# Driving Sustainability in the United States: Efficacy of State-Level Biodiesel Policy Approaches

**DOI:** 10.1021/acs.est.4c04166

**Published:** 2024-10-07

**Authors:** Adam P. Sibal, Ashlynn S. Stillwell

**Affiliations:** Civil and Environmental Engineering, University of Illinois Urbana−Champaign, 205 N Mathews Ave, MC-250, Urbana Illinois 61801, United States

**Keywords:** Biodiesel, Policy Analysis, Generalized Linear Model, Mandates, Incentives

## Abstract

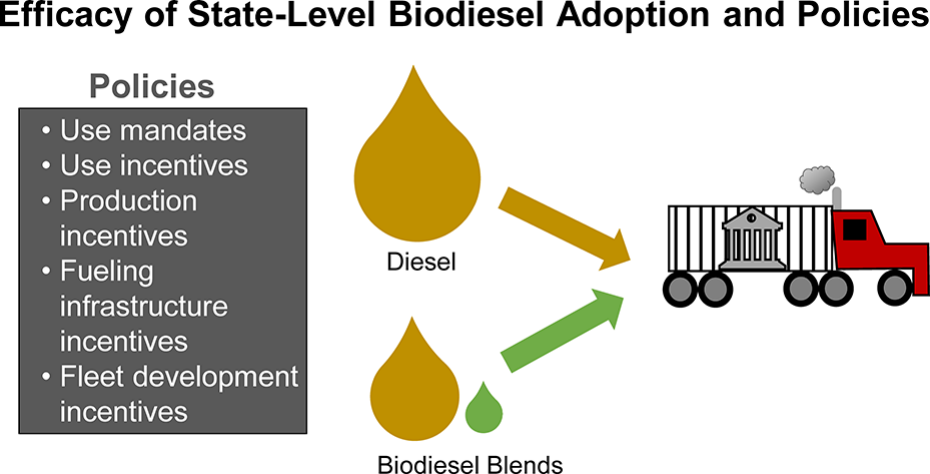

The United States has significant greenhouse gas and criteria pollutant emissions that lead to global warming, human health, ozone, and smog issues, partially attributed to its diesel-consuming transport fleet. Until fleet electrification reaches cost parity with internal combustion engines, biodiesel use can reduce these negative impacts. In this study, we analyzed and categorized the biodiesel-supporting policies of each U.S. state using manual inductive coding to compare them against state-level biodiesel consumption and production. Through statistical modeling, we determined the efficacy of these policy approaches. The policy analysis identified that biodiesel policies that support infrastructure development and biodiesel production correlate significantly with increased biodiesel consumption at the state level. We also show that a combination of these policy categories correlates significantly with overall higher biodiesel consumption. Our methodological approach and policy analysis findings reveal valuable insight into the efficacy and outcomes from existing biofuel policies in the United States.

## Abstract 2

This study identifies effective state-level biodiesel policies that enhance fuel consumption, contributing to reduced emissions and environmental sustainability.

## Introduction

The transport sector is not on track to meet the targets of the Paris Agreement to limit global warming to 1.5 °C.^1^ Heavy-duty (HD) trucks and buses, primarily fueled by diesel, make up 8% of all vehicles on the road but account for more than 35% of the direct carbon dioxide (CO_2_) emissions from the road transport sector, and these emissions are growing.^2^ In the United States, the transport sector contributed 29% of the nation’s greenhouse gas (GHG) emissions in 2021 alone.^3,4^ The transport sector is also a significant contributor of criteria emissions pollutants that lead to smog, ground-level ozone pollution, and adverse human and environmental health impacts.^3,4^ In 2020, the U.S. transport sector was responsible for 45% of all nitrogen oxide (NOx) pollution and nearly 10% of all volatile organic compounds (VOC) and particulate matter (PM_2.5_ and PM_10_), further exacerbating these phenomena.^5^

Diesel-powered fleets could alleviate some of these emissions concerns through vehicle electrification coupled with a low-carbon energy grid.^6^ However, electric HD and medium-duty (MD) trucks are not expected to reach cost parity with comparable diesel vehicles until 2035.^7^ Nationally, the electrification of the vehicles in this sector is expected to grow to 7% of the fleet by 2030, then to 49% and 80% by 2040 and 2050, respectively.^7^ As the fleet electrifies, however, the adoption of electric vehicles is expected to slow after 2050, due to the more than 30-year life of diesel-powered vehicles purchased up to and after 2035.^8^

Until electrification of the fleet becomes a reality, U.S. states could lower their transport sector direct emissions and alleviate smog, ozone, and GHG impacts in the near term by integrating biodiesel blends into their fuel mix, primarily through policy intervention and helping to create a market for these fuels.^9^ Evidence of the potential to lower full life-cycle emissions of CO_2_, PM, carbon monoxide (CO), VOCs, NOx, sulfur oxides (SOx), and methane (CH_4_) has been demonstrated by Argonne National Laboratory^10,11^ and other recent studies^12−17^ of ultralow sulfur diesel compared to soybean-based biodiesel. Notably, biodiesel can reduce greenhouse gas emissions by up to 94% over its full life cycle compared to an equivalent amount of fossil fuel diesel.^11^ Direct tailpipe emissions reductions from biodiesel use have also been shown empirically by numerous studies.^18−22^ A case study of biodiesel utilization using Argonne’s Greenhouse Gases, Regulated Emissions, and Energy Use in Transportation model is presented in Supporting Information (SI) 3 as an example of policy-informed analysis applied to Colorado, which suffers from significant ozone and smog pollution^23,24^ and has shown interest in policy levers to combat these issues.^25,26^

The importance of policy implementation to realizing the environmental, economic, and social benefits of biodiesel consumption has been demonstrated in case studies globally^27^ and in the United States.^28^ While the U.S. Renewable Fuel Standard (RFS) at the national level includes biodiesel fuel use targets by total volume, the average biodiesel content in the overall U.S. fuel mix remains low at around 2.5%.^29^ The U.S. RFS has also traditionally focused on first-generation biofuels like corn grain ethanol, shown to be (at best) carbon neutral due to land use change and other deleterious impacts on water consumption, quality, and biodiversity.^15,30−35^ Despite there being both positive and negative impacts of biofuel use, U.S. states can make more intimate policy decisions around these fuels based on specific needs, concerns, and goals.

Many U.S. states have implemented policies that aim to support the consumption and production of biodiesel through various policy mechanisms. However, these policy mechanisms vary widely in their approach and structure. States have instituted biodiesel use mandates, tax incentives, and rebates; biodiesel production incentives; fueling infrastructure development; and/or biodiesel end-use expansion through fleets. Even with these varied approaches, the structures also vary with mandates or incentives that apply either statewide, to government entities, to companies, to nonprofits, to biodiesel producers, to biodiesel fuel blenders, and/or to retailers. The complexity of the current policy landscape and the wide range of options that a state can select to increase the use of biodiesel add a layer of complexity for legislative bodies to discern effective approaches to both increase biodiesel fuel use and lower costs to consumers. Several studies have analyzed the current global^36^ and U.S. biodiesel policy landscape,^37^ while others have focused on particular policy measures such as biodiesel production incentives.^38^

To complement these analyses, we conducted a comprehensive analysis of U.S.-based biodiesel policies. We present the statistical correlation between these policies to both biodiesel consumption and production through generalized linear models (GLMs) to derive their efficacy. GLMs allow for modeling the effect that an independent variable (a policy) has on a dependent variable (biodiesel consumption and production),^39^ are particularly useful for non-normal distributions and categorical data,^40^ and have been utilized in other areas of policy analysis in the literature.^41−43^ Through this work, we aim to help strengthen the use of evidence in policy-making decisions around biodiesel, with similar parallels applicable to other transport sector decarbonization mechanisms.

## Materials and Methods

To assess the landscape of biodiesel-supporting policy options in each U.S. state, we analyzed biodiesel policy language in the U.S. Department of Energy’s Alternative Fuels Data Center Federal and State Laws and Incentives search tool.^44^ We reviewed and categorized the 325 results using manual inductive coding to discern the policy category and subcategory definitions shown in Table 1. Full results of the manual inductive coding can be found in SI 2.

**Table 1:** Major U.S. Biodiesel-Supporting Policy Mechanisms with Categorical and Subcategorical Definitions

major policy category	definition	subcategories
biodiesel fuel use mandate	legislation that defines the volume or volume percentage of biodiesel that must be sold in the diesel fuel mix in the state for designated vehicles; includes renewable fuel standard programs	statewide
		government entities
biodiesel fuel use incentive	any tax reduction or tax rebate that supports the use of biodiesel or its blends in the state over fossil fuel-based diesel	statewide
		government entities
		consumers
		retailers
		blenders
		distributors
*biodiesel production incentive*	any tax reduction, tax rebate, grant, or loan program that supports the production of biodiesel fuel directly in the state	no subcategory identified
biodiesel fueling infrastructure incentive	any tax reduction, tax rebate, grant, loan program, or mandate that supports the development of biodiesel fueling infrastructure directly in the state; not limited to blending equipment, tanks, and fuel dispensing equipment	government entities
		companies
		nonprofits
		consumers
biodiesel fleet development incentive	any tax reduction, tax rebate, grant, loan program, or mandate that supports the development of biodiesel and its fuel blends for fleet use within the state; not limited to vehicle conversion or the purchase of vehicles compatible with blends greater than B20	government entities
		companies
		nonprofits
		consumers

Data from the U.S. Energy Information Administration’s (EIA) State Energy Data System (SEDS): 1960–2020 were used to estimate state-level biodiesel consumption and production in 2020.^45,46^ To account for differences in state populations that might lead to increased biodiesel consumption, we normalized consumption on a per capita basis based on Census data. The state-level per capita biodiesel consumption and total biodiesel production volumes in 2020 were then compared with biodiesel-supporting policies (Table 1).

A state-level policy maker’s intent with enacting any supporting biodiesel policy would inherently be to increase the use of biodiesel. To determine which individual policy approaches are most strongly correlated with higher levels of biodiesel use, we developed and tested multiple GLMs for their best fit, where the response variable analyzed was 2020 per capita biodiesel consumption (gal/person). To do so, we first modeled every possible combination of major policy categories (i.e., all five, varied combinations of four, etc.) with varied distribution assumption types (Gaussian, Poisson, gamma, binomial) to determine the best fit in terms of the lowest Akaike information criterion (AIC) and Bayesian information criterion (BIC) values across 124 model combinations. To provide additional information to policy makers on policy implementation in practice, we also modeled every combination of subcategories (i.e., all 17 including production incentives and individual subcategories) with the same distribution assumption options, creating over 8 million different model combinations. The models with the lowest absolute value sum of the AIC and BIC numbers were determined to be the best fitting models and are discussed herein.

Additionally, we explored a log transformation of the response variable to address potential skewness in the biodiesel consumption data. This transformation provides additional insight into understanding the role of certain policy incentives.

## Results and Discussion

In this section, we first present the results of the best fitting major policy and subcategory GLMs and discuss the key findings. We conclude this section with a detailed discussion of the major policy categories and the significance of the subcategories of the policies within them.

### Major and Subcategory Policy GLM Comparisons

The analysis indicated clearly that the best model family for the data set was Poisson with the lowest AIC/BIC number, as summarized in Table S1.1. The results of the best fitting major and subcategory policy GLMs including their coefficient estimates, *p*-values, and 95% confidence intervals are shown in Table 2 and are further detailed in Tables S1.2 and S1.3.

**Table 2:** Coefficient Estimates, 95% Confidence Intervals, and *p*-Values for the Best Fitting Major Policy Category and Subcategory Policy Generalized Linear Models (GLMs) where the Response Variable Analyzed Was 2020 per Capita Biodiesel Consumption (Gal/Person) with Akaike Information Criterion (AIC) and Bayesian Information Criterion (BIC) for Each Model^a^

		major policy category GLM		subcategory policy GLM	
policy category		coefficient estimate (95% confidence interval)	*p*-value	coefficient estimate (95% confidence interval)	*p*-value
biodiesel fuel use mandate		0.1750 (−0.062 to 0.412)	0.148	-	-
	statewide	-	-	0.0855 (−0.352 to 0.523)	0.702
	government entities	-	-	–0.0842 (−0.472 to 0.303)	0.670
biodiesel fuel use incentive		0.0762 (−0.172 to 0.325)	0.547	-	-
	statewide	-	-	0.0817 (−0.603 to 0.766)	0.815
	government entities	-	-	**–1.2722 (−2.000 to −0.544)**	**0.001**
	consumers	-	-	–0.0127 (−0.586 to 0.561)	0.965
	retailers	-	-	–0.0234 (−0.586 to 0.539)	0.935
	blenders	-	-	0.2400 (−0.399 to 0.879)	0.462
	distributors	-	-	–0.0165 (−0.603 to 0.570)	0.956
biodiesel production incentive		**0.7192 (0.469 to 0.970)**	**0.000**	**0.5540 (0.198 to 0.910)**	**0.002**
biodiesel fueling infrastructure incentive		**0.3075 (0.004 to 0.611)**	**0.047**	-	-
	government entities	-	-	**–1.6713 (−2.847 to −0.495)**	**0.005**
	companies	-	-	**0.9619 (0.391 to 1.533)**	**0.001**
	nonprofits	-	-	1.2078 (−0.302 to 2.718)	0.117
	consumers	-	-	–1.1697 (−2.707 to 0.367)	0.136
biodiesel fleet development incentive		–0.0502 (−0.319 to 0.219)	0.714	-	-
	government entities	-	-	**0.5184 (0.100 to 0.937)**	**0.015**
	companies	-	-	**–0.6896 (−1.243 to −0.136)**	**0.015**
	nonprofits	-	-	0.0496 (−0.559 to 0.659)	0.873
	consumers	-	-	0.6312 (−0.438 to 1.700)	0.247

AIC	332.875	301.174
BIC	–26.810	–35.330

a Significant policy types (*p*-value ≤ 0.05) are bolded.

Interestingly, the model with the best fit in both cases was the model that retained all the policy categories. However, upon log-transforming the response variable in the major policy category GLM, we found that the best fitting models included only production and infrastructure incentives or some combination including the two policy types. This result further validated the findings of the major policy category GLM, where these incentives clearly correlated to higher biodiesel consumption. As such, we discuss these findings briefly here and provide the full log-transformed model details in the Tables S1.5 and S1.6.

Given the complexity and potential for overfitting in the subcategory model, we focused our analysis on 2020 per capita biodiesel consumption (i.e., the untransformed response) to provide more interpretable results that are grounded in the actual data.

The results of the best fitting major policy category GLM indicate that biodiesel production incentive policies and biodiesel fueling infrastructure policies both have a positive coefficient estimate with per capita biodiesel consumption and demonstrate significance. This model also shows near zero coefficient estimates and limited significance among mandates, fuel use incentive, and fleet development policies. The best fitting subcategory policy GLM demonstrates positive coefficient estimates and significance for biodiesel production incentives, biodiesel fueling infrastructure incentives when applied to companies, and biodiesel fleet development incentives when applied to government entities. The best fitting subcategory policy GLM also shows negative coefficient estimates and significance for biodiesel fuel use incentives when applied to government entities, biodiesel fueling infrastructure incentives when applied to government entities, and biodiesel fleet development incentives when applied to companies.

Between the two models, similar fitness is observed; however, the subcategory policy GLM has a slightly better overall fit with a lower AIC value and a slightly higher BIC value. A commonality exists between the two models, in which both indicate a high significance for biodiesel production incentive policies and biodiesel fueling infrastructure incentive policies associated with higher levels of biodiesel fuel consumption per capita. This commonality suggests that these two policy types might be important areas of focus when considering the breadth of policy options available. However, when considering fueling infrastructure incentives, the subcategory policy GLM indicates that policies that apply the incentive to companies that are setting up such infrastructure, such as fuel stations, tend to correlate positively with higher consumption levels per capita. Only applying the policies to fuel infrastructure for government owned vehicles/entities might not be as effective.

Another commonality between the models is the lack of significance shown for biodiesel fuel use mandates and higher levels of biodiesel consumption per capita. Additionally, between the two models, there is not a significant positive relationship between biodiesel fuel use incentive policies and higher per capita biodiesel consumption. For this policy approach, the only significant policy type is observed through government entities, and the coefficient estimate is negative with high significance (*p*-value = 0.001), suggesting these policies might be ineffective in increasing per capita biodiesel consumption.

An interesting difference between the models arises for biodiesel fleet development policies, in which the major policy category GLM exhibits little significance with a higher level of biodiesel fuel use per capita, while the subcategory policy GLM shows significance at *p*-value = 0.015 for government applicable policies with a slightly positive coefficient estimate and company applicable policies with a negative coefficient estimate. This result suggests that fleet development policies should be considered closely and might require local context considerations for policy makers.

Collectively, the results of these two models indicate that biodiesel fuel use mandates, biodiesel fuel use incentive policies, and biodiesel fleet incentive policies might not significantly contribute to increased biodiesel fuel use. Given the likely high cost to the governing body of fuel use incentive policies and fleet incentive policies, governing bodies could more effectively allocate funding toward biodiesel fueling infrastructure-based or production-based policies as the most significant, positively correlated policy mechanism to increase per capita biodiesel fuel use.

### Major Policy Category Assessment

**Figure 1:**
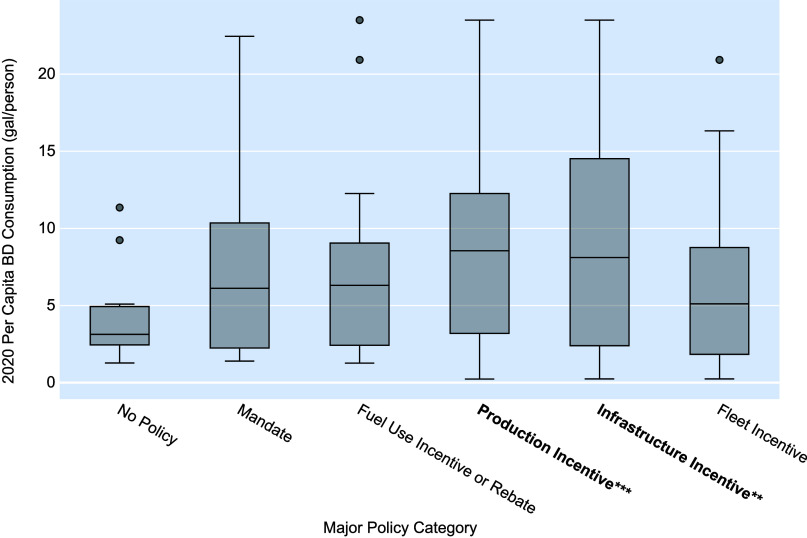
Major biodiesel (BD) policy category comparisons with 2020 per capita biodiesel consumption. Significance corresponding to the major policy categories in the GLM are noted as follows: * for *p*-value < 0.10, ** for *p*-value < 0.05, and *** for *p*-value < 0.01.

While it is valuable to understand the policies that most significantly contribute to higher per capita biodiesel consumption, policies around biodiesel can be implemented alone or in combination for various reasons to overcome state-specific challenges with its adoption and increased use. It is therefore important to consider different policy approaches and evaluate their efficacy. Figure 1 shows the results of the major policy category GLM, along with a graphical representation of biodiesel consumption per capita when focusing solely on the major policy categories.

An additional finding of the modeling suggests that a combination of multiple major policy category approaches correlates with higher levels of per capita biodiesel consumption. Four of the five major policy categories in Table 2 displayed positive coefficients in the GLM, indicating the potentially positive effect of a multipolicy approach. Visual data representations of this effect can be seen in Figure S1.4.

### Biodiesel Fuel Use Mandates

Our analysis of biodiesel-supporting policies indicated that 17 states currently have biodiesel fuel use mandates with varied minimum biodiesel blend requirements. Of these 17 states, 11 were found to apply to only vehicles and/or equipment owned, leased, or operated by the state in some capacity, while six applied to all diesel fuel sold statewide. Full details on minimum blending requirements and conditions can be found in Table S2.1.

**Figure 2:**
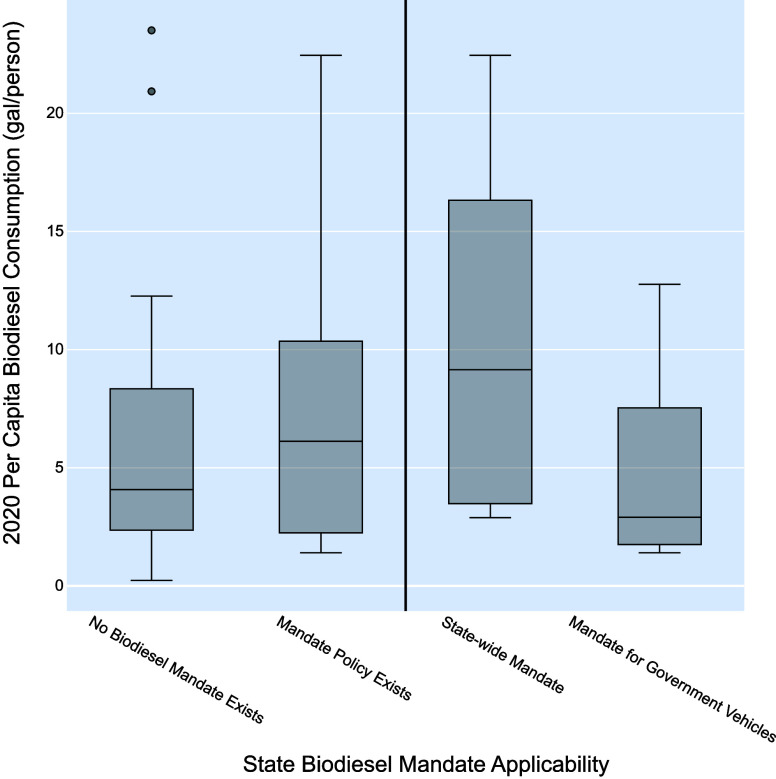
Biodiesel use mandate policy comparisons with 2020 per capita biodiesel consumption. Significance corresponding to the subcategory policy GLM are noted as follows: * for *p*-value < 0.10, ** for *p*-value < 0.05, and *** for *p*-value < 0.01. *p*-values for the major category (left of the vertical line) come from the major policy category GLM, while subcategories (right of the vertical line) come from the biodiesel fuel use mandate policy subcategory GLM.

Figure 2 demonstrates that in states where a biodiesel fuel use mandate exists, per capita biodiesel consumption tends to be higher than in states without a mandate. However, from a major policy approach, the correlation is not statistically significant. A further assessment of biodiesel fuel use mandate policies by subcategory indicates that a statewide mandate on all fuels sold in the state has a slightly positive correlation with greater per capita biodiesel consumption, though it is insignificant at *p*-value = 0.720, while mandates that apply to vehicles and/or equipment owned, leased, or operated by the government only (*p*-value = 0.670) do not significantly outperform states with no biodiesel fuel use mandates at all. These results additionally indicate that biodiesel mandate policy approaches might not be effective mechanisms for increasing biodiesel consumption, as they do not significantly correlate with higher levels of biodiesel use. State-level context is necessary when considering such policies.

Direct consultation with members of the Colorado Motor Carriers Association and the Colorado Fuel Distributors Association indicated that drawbacks with a mandate approach include locking in fuel consumers to higher fuel prices. However, the benefits of this policy approach include certainty for governments in achieving emissions reduction targets and lower government fiscal responsibilities than in other incentive policy structures.^47^

### Biodiesel Fuel Use Incentives

State-level biodiesel fuel use incentive structures exist in 15 U.S. states. Of these policies, four states remove sales tax on biodiesel for government vehicle purchases, and 12 states have policies that provide biodiesel fuel use incentives more broadly across the state. Six states offer a decreased tax rate at the pump or rebate to consumers. Five states offer a tax rebate to retailers. Three states provide incentives to distributors of biodiesel and biodiesel blended fuels, and three states provide incentives to biodiesel fuel blenders. Full details of the state policies are given in Table S2.2.

**Figure 3:**
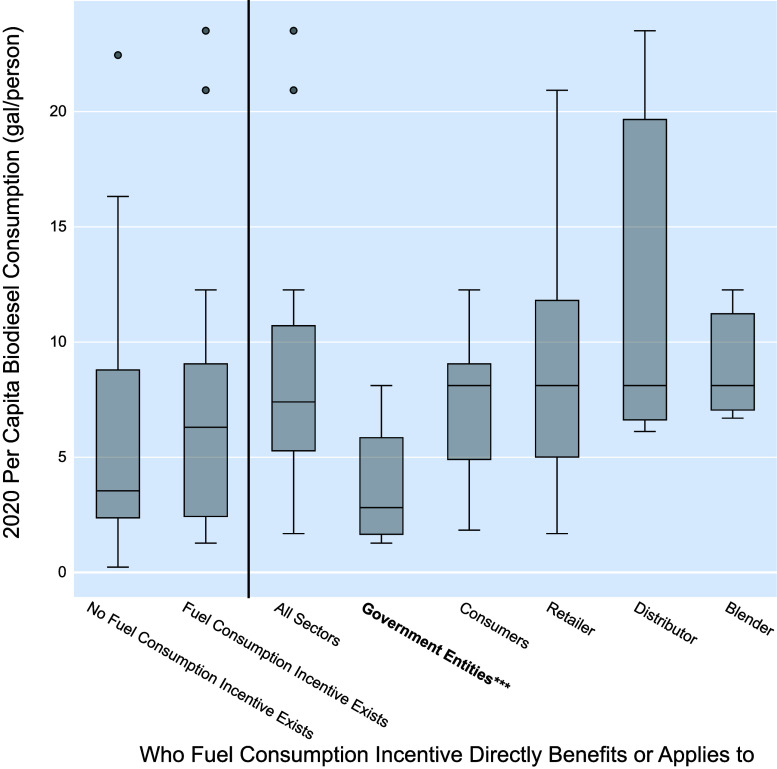
Biodiesel fuel use incentive policy comparisons with 2020 per capita biodiesel consumption. Significance corresponding to the subcategory policy GLM are noted as follows: * for *p*-value < 0.10, ** for *p*-value < 0.05, and *** for *p*-value < 0.01. *p*-values for the major category (left of the vertical line) come from the major policy category GLM, while subcategories (right of the vertical line) come from the biodiesel fuel use incentive policy subcategory GLM.

Analysis of these policies, shown in Figure 3 and Table 2, indicates that biodiesel fuel use incentive policies do not significantly lead to more biodiesel consumption on a per capita basis. However, further analysis of these policy subcategories shows the highest positive correlation between the direct recipient of the incentive as the distributor and increased biodiesel consumption (coefficient estimate = 0.2400), although it is not statistically significant (*p*-value = 0.462). Conversely, the subcategory policy GLM indicates that incentive policies applicable to only government entities exhibit a highly significant (*p*-value = 0.001) but negative correlation (coefficient estimate = −1.2722) with per capita biodiesel consumption, indicating that government focused incentive policies are associated with decreases in per capita biodiesel consumption. The GLM results demonstrate broadly that biodiesel fuel use incentive-based policies do not significantly contribute to per capita biodiesel fuel consumption, suggesting that this state-level policy should be carefully considered prior to implementation.

Despite this lack of statistical significance, direct consultation with members of the Colorado Motor Carriers Association and the Colorado Fuel Distributors Association indicated that fuel use incentive policies are preferred by consumers of diesel fuel, as they remove some of the potential added cost in instances where biodiesel blends are more expensive than fossil fuel diesel and shelter consumers from some of the risks of higher biodiesel fuel prices. Incentives also have the potential to decrease overall costs to consumers for biodiesel and biodiesel blended fuels.^48^

Potential drawbacks of this policy approach include not meeting emissions targets if the incentive structure does not support an increase in biodiesel use due to market conditions,^49^ as shown as a possible outcome in this analysis. There can also be uncertainty for governments in the financial costs required if biodiesel consumption fluctuates. Another drawback of this approach is the inherent fiscal burden on the governing body and an incentive structure that could lead to the direct recipients capturing the incentive value and not passing the cost savings on to the consumer.^50^

### Biodiesel Production Incentives

Policy options that have been implemented in several states include measures such as property tax exemptions for biodiesel production facilities, tax rebates for jobs created by biodiesel facilities, exemption from sales tax for capital equipment and services, grants and low-interest loans for facility construction, and sales tax exemptions on feedstocks.^38^ As of 2022, 33 states currently produce biodiesel, up from 30 states in 2020. Of these 33 states, 11 have policies in place that incentivize biodiesel production in the state.

As shown in the results of the GLMs (Table 2 and Figure 1), biodiesel production policies are significantly correlated to higher per capita biodiesel consumption at the state level. We explored this relationship with an ordinary least-squares (OLS) model (full results shown in Figure S1.1), revealing a positive and significant correlation (*R*^2^ = 0.292, *p*-value = 0.00) between per capita biodiesel production in a state and higher levels of per capita biodiesel consumption. Although the relationship is relatively weak, the correlation is significant, implying that states with higher biodiesel production levels tend to have higher biodiesel consumption. The reason for the weak relationship could be attributed to the limited observations and 39 states that produced less than 2 million barrels of biodiesel and also consumed less than 1 million barrels in 2020. There are a few states that are large consumers with low production, and others that are large producers with low consumption, likely contributing to the low *R*^2^ value. Twenty states also register no production, as shown in the map in Figure S1.2. The largest biodiesel producers by volume tend to be in the Midwest region, close to ample feedstocks, such as soybean, canola, and corn oils.

However, the results of this analysis indicate that states that have enacted biodiesel production incentive policies tend to correlate significantly with increased biodiesel consumption per capita, which is also correlated to production in those states. State policymakers aiming to increase biodiesel consumption should consider production incentive policy mechanisms that might lead to an increased level of production and consumption of biodiesel in their state.

Benefits of incentivizing and securing in-state biodiesel production include potentially lower fuel cost and reduced life-cycle emissions of biodiesel fuel due to decreased transportation requirements compared to procuring biodiesel from surrounding producer states.^37,51^ An additional benefit of this approach is job creation, supporting the construction and operation of these production facilities.^52^

### Biodiesel Fueling Infrastructure Incentives

Biodiesel fueling infrastructure incentive policies are in 14 states, which were further categorized to identify the direct recipient of the incentive. Of these states, six states have policies that provide incentives to government entities, thirteen states provide incentives to companies, four states provide incentives to nonprofit organizations, and two states provide incentives to small-scale consumers. Examples of such policies include tax credits for equipment and labor costs for the purchase and installation of alternative fuel infrastructure; grants for the purchase of biodiesel blending equipment, storage, and dispensing; and tax credits or grants for equipment and facility conversions to blend, store, and dispense biodiesel fuels. For a detailed list of the policies, see Table S2.4.

**Figure 4:**
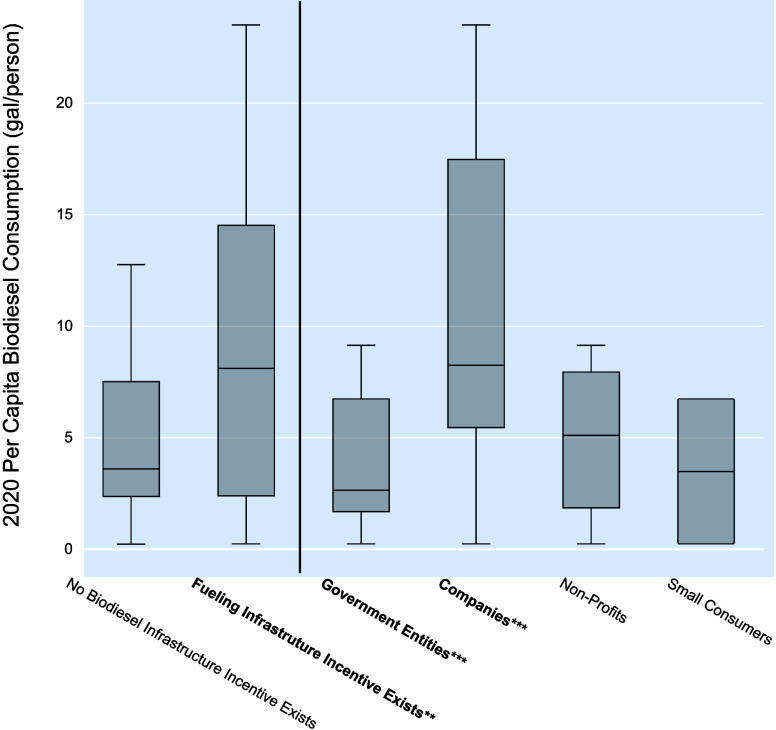
Biodiesel fueling infrastructure incentive policy comparisons with 2020 per capita biodiesel consumption Significance corresponding to the subcategory policy GLM are noted as follows: * for *p*-value < 0.10, ** for *p*-value < 0.05, and *** for *p*-value < 0.01. *p*-values for the major category (left of the vertical line) come from the major policy category GLM, while subcategories (right of the vertical line) come from the biodiesel fueling infrastructure incentive policy subcategory GLM.

An analysis of these policies’ existence in states compared to their per capita biodiesel consumption shows an association between the existence of biodiesel fueling infrastructure policies and increased consumption, as shown in Figure 4 and Table 2. Analyzing the direct recipient of the incentive shows a strong positive relationship between states in which the incentive is directly available to companies that blend, store, transport, or dispense biodiesel fuels and their blends with a *p*-value of 0.001 and increased biodiesel consumption. Conversely the subcategory policy GLM indicates a negative relationship between the application of fueling infrastructure policy only to government entities and biodiesel consumption (coefficient estimate = −1.6713; *p*-value = 0.005). For states that provide the incentive to nonprofits and small direct consumers, there is no significant difference between states with no biodiesel fueling infrastructure incentives in terms of increased per capita biodiesel consumption. The biodiesel fueling infrastructure analysis results imply that states that offer incentives to companies generally have higher biodiesel consumption, and such a policy might support increased consumption if adopted.

Benefits of biodiesel fueling infrastructure policies include the potential to lower direct fuel costs to consumers of biodiesel fuel and its blends by alleviating capital cost expenses for the infrastructure equipment that fuel blenders, distributors, and retailers would pass onto consumers. Additional benefits of these policies include increased availability of biodiesel and biodiesel blended fuels and lower market barriers for the distribution of these fuels.^53^

Drawbacks of this approach include the need for government expenditures to finance such projects and decreased tax revenues. Governments that implement such policies might also need to establish a procedure or acquire agency staffing to verify the installation of equipment.

### Biodiesel Fleet Development Incentives

The final policy approach we analyzed is biodiesel fleet development incentives. There are 18 states with fleet development policies, with 16 of those states providing incentives to government entities, 14 states providing to companies, nine states providing to nonprofit organizations, and four states offering incentives to all consumers. Examples of these policies include tax credits, grants, or reimbursements for all or portions of the equipment and labor cost of engine conversions to run on blends greater than B20 (20% biodiesel blended diesel fuel); tax credits, grants, or reimbursements for purchasing new vehicles that can run on blends greater than B20; or mandates for the procurement of vehicles that can run on blends greater than B20. A full list of biodiesel fleet development policies can be found in Table S2.5.

**Figure 5:**
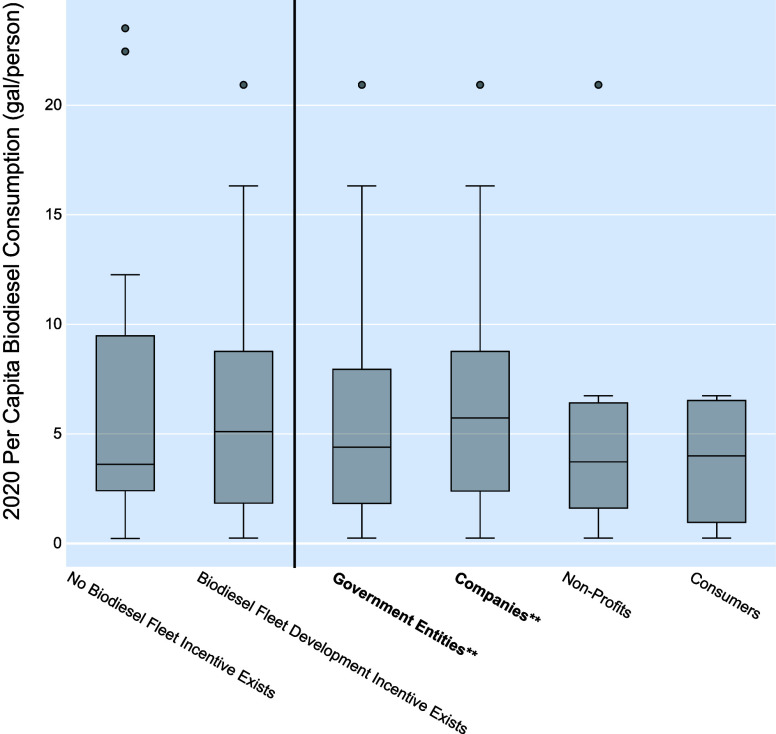
Biodiesel fleet development incentive policy comparisons with 2020 per capita biodiesel consumption. Significance corresponding to the subcategory policy GLM are noted as follows: * for *p*-value < 0.10, ** for *p*-value < 0.05, and *** for *p*-value < 0.01. *p*-values for the major category (left of the vertical line) come from the major policy category GLM, while subcategories (right of the vertical line) come from the biodiesel fleet development incentive policy subcategory GLM.

Analysis of these state-level policies and per capita biodiesel consumption does not show a strong relationship between biodiesel fleet development policies and increased biodiesel consumption. Of all the major policy approaches, fleet development incentives show the weakest significance (*p*-value = 0.714) to increased biodiesel consumption per capita, as shown in Table 2 with coefficient estimate = −0.0502, implying a weakly negative trend. However, the subcategory policy GLM indicates that both government entities (positive coefficient estimate) and company applicable policies (negative coefficient estimate) are significant indicators of biodiesel consumption per capita. This result in the subcategory policy GLM was unexpected based on the presented data in Figure 5 and could be a result of the limited set of observations or a counteractive effect of the policies in the major policy category GLM.

In theory, these policies should increase biodiesel consumption, but the effect appears to be statistically insignificant in practice from a major policy perspective. These results imply that the additional engine conversion and maintenance costs needed for engines that run on greater than B20^54^ and the greater purchase cost of alternative fuel vehicles^55^ might impede achieving the desired results such that funding could be allocated to other methods that increase biodiesel consumption.

## Policy Implications

Our study identifies key biodiesel policies that significantly correlate with per capita consumption. Policies that provide incentives for biodiesel production and fueling infrastructure, especially when directed at companies, show a strong positive correlation with higher biodiesel use. Biodiesel fuel use mandates applied statewide demonstrate some positive correlation with consumption, although their overall significance is limited. Biodiesel fuel use incentive policies do not show a significant impact on increasing biodiesel consumption and are particularly ineffective when directed at government entities. Fleet development incentives also fail to significantly boost biodiesel consumption with mixed results depending on the targeted group.

In conclusion, the most effective policies for increasing biodiesel consumption are those that support production and incentivize the fueling infrastructure through companies. Policymakers should focus resources on these areas to achieve more substantial and sustained increases in the level of biodiesel use.

The results of our analysis present several key findings relevant to transportation sector policies to alleviate smog and ozone issues, decrease human health impacts, and increase biodiesel fuel consumption as a near-term solution, as the transition to electrification of the MD and HD transportation sectors is a more medium- to long-term possibility. This analysis demonstrates the context of biodiesel policies in supporting sustainable transportation transitions; however, policymakers should not only be cognizant of air pollution emissions reductions from the transport sector but also consider the local- and broader-scale context of land use change, biodiversity, and water impacts of first-generation biofuels.^15,30−35^ As shown, state-level policy around biofuels can have a significant impact on the use of these fuels and contribute to sustainable transport solutions.

## Supporting Information Available

GLM modeling outputs, biodiesel production and consumption, policy maps, U.S. biodiesel policies sorted into manual inductive coding categories, and Colorado biodiesel use case study (PDF)

## Abbreviations

CO_2_: carbon dioxide
PM: particulate matter
CO: carbon monoxide
VOCs: volatile organic compounds
NOx: nitrogen oxides
SOx: sulfur oxides
CH_4_: methane
PM_2.5_: particulate matter ≤2.5 μm in diameter
PM_10_: particulate matter ≤10 μm in diameter
GHG: greenhouse gas
MD: medium-duty
HD: heavy-duty
LD: light-duty
NREL: National Renewable Energy Laboratory
BD: biodiesel
B20: 20% biodiesel blended diesel fuel
B100: 100% biodiesel
EIA: U.S. Energy Information Administration
SEDS: State Energy Data System
