# Supplementary material for: Driving Sustainability in the United States: Efficacy of State-Level Biodiesel Policy Approaches
Source: Environ Sci Technol. 2024 Oct 7;58(42):18474–83. doi: 10.1021/acs.est.4c04166 (PMC11500407; doi:10.1021/acs.est.4c04166)
Supplement: Supplementary file 1 — es4c04166_si_001.pdf [file es4c04166_si_001.pdf]

Supporting Information: Driving Sustainability in the United States: Efficacy of State-Level Biodiesel Policy Approaches

AUTHOR NAMES: Adam P. Sibal, Ashlynn S. Stillwell*

AUTHOR ADDRESS: Civil and Environmental Engineering, University of Illinois Urbana-Champaign, 205 N Mathews Ave, MC-250, Urbana IL 61801, adamps2@illinois.edu

CONTENTS

SI 1: GLM modeling outputs, biodiesel production, consumption, and policy maps

Pages: S2-S13

Figures: 5

Tables: 6

SI 2: U.S. biodiesel policies sorted into manual inductive coding categories

Pages: S14-S43

Figures: 0

Tables: 5

SI 3: Colorado Biodiesel Use Case Study

Pages: S44-S85

Figures: 49

Tables: 8

Supporting Information 1: Driving Sustainability in the United States: Efficacy of State-Level Biodiesel Policy Approaches

AUTHOR NAMES: Adam P. Sibal, Ashlynn S. Stillwell*

AUTHOR ADDRESS: Civil and Environmental Engineering, University of Illinois Urbana-Champaign, 205 N Mathews Ave, MC-250, Urbana IL 61801, adamps2@illinois.edu

Table S1.1. Summary of absolute value of the combined AIC/BIC value for the best performing major policy category GLM by model family.

Model Family	Best Preforming GLM AIC/BIC Absolute Value Sum
Poisson	356.685
Gamma	429.424
Gaussian	1,239.251
Binomial	45,550.02

Table S1.2. The best fitting major policy category GLM model outputs for biodiesel production per capita against all policy types under a Poisson distribution for U.S. states in 2020.

Generalized Linear Model Regression Results

Dep. Variable:	2020 Per Capita BD Consumption (gal/person)	No. Observations:	51
Model:	GLM	Df Residuals:	45
Model Family:	Poisson	Df Model:	5
Link Function:	log	Scale:	1.0000
Method:	IRLS	Log-Likelihood:	-160.44
Date:	Wed, 10 Jul 2024	Deviance:	150.12
Time:	14:39:44	Pearson chi2:	147.
No. Iterations:	5		
Covariance Type:	nonrobust		

	coef	std err	z	P>|z|	[0.025	0.975]
const	1.3381	0.110	12.128	0.000	1.122	1.554
Mandate	0.1750	0.121	1.448	0.148	-0.062	0.412
Fuel Use Incentive or Rebate	0.0762	0.127	0.602	0.547	-0.172	0.325
Production Incentive	0.7192	0.128	5.627	0.000	0.469	0.970
Infrastructure Incentive	0.3075	0.155	1.985	0.047	0.004	0.611
Fleet Incentive	-0.0502	0.137	-0.366	0.714	-0.319	0.219

Table S1.3. The best fitting subcategory policy GLM model outputs for biodiesel production per capita against all policy types under a Poisson distribution for U.S. states in 2020.

Generalized Linear Model Regression Results

Dep. Variable:	2020 Per Capita BD Consumption (gal/person)	No. Observations:	51
Model:	GLM	Df Residuals:	30
Model Family:	Gamma	Df Model:	20
Link Function:	log	Scale:	0.61728
Method:	IRLS	Log-Likelihood:	-130.15
Date:	Thu, 18 Apr 2024	Deviance:	23.952
Time:	23:28:08	Pearson chi2:	18.5
No. Iterations:	26		
Covariance Type:	nonrobust		

	coef	std err	z	P>|z|	[0.025	0.975]
const	1.4893	0.216	6.894	0.000	1.066	1.913
Mandate	0.5720	1.021	0.560	0.575	-1.429	2.573
Mandate_state	-0.3222	0.851	-0.379	0.705	-1.991	1.346
Mandate_Gov	-0.6782	0.979	-0.692	0.489	-2.598	1.241
Fuel_Use_Incentive_or_Rebate	0.1037	2.044	0.051	0.960	-3.902	4.109
FU_State	0.2080	2.783	0.075	0.940	-5.247	5.663
FU_Gov	-0.9802	1.977	-0.496	0.620	-4.855	2.894
FU_Consumer	-0.2519	0.828	-0.304	0.761	-1.875	1.372
FU_Retailer	0.0824	0.828	0.100	0.921	-1.540	1.705
FU_Distributor	-0.0990	1.000	-0.099	0.921	-2.058	1.860
FU_Blender	0.1840	0.886	0.208	0.835	-1.552	1.920
Production_Incentive	0.4341	0.352	1.234	0.217	-0.255	1.124
Infrastructure_Incentive	-0.6089	0.611	-0.996	0.319	-1.807	0.589
INF_Gov	-1.1189	0.455	-2.461	0.014	-2.010	-0.228
INF_Companies	1.2817	0.576	2.225	0.026	0.153	2.411
INF_NP	0.7718	0.796	0.969	0.332	-0.789	2.332
INF_Consumers	-1.0001	1.456	-0.687	0.492	-3.854	1.854
Fleet_Incentive	-0.0135	1.029	-0.013	0.990	-2.030	2.003
FT_Gov	0.4569	0.912	0.501	0.616	-1.330	2.244
FT_NP	-0.2446	0.635	-0.385	0.700	-1.489	1.000
FT_Companies	-0.4602	0.706	-0.652	0.515	-1.844	0.924
FT_Consumers	0.6977	0.990	0.705	0.481	-1.243	2.639

AIC: 302.30846931650257
BIC: -94.0028419701292

Table S1.4. Biodiesel consumption per capita and biodiesel production per capita OLS model outputs for U.S. states in 2020.

```
                            OLS Regression Results
==========================================================================================
Dep. Variable:     Log_BD_Consumption_per_Capita   R-squared:                       0.292
Model:                                       OLS   Adj. R-squared:                  0.277
Method:                            Least Squares   F-statistic:                     20.16
Date:                           Sun, 21 Apr 2024   Prob (F-statistic):           4.33e-05
Time:                                   00:53:46   Log-Likelihood:                -46.558
No. Observations:                             51   AIC:                             97.12
Df Residuals:                                 49   BIC:                             101.0
Df Model:                                      1
Covariance Type:                       nonrobust
======================================================================================
                      coef    std err          t      P>|t|      [0.025      0.975]
--------------------------------------------------------------------------------------
const               1.5419      0.096     16.016      0.000       1.348       1.735
Log_BD_Production   1.4510      0.323      4.490      0.000       0.802       2.100
==============================================================================
Omnibus:                        0.524   Durbin-Watson:                   1.788
Prob(Omnibus):                  0.769   Jarque-Bera (JB):                0.667
Skew:                           0.179   Prob(JB):                        0.716
Kurtosis:                       2.569   Cond. No.                         3.82
==============================================================================
```

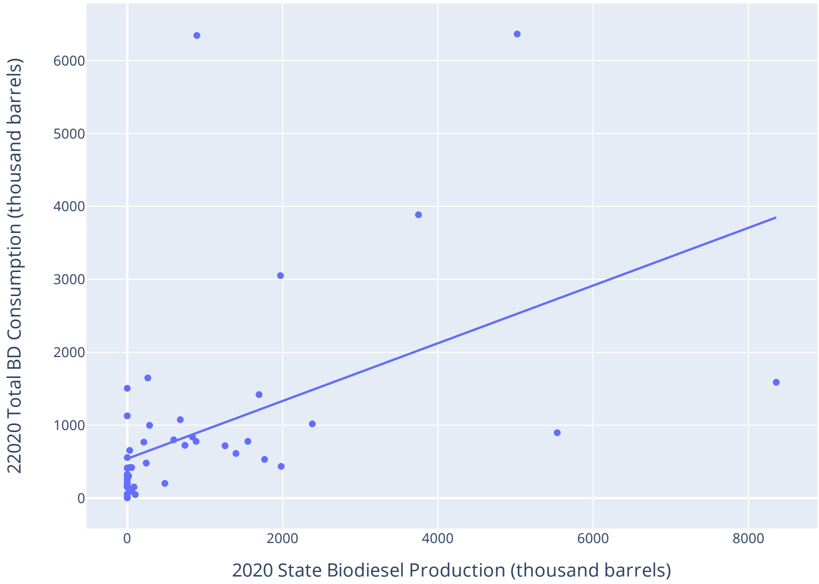

Figure S1.1. 2020 state-level biodiesel production and per capita biodiesel consumption (R^2 = 0.24).[2]

Table S1.5. Top 5 best fitting major policy category model GLM where the log-transformed response variable analyzed was 2020 per capita biodiesel consumption (gal/person).

Ranking of Model Fit	Major Policy Category in the Model	AIC	BIC	AIC/BIC Absolute Value Sum
1	Production Incentive	146.48	44.53	191.00
2	Infrastructure Incentive	148.86	46.91	195.76
3	Production Incentive, Mandate	147.80	48.18	195.98
4	Production Incentive, Fuel Use Incentive	147.98	48.31	196.29
5	Production Incentive, Infrastructure Incentive	148.23	48.11	196.33

Table S1.6. Best fitting major policy category model GLM with log-transformed response variable analyzed was 2020 per capita biodiesel consumption (gal/person).

Generalized Linear Model Regression Results

Dep. Variable:	2020 Per Capita BD Consumption (gal/person)	No. Observations:	51
Model:	GLM	Df Residuals:	49
Model Family:	Poisson	Df Model:	1
Link Function:	log	Scale:	1.0000
Method:	IRLS	Log-Likelihood:	-71.238
Date:	Tue, 03 Sep 2024	Deviance:	237.19
Time:	12:59:45	Pearson chi2:	29.4
No. Iterations:	5		
Covariance Type:	nonrobust		

	coef	std err	z	P>|z|	[0.025	0.975]
const	0.1842	0.159	1.160	0.246	-0.127	0.495
Production Incentive	0.4496	0.234	1.923	0.055	-0.009	0.908

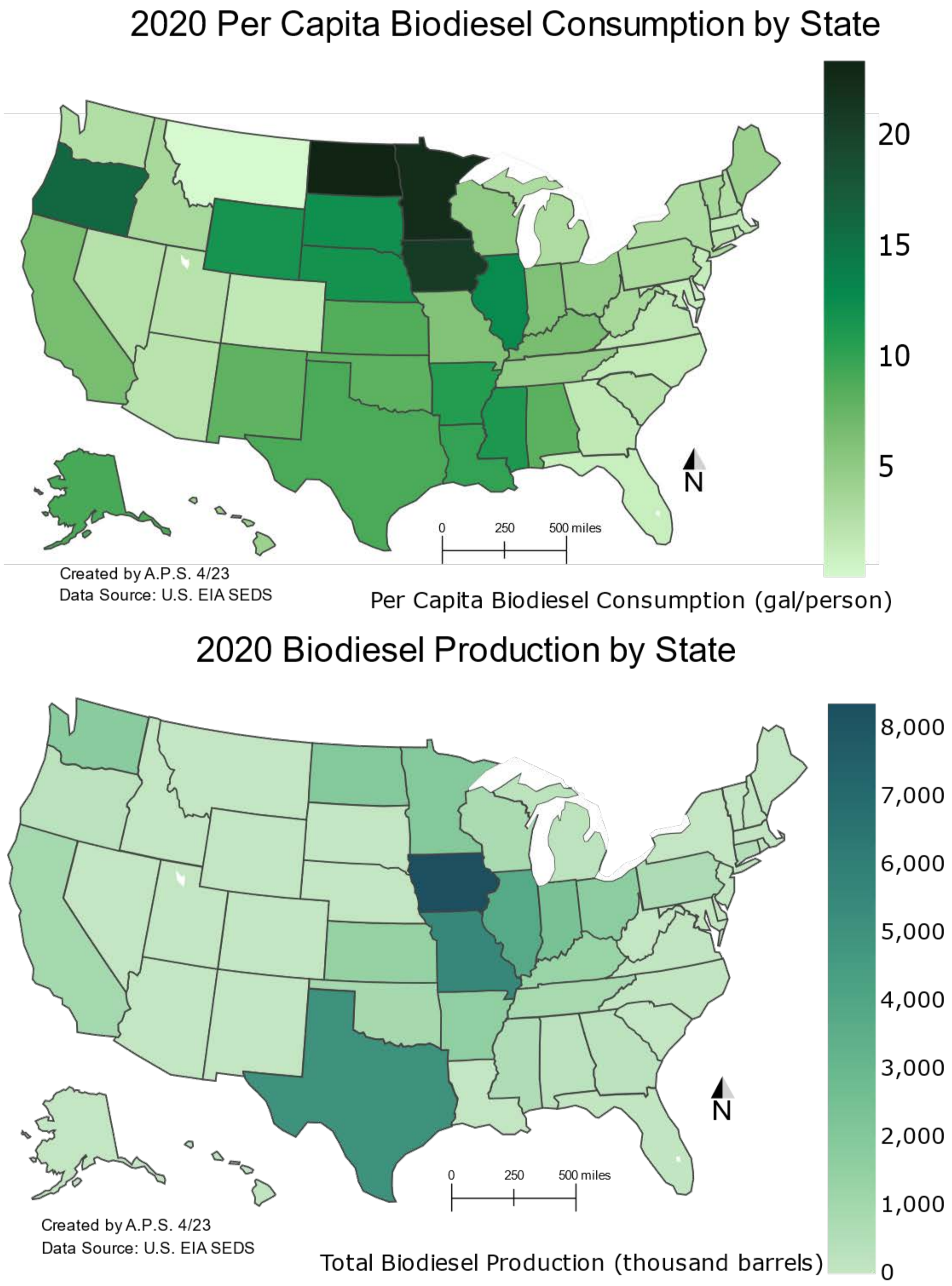

Figure S1.2. 2020 per capita biodiesel consumption by state in gallons per person and 2020 total biodiesel production by state in thousand barrels. [1,2]

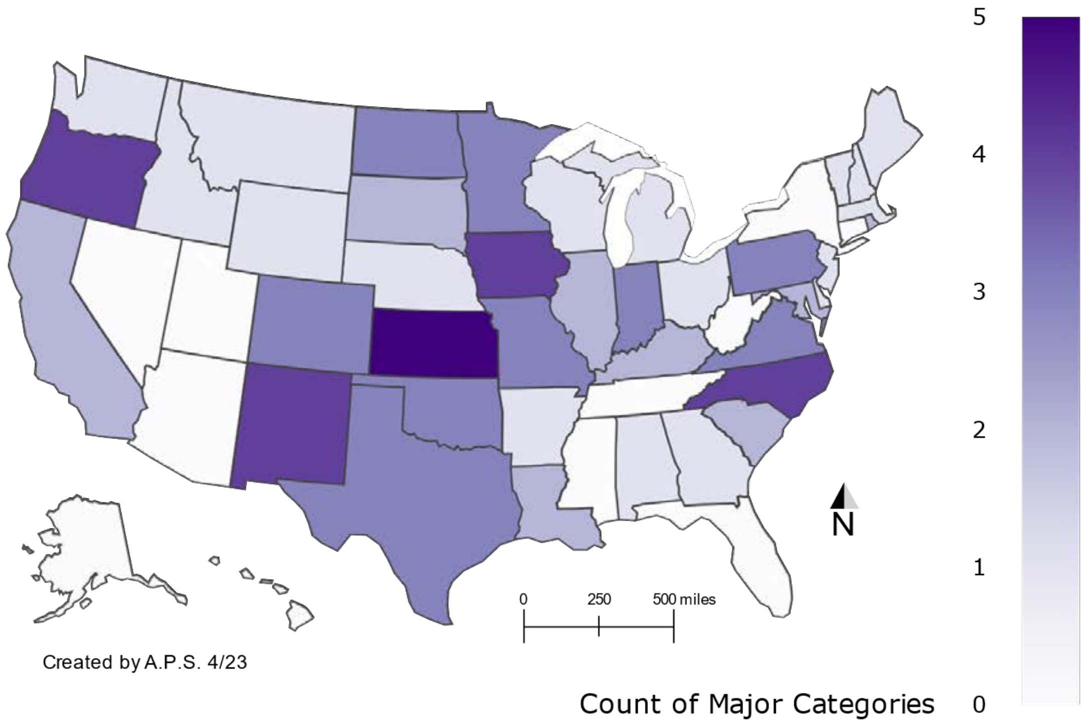

Figure S1.3. Number of major biodiesel-supporting policy categories addressed by state.

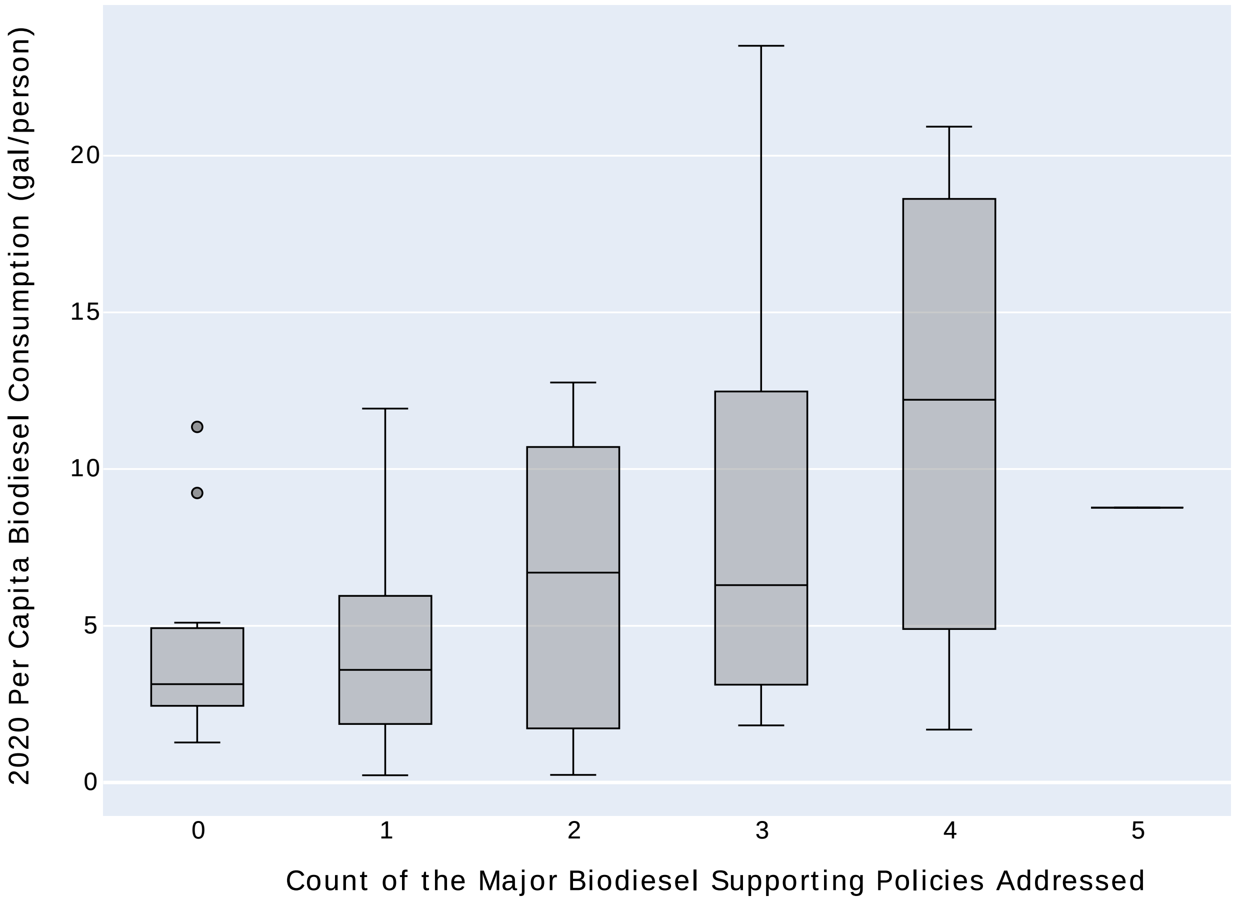

Figure S1.4. Biodiesel-supporting policy subcategories addressed and state-level per capita biodiesel consumption

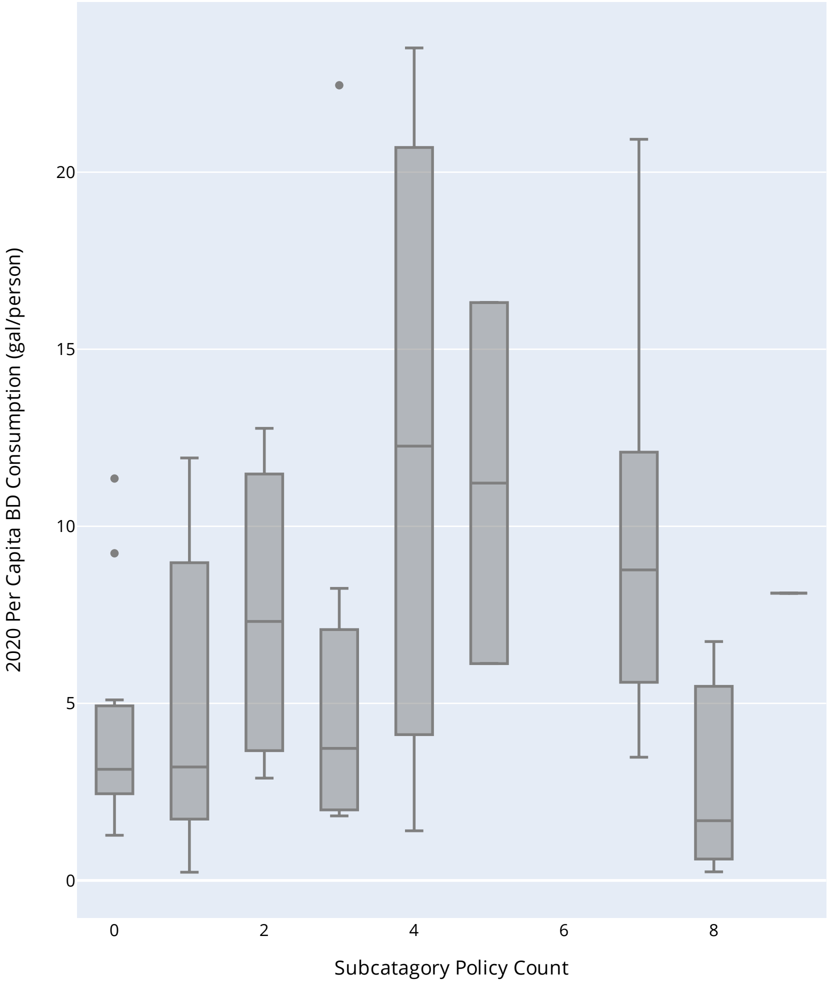

Figure S1.5. Biodiesel-supporting policy subcategories addressed and state-level per capita biodiesel consumption

REFERENCES

(1) U.S. EIA. *State Energy Consumption Estimates 1960 Through 2020*; DOE/EIA-0214(2020); 2022. https://www.eia.gov/state/seds/archive/seds2020.pdf.
(2) U.S. EIA. *State Energy Production Estimates 1960 Through 2020*; DOE/EIA-0214(2020); 2022.

Supporting Information 2: Driving Sustainability in the United States: Efficacy of State-Level Biodiesel Policy Approaches

AUTHOR NAMES: Adam P. Sibal, Ashlynn S. Stillwell*

AUTHOR ADDRESS: Civil and Environmental Engineering, University of Illinois Urbana-Champaign, 205 N Mathews Ave, MC-250, Urbana IL 61801, adamps2@illinois.edu

Table S2.1. U.S. Biodiesel Fuel Use Mandate Policies Summary

**State**	**Biodiesel Volume Mandate Minimum Requirement**	**Note**	**Applicability**	**Reference**
Arkansas	2%	State-owned or leased diesel-powered vehicles	Government vehicles	Arkansas Code 15-13-101, 15-13-102, and 15-13-202 to 15-13-203
California	LCFS	The California Air Resources Board regulations require transportation fuel producers and importers to meet specified average carbon intensity requirements for fuel. LCFS-regulated fuels include natural gas, electricity, hydrogen, and gasoline mixed with at least 10% corn-derived ethanol, biomass-based diesel, and propane. The LCFS Program allows producers and importers to generate, acquire, transfer, bank, borrow, and trade credits.	State-wide	California Code of Regulations Title 17, Section 95480-95490; and California Health and Safety Code 38500-38599
Illinois	5%	Applies to any diesel-powered vehicle owned or operated by the state, county, or local government, school district, community college, public college or university, or mass transit agency	Government vehicles	20 Illinois Compiled Statutes 689/10 and 625 Illinois Compiled Statutes 5/12-705.1
Indiana	2%	Applies to governmental entities and state educational institutions	Government vehicles	Indiana Code 5-22-5-8, and 21-31-9-3
Kansas	2%	State-owned diesel-powered vehicles and equipment must use a biodiesel blend that contains at least 2% biodiesel (B2), where available, as long as the price of the biodiesel blend is not more than $0.10 per gallon as compared to the price of diesel fuel.	Government vehicles and equipment	Kansas Statutes 75-3744a

State	Biodiesel Volume Mandate Minimum Requirement	Note	Applicability	Reference
Louisiana	2%		State-wide	LA Rev Stat § 3:3:4674, LA Rev Stat § 3:3:4674.1, and LA Rev Stat § 3:3:3712
Massachusetts	15%<br><br>5%*	State Agency Vehicles Use Only. On- and off-road<br><br>*All agencies that purchase and store diesel fuel at their facilities as of July 1, 2021, must purchase and use a biodiesel blend that contains at least 5% biodiesel (B5) in any diesel-powered vehicle owned or operated by the state	Government vehicles	Massachusetts Executive Office of Administration and Finance Administrative Bulletin 13 (2006)<br><br>*Massachusetts Executive Order 594 (2021)
Maryland	5%	At least 50% of state vehicles using petroleum diesel fuel must use a minimum blend of 5% biodiesel (B5) or other biofuel approved by the U.S. Environmental Protection Agency as a fuel or fuel additive. This requirement does not apply to any state vehicles for which the use of biodiesel or other biofuel will void the manufacturer's warranty for that vehicle	Government vehicles	State Finance and Procurement Code 14-408
Minnesota	20%*<br>5%**	*Between April and September<br>**The rest of the year	State-wide	Minnesota Statutes 239.75 and 239.77
Missouri	20%	Applies to 75% of the Missouri Department of Transportation fleet	Government vehicles	Missouri Revised Statutes 414.365 and 414.407
New Hampshire	5%	Applies to the department of transportation vehicles. B20 is encouraged	Government vehicles	New Hampshire Revised Statutes 228:24-a
New Jersey	Undefined	New Jersey state departments, agencies, offices, universities, and colleges must	Government vehicles	New Jersey Statutes 52:34-6.6 through 52:34-6.8

State	Biodiesel Volume Mandate Minimum Requirement	Note	Applicability	Reference
		purchase biofuels for use in motor vehicles		
New Mexico	5%	State-wide. Policy suspended from October 12, 2022, to April 15. 2023	State-wide	NM Stat § 57-19-27, NM Stat § 57-19-28 , NM Stat § 57-19-29
North Carolina	20%	Every school bus capable of operating on diesel fuel	School buses	North Carolina General Statutes 115C-240(c) and 115C-249(a)
Oregon	5%		State-wide	Oregon Revised Statutes 646.913 through 646.923 and Oregon Administrative Rules 603-027-0410 and 603-027-0420
Pennsylvania	2%*<br>5%**<br>10%***<br>20%****	*one year after in-state production of biodiesel reaches 40 million gallons<br>**one year after in-state production of biodiesel reaches 100 million gallons<br>***one year after in-state production of biodiesel reaches 200 million gallons<br>****one year after in-state production of biodiesel reaches 400 million gallons	State-wide	Title 74 Pennsylvania Statutes, Chapter 18H, Sections 1650.3, 1650.4, and 1650.4.1
South Carolina	5%	All state-owned diesel fueling facilities	Government Vehicles	South Carolina Code of Laws 12-63-30
Washington	2%<br>5%*<br>20%**	* 80 days after the Washington State Department of Agriculture (WSDA) determines that in-state feedstocks and oil-seed crushing capacity can meet a 3% requirement<br>**For state agency vehicles, vessels, and construction equipment	State-wide<br><br>Government vehicles, vessels, and equipment	Revised Code of Washington 19.112.010 and 19.112.110 -19.112.180<br><br>Revised Code of Washington 43.19.642 and 43.19.646
West Virginia	Undefined	West Virginia higher education governing boards must use alternative fuels to the maximum extent feasible.	Government vehicles	West Virginia Code 18B-5-9

Table S2.2. U.S. Biodiesel Fuel Use Incentive Policies Summary

State	Biodiesel Fuel Tax Incentives or Rebates	Applicability	Incentive or Rebate Recipient	Reference
Delaware	Taxes imposed on alternative fuels are waived	Government vehicles	Consumer	Delaware Code Title 30, Chapter 51, Subchapter II
Idaho	The state excise tax does not apply to special fuels when used in state or federal government-owned vehicles fuel suitable for use in diesel engines.	Government vehicles	Government	Idaho Statutes 63-2401, 63-2402, and 63-2423
Illinois	A sales and use tax of 6.25% applies to 100% of the proceeds from the sale of fuel blends containing between 1% and 10% biodiesel. Sales and use taxes do not apply to the proceeds from the sale of biodiesel blends containing between 11% and 99%	State-wide	Consumer	35 Illinois Compiled Statues 120/2-10, 105/3-10, and 105/3-44
Indiana	The special fuel tax does not apply to nominal biodiesel blends of at least 20% (B20); special fuel used only for a personal, noncommercial use and not for resale; and biodiesel used by a biodiesel producer holding an exemption certificate<br><br>The sale of biodiesel and blended biodiesel used to power an internal combustion engine or motor	State-wide	Consumer	Indiana Code 6-6-2.5 and 6-6-1.6<br><br>Indiana Code 6-2.5-5-51 and 6-6-2.5-22

State	Biodiesel Fuel Tax Incentives or Rebates	Applicability	Incentive or Rebate Recipient	Reference
	is exempt from state gross retail tax.			
Iowa	Retailers selling biodiesel blends containing a minimum of 5% biodiesel (B5) are eligible for a state income tax credit of $0.035 per gallon of biodiesel sold. Biodiesel blends containing a minimum of 11% biodiesel (B11) are eligible for a state income tax of $0.055 per gallon sold.	State-wide	Retailer	Iowa Code 422.11P
Kansas	A qualified motor fuel dealer is eligible for up to $0.03 for every gallon of biodiesel sold if the required threshold percentage is met. For biodiesel, the threshold increases incrementally on an annual basis from 2% in 2009 to 25% in 2025.<br><br>Kansas residents are eligible for a rebate from the Kansas Soybean Commission of up to $2,000 for fleets or $200 for individuals, for using biodiesel blends above 10% and 5% in diesel-powered vehicles, respectively. Fleets may be eligible for an additional rebate of $1 per	State-wide	Retailer<br><br>Consumer	Kansas Statutes 79-34,171 through 79-34,176<br><br>[1]

State	Biodiesel Fuel Tax Incentives or Rebates	Applicability	Incentive or Rebate Recipient	Reference
	gallon, up to $2,000, for annual biodiesel purchases.			
Kentucky	Qualified biodiesel producers or blenders are eligible for an income tax credit of $1.00 per gallon of pure biodiesel (B100) or renewable diesel produced or used in the blending process. Re-blending of blended biodiesel does not qualify for the tax credit. The total amount of credits claimed by all biodiesel producers may not exceed the annual biodiesel tax credit cap of $10 million. Unused credits may not be carried forward.	Statewide	Blender	Kentucky Revised Statutes 141.422 to 141.424
Maine	Biodiesel blends of 90-100% have a tax rate per gallon of $0.287 while diesel fuel has a tax rate of $0.312.	State-wide	Consumer	Maine Revised Statutes Title 36, Section 3203
Missouri	A retail service station or a distributor that sells a biodiesel blend directly to users is eligible for a state tax credit. Biodiesel blends containing a minimum of 5% biodiesel (B5) are eligible for a tax credit of $0.02 per gallon. Biodiesel blends containing a	State-wide	Retailer or distributor	Missouri House Bill 3, 2022, and Missouri Revised Statutes 135.755

State	Biodiesel Fuel Tax Incentives or Rebates	Applicability	Incentive or Rebate Recipient	Reference
	minimum of 11% biodiesel (B11) are eligible for a tax credit of $0.05 per gallon.			
Montana	A licensed distributor who pays the special fuel tax on biodiesel may claim a refund equal to $0.02 per gallon of biodiesel sold during the previous quarter if the biodiesel is made entirely from components produced in Montana. Additionally, the owner or operator of a retail motor fuel outlet may claim a refund equal to $0.01 per gallon of biodiesel purchased from a licensed distributor if the biodiesel is made entirely from components produced in the state.	State-wide	Distributor	Montana Code Annotated 15-70-433
New Mexico	Alternative fuel distributed by or used for the federal government, state government, or Indian nation, tribe, or pueblo purposes is exempt from the state excise tax.<br><br>Entities and individuals that receive or manufacture and deliver biodiesel within the state for blending or resale	Government, Indian nation, tribal or pueblo<br><br>Statewide	Government, Indian nation, tribal or pueblo<br><br>Consumer, Blender, Distributor, Retailer	New Mexico Statutes 7-16B-5<br><br>New Mexico Statutes 7-16A-10

State	Biodiesel Fuel Tax Incentives or Rebates	Applicability	Incentive or Rebate Recipient	Reference
	are eligible for a tax deduction for the fuel.			
North Carolina	The retail sale, use, storage, and consumption of alternative fuels are exempt from the state retail sales and use tax<br><br>Fuel blenders or suppliers of ethanol or biodiesel are not required to file a bond with the North Carolina Department of Revenue when the expected motor fuel tax liability is less than $2,000.	State-wide	Retailer<br><br>Blender and Distributor	North Carolina General Statutes 105-164.13 and 105-449.130<br><br>North Carolina General Statutes 105-449.60 and 105-449.72
North Dakota	A licensed fuel supplier who blends biodiesel or renewable diesel with diesel fuel may claim an income tax credit of $0.05 per gallon for fuel containing at least 5% biodiesel or renewable diesel.	State-wide	Blender	North Dakota Century Code 57-38-01.22
Rhode Island	Biodiesel is exempt from the $0.34 per gallon state motor fuel tax. Biodiesel may be blended with other fuel for use in motor vehicles, but only the biodiesel portion of the blended fuel is exempt	State-wide	Consumer	Rhode Island General Laws 31-36-1(6)

State	Biodiesel Fuel Tax Incentives or Rebates	Applicability	Incentive or Rebate Recipient	Reference
South Dakota	Biodiesel and biodiesel blends are taxed at the state motor fuel excise tax rate of $0.28 per gallon. Beginning the fiscal quarter after which a biodiesel production facility in the state reaches a nameplate capacity of at least 20 million gallons per year and fully produces at least 10 million gallons of biodiesel within one year, the tax on biodiesel and biodiesel blends is reduced to $0.26 per gallon.<br><br>Licensed biodiesel blenders are eligible for a tax credit for special fuel, including diesel that is blended with biodiesel. The tax credit is granted on a per-gallon basis in the amount that the rate for special fuel exceeds the rate for the biodiesel blend.	State-wide	Consumer<br><br>Blender	South Dakota Statutes 10-47B-3 through 10-47B-10<br><br>South Dakota Statutes 10-47B-121.1
Texas	The biodiesel portion of blended fuel containing taxable diesel is exempt from the diesel fuel tax.	State-wide	Consumer	Texas Statutes, Tax Code 162.204

State	Biodiesel Fuel Tax Incentives or Rebates	Applicability	Incentive or Rebate Recipient	Reference
Virginia	Alternative fuel is exempt from taxes if it is sold to a government entity for its exclusive use, a non-profit charitable organization for the purpose of providing charitable services for low-income medical patients, or produced by an agricultural operation and used exclusively for farm use or vehicles of that producer.	Government vehicles, non-profits, agriculture	Government, some consumers	Virginia Code 58.1-2250

Table S2.3 U.S. Biodiesel Production Incentive Policies Summary

State	Biodiesel Production Incentives Policy Summary	Reference
Alabama	A tax credit of 3% of the previous year's annual employee wages for up to 10 years.<br><br>A tax credit of 1.5% of qualified capital investment annually for up to 10 years.	Code of Alabama 2-2-90 and 40-18-370 through 40-18-383
Colorado	Grant offerings that can be applied to biofuel industries.	[2]
Georgia	A business that manufactures biofuels may claim an annual tax credit for five years. The amount is based on the number of eligible new full-time employee jobs and county.<br><br>The sale of personal property to an alternative fuel facility for the production and processing of biodiesel is exempt from the state sales and use tax.	Georgia Code 48-7-40<br><br>Georgia Code 48-8-3
Iowa	State tax incentives to business projects for the production of biomass or alternative fuels. Incentives may include an investment tax credit equal to a percentage of the qualifying investment, amortized over five years; a refund of state sales, service, or use taxes paid to contractors or subcontractors during construction; an increase of the state's refundable research activities credit; and a local property tax exemption of up to 100% of the value added to the property.	Iowa Code 15.335 and 422.10
Kansas	Qualified equipment used for storing and blending petroleum-based fuel with biodiesel is exempt from state property taxes. The exemption begins at the time of installation at a fuel terminal, refinery, or biofuel production plant, and ends 10 taxable years following the year of installation.	Kansas Statutes 79-232 and 79-32,251
Kentucky	Qualified biodiesel producers or blenders are eligible for an income tax credit of $1.00 per gallon of pure biodiesel (B100) or renewable diesel produced or used in the blending process. Re-blending of blended biodiesel does not qualify for the tax credit. The total amount of credits claimed by all biodiesel producers may not exceed the annual biodiesel tax credit cap of $10 million. Unused credits may not be carried forward.	Kentucky Revised Statutes 141.422 to 141.424

State	Biodiesel Production Incentives Policy Summary	Reference
Louisiana	Corporate or income tax credit for qualified capital infrastructure projects in Louisiana that are directly related to biofuel industries. The tax credit is for 7% to 18% of the project costs, calculated based on investment costs, up to $1,000,000 per state-certified green project. The portion of the base investment expended on payroll for Louisiana residents employed in connection with the construction of the project may be eligible for an additional 7.2% tax credit on the payroll.	Louisiana Revised Statutes 47:6037
Michigan	Industrial property that is used for the creation or synthesis of biodiesel fuel may be eligible for a tax exemption.	Michigan Compiled Laws 207.552 and 207.803 through 207.809
Minnesota	Offer grants, loans, or other financial incentives to alternative fuel retailers for the installation of ethanol blender pumps or other rural economic infrastructure activities, or to producers of transportation fuels from cellulosic material or bio-based products.	Minnesota Statutes 41A.12
Montana	Alternative fuel production facilities, including biodiesel, may qualify for a reduced property tax rate of 3% of market value. In addition, temporary property tax rate abatements are available for qualified biodiesel production facilities. The tax abatements are available during facility construction and for the first 15 years after the facility begins operation. The total time of the qualifying period may not exceed 19 years.<br><br>Biodiesel producers that produce biodiesel from waste vegetable oil feedstock are exempt from the state's special fuel tax. Waste vegetable oil is used as cooking oil gathered from restaurants or commercial food processors.	Montana Code Annotated 15-6-157, 15-6-158, and 15-24-3111<br><br>Montana Code Annotated 15-70-401 and 15-70-405
Nebraska	Motor fuels sold to an ethanol or biodiesel production facility and motor fuels manufactured at and sold from an ethanol or biodiesel facility are exempt from certain motor fuel tax laws.	Nebraska Revised Statutes 66-489 and 66-496
New Mexico	A tax credit is available for up to 30% of the cost of both purchasing and installing equipment used to produce biodiesel blends containing at least 2% biodiesel (B2). The tax credit is limited to $50,000 per facility and is claimed against gross receipts tax or compensating tax.<br><br>Entities and individuals that receive or manufacture and deliver biodiesel within the state for blending or resale are eligible for a tax deduction for the fuel.	New Mexico Statutes 7-9-79.2<br><br>New Mexico Statutes 7-16A-10

**State**	**Biodiesel Production Incentives Policy Summary**	**Reference**
North Dakota	Provides an interest buy-down of up to 5% below the note rate to biodiesel, ethanol, or renewable diesel production facilities; livestock operations feeding by-products produced at a biodiesel, ethanol, or renewable diesel facility; and grain handling facilities that provide storage of grain used in biofuels production. Qualified biodiesel, ethanol, and renewable diesel production facilities located in North Dakota may receive up to $500,000 of interest buydown for the purchase, construction, or expansion of a production facility, or the purchase or installation of equipment at the facility.<br><br>Qualified producers or blenders may be eligible for a corporate income tax credit of 10% of the direct costs incurred to add equipment to retrofit an existing facility or construct a new facility in the state to produce or blend diesel fuel containing at least 2% biodiesel or renewable diesel. A taxpayer may only claim the credit for up to five years and is limited to $250,000 in cumulative credits for all taxable years.<br><br>Offers loan guarantees of up to $400,000 per borrower for eligible entities constructing facilities using biomass for agriculturally-derived fuel production. The total value of loan guarantees under this program may not exceed $8 million at any one time. Additional restrictions apply.<br><br>Provides grants to enhance the production of low-emission technology projects. Eligible projects related to biofuel, hydrogen, natural gas, and energy efficiency. CSEA may award up to $25 million between July 1, 2021, and June 30, 2023. Eligible applicants include corporations, cooperatives, associations, and others.	North Dakota Century Code 17-03<br><br>North Dakota Century Code 57-38-30.6<br><br>North Dakota Century Code 6-09.7-01 and 6-09.7-09<br><br>North Dakota House Bill 1452, 2021, and North Dakota Century Code 17-01 and 17-07
Oklahoma	Provides technical and regulatory assistance to small businesses that need permits to construct and operate a biodiesel production facility.	[3]
Oregon	Offers low-interest loans for fuel production facilities and dedicated feedstock production.<br><br>Property used to produce biofuels, including ethanol and biodiesel, may be eligible for a property tax exemption if it is located in a designated Rural Renewable Energy Development Zone.	Oregon Revised Statutes 470<br><br>Oregon Revised Statutes 285C.350 through 285C.370

State	Biodiesel Production Incentives Policy Summary	Reference
South Dakota	A licensed biodiesel producer may apply for and obtain a tax refund for state fuel taxes paid on methanol used to produce biodiesel.	South Dakota Statutes 10-47B-121.1
Virginia	Qualified biodiesel and green diesel producers are eligible for a tax credit of $0.01 per gallon of biodiesel or renewable diesel fuels produced. This credit is available for producers who generate up to two million gallons of biodiesel or renewable diesel fuel per year. The annual credit may not exceed $5,000, and producers are only eligible for the credit for the first three years of production.<br><br>Qualified employers are eligible for a $500 tax credit for each new green job created that offers a salary of at least $50,000, for up to 350 jobs per employer. The credit is allowed for the first five years that the job is continuously filled. For the purposes of this tax credit, a green job is defined as employment in industries including biofuels.<br><br>Provides grants to promote and develop the agriculture and forestry industry in Virginia and create or expand value-added facilities, including qualified biofuel production facilities. Individual grants may not exceed $500,000 or 25% of qualified capital expenditures.<br><br>Offers up to $10,000 to state agencies and local governments for the incremental cost of new or converted AFVs.	Virginia Code 58.1-439.12:02<br><br>Virginia Code 58.1-439.12:05<br><br>Virginia Code 3.2-304<br><br>[4]
Washington	Waste vegetable oil, specifically cooking oil gathered from restaurants or commercial food processors, used by an individual to produce biodiesel for personal use are exempt from state sales and use taxes.	Revised Code of Washington 82.08.0205 and 82.12.0205
Wyoming	Alternative fuel sold for use in motor vehicles and intended for export from the state by a licensed alternative fuel exporter is exempt from the alternative fuel license tax. Any person exporting alternative fuel for which the license tax has been paid is eligible for a refund of the license tax paid.	Wyoming Statutes 39-17-301, 39-17-305, and 39-17-309©

Table S2.4 U.S. Biodiesel Fueling Infrastructure Incentive Policies Summary

State	Biodiesel Blending, Distribution, and Infrastructure Incentives	Applicability	Reference
California	Provide financial incentives for businesses, vehicle and technology manufacturers, workforce training partners, fleet owners, consumers, and academic institutions with the goal of developing and deploying alternative and renewable fuels and advanced transportation technologies including biofuels.	Statewide	California Health and Safety Code 44272 - 44273 and California Code of Regulations, Title 13, Chapter 8.1
Colorado	Funding for the incremental cost of alternative fuel vehicles and alternative fueling infrastructure for public fleets.	Government	[5]
Iowa	Biodiesel distributors may apply for cost-share grants for infrastructure upgrades and installations at biodiesel terminal facilities. Facilities blending or dispensing blends ranging from 2% biodiesel (B2) to 98% biodiesel (B98) are eligible for up to 50% of the total project, up to $50,000. Facilities blending or dispensing B99 or B100 are eligible for up to 50% of the total project, up to $100,000.	Corporations	Iowa Code 159A.13-159A.15
Kansas	An income tax credit is available for 40% of the total cost to install alternative fueling infrastructure. Qualified property must be directly related to the delivery of alternative fuel into the fuel tank of an alternative fuel vehicle. The tax credit may not exceed $100,000 per fueling station. The credit is only available to entities with corporate income tax liability.<br><br>Qualified equipment used for storing and blending petroleum-based fuel with biodiesel is exempt from state property taxes. The exemption begins at the time of installation at a fuel terminal, refinery, or biofuel production plant, and ends 10 taxable years following the year of installation.	Corporations<br><br>Corporations	Kansas Statutes 79-32,201<br><br>Kansas Statutes 79-232 and 79-32,251
Minnesota	Offer grants, loans, or other financial incentives to alternative fuel retailers for the installation of ethanol blender pumps or other rural economic infrastructure activities, or to producers of transportation fuels from cellulosic material or bio-based products.	Corporations	Minnesota Statutes 41A.12
Missouri	Offers business grants for the development, construction, installation, upgrade, or retrofit of biofuel infrastructure. Biodiesel blends must be 6% biodiesel (B6) or higher. Terminal companies, fuel distributors, or fuel retailers are allotted 75% of the covering the lesser of 50% of costs or $500,000. Any fuel retailer with five or less stations, fleet operations, or individual businesses are allotted 25% of the available funds covering the lesser of 75% of the costs or $250,000	Corporations	[6]

State	Biodiesel Blending, Distribution, and Infrastructure Incentives	Applicability	Reference
New Mexico	In calculating the annual petroleum products loading fee, a biodiesel blending facility owner may deduct the number of biodiesel gallons delivered to be blended into petroleum products.<br><br>Entities and individuals that receive or manufacture and deliver biodiesel within the state for blending or resale are eligible for a tax deduction for the fuel.	Corporations	New Mexico Statutes 7-13A-5<br><br>New Mexico Statutes 7-16A-10
North Carolina	Alternative Fuel Revolving Fund (Fund) for state agencies to offset the incremental costs of purchasing biodiesel blends of at least 20% (B20) and developing alternative fueling infrastructure. For the purposes of this program, alternative fuels include 100% biodiesel (B100) and biodiesel blends of at least B20.	Government	North Carolina General Statutes 143-58.4, 143-58.5, 143-341, and 136-28.13
North Dakota	Qualified retailers may be eligible for a corporate income tax credit of 10% of the direct costs incurred to adapt or add equipment to a facility so that it may sell diesel fuel containing at least 2% biodiesel or renewable diesel. A retailer may only claim the credit for up to five years and is limited to $50,000 in cumulative credits for all taxable years.<br><br>Qualified producers or blenders may be eligible for a corporate income tax credit of 10% of the direct costs incurred to add equipment to retrofit an existing facility or construct a new facility in the state for the purpose of producing or blending diesel fuel containing at least 2% biodiesel or renewable diesel. A taxpayer may only claim the credit for up to five years and is limited to $250,000 in cumulative credits for all taxable years.	Corporations<br><br>Corporations	North Dakota Century Code 57-38-01.23
Oklahoma	Issues grants for alternative fuel and advanced technology vehicle projects in the Oklahoma City Area Regional Transportation Study (OCARTS) area. Projects must provide a reduction in vehicle equipment emissions and cannot increase the number of vehicles in applicant fleets. Eligible projects may also include AFV fueling stations.	Corporations	[7]
Oregon	Offers low-interest loans for fueling infrastructure, and fleet vehicles.	Corporations	Oregon Revised Statutes 470

State	Biodiesel Blending, Distribution, and Infrastructure Incentives	Applicability	Reference
Pennsylvania	Provides financial assistance for innovative, advanced fuel and vehicle technology projects including:<br>-Incremental cost expenses relative to retrofitting vehicles to operate on alternative fuels;<br>-Incremental cost expenses to purchase alternative fuel vehicles;<br>-The cost to purchase and install the necessary fleet- or home-refueling equipment for alternative fuel vehicles; or,<br>-The cost to perform research, training, development, and demonstration of new applications or next-phase technology related to alternative fuel vehicles..	Corporations, government, and non-profits	Title 73 Pennsylvania Statutes, Chapter 18E, Section 1647.3
Texas	Provides grants for various types of clean air projects to improve air quality in the state's nonattainment areas and other affected counties. Eligible projects include those that involve replacement, retrofit, repower, or lease or purchase of new heavy-duty vehicles; alternative fuel dispensing infrastructure; idle reduction and electrification infrastructure; and alternative fuel use. The Rebate Grants Program provides grants to upgrade or replace diesel heavy-duty vehicles and non-road equipment. Qualifying projects must reduce emissions of nitrogen oxides or other pollutants by at least 25% as compared to baseline levels and must meet operational and fuel usage requirements.	Corporations, government, and non-profits	Texas Statutes Health and Safety Code 386 and Texas Administrative Code 114.620-114.629
Virginia	Qualified employers are eligible for a $500 tax credit for each new green job created that offers a salary of at least $50,000, for up to 350 jobs per employer. The credit is allowed for the first five years that the job is continuously filled. For the purposes of this tax credit, a green job is defined as employment in industries including biofuels.	Corporations	Virginia Code 58.1-439.12:05
Washington, D.C.	A tax credit is also available for 50% of the equipment and labor costs for the purchase and installation of alternative fuel infrastructure.	District-wide	District of Columbia Code 47-1806.12 through 47-1806.13, 47-1807.10 through 47-1807.11, and 47-1808.10 through 47-1808.11

Table S2.5 Biodiesel Fleet Development Incentive Policies

State	Vehicle Deployment Infrastructure Incentives	Applicability	Reference
California	Fund alternative mobile source emissions and trip reduction programs, including alternative fuel vehicle projects, on an ongoing basis. Programs such as low-emission, alternative fuel, or zero-emission vehicle procurement and old vehicle scrapping may be considered for funding.<br><br>The South Coast Air Quality Management District (SCAQMD) requires government fleets and private contractors under contract with public entities to purchase non-diesel lower emission and alternative fuel vehicles. The rule applies to transit buses, school buses, refuse haulers, and other vehicle fleets of at least 15 vehicles that operate in Los Angeles, San Bernardino, Riverside, and Orange counties.<br><br>The fleet rule for public agencies and utilities requires fleets to install VDECS on vehicles or purchase vehicles that run on alternative fuels or use advanced technologies to achieve emissions requirements by specified implementation dates.<br><br>Provide financial incentives for businesses, vehicle and technology manufacturers, workforce training partners, fleet owners, consumers, and academic institutions with the goal of developing and deploying alternative and renewable fuels and advanced transportation technologies including biofuels.	Corporations<br><br>Government<br><br>Government<br><br>State-wide	[8]<br><br>SCAQMD Rules 1186.1 and 1191-1196<br><br>California Code of Regulations Title 13, 2021-2027<br><br>California Health and Safety Code 44272 - 44273 and California Code of Regulations, Title 13, Chapter 8.1

State	Vehicle Deployment Infrastructure Incentives	Applicability	Reference
Colorado	Funding for the incremental cost of alternative fuel vehicles and alternative fueling infrastructure for public fleets.	Government	[5]
Idaho	Rebates for the replacement of qualified medium- and heavy-duty diesel vehicles with new diesel or alternative fuel vehicles.	State-Wide	[9]
Illinois	Reimburse any qualifying school district for the cost of converting gasoline buses to more fuel-efficient engines or engines using alternative fuels.	Government	105 Illinois Compiled Statutes 5/29-5
Indiana	Grants ranging from $50,000 to $1,000,000 for replacing or converting a diesel vehicle or vehicle component with one that operates on alternative fuel.	State-Wide	[10]
Iowa	The Iowa Department of Transportation (IowaDOT) provides funding for the replacement, retrofit, or conversion of medium- and heavy-duty (MHD) on-road diesel vehicles with new diesel or alternative fuel vehicles. Grants are also available for off-road diesel vehicle replacements and repowers. Grants are available for MHD school buses, transit buses, and trucks. Non-road vehicles and equipment may also be eligible for funding. Eligible applicants include government, nonprofit, and private entities that own or operate diesel fleets and equipment.	Corporations, Government, Non-Profit	[11]

State	Vehicle Deployment Infrastructure Incentives	Applicability	Reference
Kansas	An income tax credit is available for 40% of the incremental or conversion cost for qualified AFVs, based on gross vehicle weight rating. Up to $2,400 for less than 10,000 lbs, up to $4,000 for 10.000 - 26,000 lbs, and up to $40,000 for over 26,000 lbs. Alternatively, a tax credit of 5% of the cost of the AFV, up to $750, is available for the purchase of an original equipment manufacturer AFV. Qualified AFVs include vehicles that operate on a combustible liquid derived from grain starch, oil seed, animal fat, or other biomass, or produced from a biogas source. Only the first individual to take the title of the vehicle may receive this credit. The credit is only available to entities with corporate income tax liability.	Corporations	Kansas Statutes 79-32,201
Maryland	Provides grants to fleets for the retrofit or purchase of new AFVs. Grant award amounts vary and may cover up to 100% of the incremental AFV cost. For biodiesel, the maximum grant award per vehicle is $7,500 for Class 1-2 and $50,000 for Class 3-8 vehicles. Eligible applicants must be a fleet vehicle operator or purchaser and may include school districts, nonprofits, commercial entities, corporations, and local and municipal governments. AFVs purchased for individual or personal use are ineligible.	Corporations, Government, Non-Profit	[12]

State	Vehicle Deployment Infrastructure Incentives	Applicability	Reference
North Carolina	Provides grants to repower, replace, and convert eligible on- and off-road vehicles and equipment to alternative fuels and fuel-efficient technology.<br><br>Alternative Fuel Revolving Fund (Fund) for state agencies to offset the incremental costs of purchasing biodiesel blends of at least 20% (B20) and developing alternative fueling infrastructure. For the purposes of this program, alternative fuels include 100% biodiesel (B100) and biodiesel blends of at least B20.<br><br>Provide funding for heavy-duty on-road new diesel or alternative fuel vehicles or engine repowers and replacements, as well as off-road repowers and replacements. Both government and non-government entities that own and operate diesel fleets and equipment are eligible for funding. Applies to various types of vehicles and equipment.	State Wide<br><br>Government<br><br>Corporations	[13]<br><br>North Carolina General Statutes 143-58.4, 143-58.5, 143-341, and 136-28.13<br><br>[14]
Ohio	All newly acquired state agency vehicles must be capable of using an alternative fuel and must use the relevant alternative fuel if it is reasonably priced and available. Alternative fuel is defined as any fuel containing 85% or more ethanol (E85), fuel blends containing at least 20% biodiesel (B20).	Government	Ohio Revised Code 125.831-125.836
Oklahoma	Issues grants for alternative fuel and advanced technology vehicle projects in the Oklahoma City Area Regional Transportation Study (OCARTS) area. Projects must provide a reduction in vehicle equipment emissions and	Corporations	[7]

State	Vehicle Deployment Infrastructure Incentives	Applicability	Reference
	cannot increase the number of vehicles in applicant fleets. Eligible projects may also include AFV fueling station.		
Oregon	Offers low-interest loans for fueling infrastructure, and fleet vehicles.<br><br>For at least 25% of new state light-duty vehicles to the maximum extent possible, if ZEVs are not feasible, the state agency may purchase or lease AFVs and use alternative fuels to operate those vehicles, except in regions where it is not economically or logistically possible to fuel an AFV.<br><br>Award grants to owners and operators of at least 450 school buses powered by diesel engines. Eligible vehicles include buses that have at least three years of remaining useful life. Grants will be available for 30%, up to $50,000, for the purchase of a new bus or up to 100% of the cost to retrofit a school bus with emissions-reducing parts or technology that reduce diesel particulate matter emissions by at least 85%.	Corporations<br><br>Government<br><br>Government	Oregon Revised Statutes 470<br><br>Oregon Revised Statutes 283.327 and 267.030 and Executive Order 20-04, 2020<br><br>Oregon Revised Statutes 468A.795-468A.803
Pennsylvania	Provides financial assistance for innovative, advanced fuel and vehicle technology projects including:<br>-Incremental cost expenses relative to retrofitting vehicles to operate on alternative fuels;<br>-Incremental cost expenses to purchase alternative fuel vehicles;<br>-The cost to purchase and install the necessary fleet- or home-refueling equipment for	Corporations, government, and non-profits	Title 73 Pennsylvania Statutes, Chapter 18E, Section 1647.3

State	Vehicle Deployment Infrastructure Incentives	Applicability	Reference
	alternative fuel vehicles; or,<br>-The cost to perform research, training, development, and demonstration of new applications or next-phase technology related to alternative fuel vehicles.		
Rhode Island	At least 75% of state motor vehicle acquisitions must be AFVs.	Government	Rhode Island Executive Order 15-17
South Carolina	The South Carolina Department of Education must fuel state school bus fleets with biodiesel when feasible.<br><br>Provides low-interest loans for a variety of energy efficiency improvements, including AFV conversions and the incremental costs of a new AFV, with qualified project payback periods. Loans may cover up to 100% of project costs, ranging from $25,000 to $500,000 per state fiscal year. Eligible recipients include state agencies, local governments, public colleges and universities, school districts, and private non-profit organizations. State agencies and public educational institutions may combine their loan with a ConserFund Plus grant, which may cover up to 30% of total project costs.<br><br>Provides low-interest loans for a variety of energy efficiency improvements, including AFV conversions and incremental costs, with qualified project payback periods. Eligible recipients include businesses and industries. Utilities, non-profit organizations, and government entities may be eligible under special conditions. The loan may cover up to	Government<br><br>Corporations	South Carolina Code of Laws 48-52-650<br><br>South Carolina Code of Laws 48-52-650

State	Vehicle Deployment Infrastructure Incentives	Applicability	Reference
	100% of the project costs, ranging from $50,000 to $1 million.		
Texas	Provides grants for various types of clean air projects to improve air quality in the state's nonattainment areas and other affected counties. Eligible projects include those that involve replacement, retrofit, repower, or lease or purchase of new heavy-duty vehicles; alternative fuel dispensing infrastructure; idle reduction and electrification infrastructure; and alternative fuel use. The Rebate Grants Program provides grants to upgrade or replace diesel heavy-duty vehicles and non-road equipment. Qualifying projects must reduce emissions of nitrogen oxides or other pollutants by at least 25% as compared to baseline levels and must meet operational and fuel usage requirements.	Corporations and Government<br><br>Government<br><br>Government	Texas Statutes Health and Safety Code 386 and Texas Administrative Code 114.620-114.629<br><br>Texas Statutes, Government Code 2158.004-2158.009<br><br>Texas Statutes, Health and Safety Code 390, and Texas Administrative Code 114.640-114.648

State	Vehicle Deployment Infrastructure Incentives	Applicability	Reference
	State agency fleets with more than 15 vehicles, excluding emergency and law enforcement vehicles, may not purchase or lease a motor vehicle unless the vehicle uses natural gas, propane, ethanol, or fuel blends of at least 85% ethanol (E85), methanol or fuel blends of at least 85% methanol (M85), biodiesel or fuel blends of at least 20% biodiesel (B20), or electricity (including plug-in hybrid electric vehicles). Covered state agency fleets must consist of at least 50% of vehicles that are able to operate on alternative fuels and use these fuels at least 80% of the time the vehicles are driven. Covered state agencies may meet these requirements through the purchase of new vehicles or the conversion of existing vehicles.<br><br>Any public school district or charter school may receive a grant through the Texas Commission on Environmental Quality (TCEQ) to pay for the incremental costs to replace school buses or install diesel oxidation catalysts, diesel particulate filters, emission-reducing add-on equipment, and other emissions reduction technologies in qualified school buses.		
Utah	At least 50% of new or replacement light-duty state agency vehicles must meet Bin 2 emissions standards established in Title 40 of the U.S. Code of Federal Regulations, or be propelled to a significant extent by electricity, natural gas, propane, hydrogen, or biodiesel.	Government	Utah Code 63A-9-401 and 63A-9-403

State	Vehicle Deployment Infrastructure Incentives	Applicability	Reference
Vermont	Provides funding to local, state, and regional agencies or departments, businesses, institutions, and nonprofit organizations for projects focused on reducing emissions from diesel engines and vehicles. Qualifying heavy-duty vehicles include buses and Class 5-8 trucks. Projects include certified engine replacements; alternative fuel conversions; and certified vehicle or equipment replacements.	Corporations, government, and non-profits	[15]
Washington, D.C.	Businesses and individuals are eligible for an income tax credit of 50% of the equipment and labor costs for the conversion of qualified AFVs, up to $19,000 per vehicle.<br><br>The tax credit is also available for 50% of the equipment and labor costs for the purchase and installation of alternative fuel infrastructure.	District-wide	District of Columbia Code 47-1806.12 through 47-1806.13, 47-1807.10 through 47-1807.11, and 47-1808.10 through 47-1808.11
Wisconsin	Provide school districts financial aid to cover the incremental cost of purchasing biodiesel to operate school buses, as compared to the cost of petroleum diesel fuel.<br><br>Funding is available for the replacement and scrapping of model year 1992-2009 heavy-duty public transit buses with new replacement diesel or alternative fueled buses<br><br>Funding for 25% to 100% of eligible project costs is available to businesses, nonprofits, and public entities that reduce diesel emissions by replacing engines, retrofitting exhaust controls, purchasing new vehicles, or	Government<br><br>Government<br><br>Government, corporations, nonprofits	Wisconsin Statutes 121.575<br><br>[16]<br><br>[17]

State	Vehicle Deployment Infrastructure Incentives	Applicability	Reference
	installing idle reduction equipment. Eligible projects include school buses, transit buses, and non-road engines, equipment, or vehicles.		

REFERENCES

(1) Metropolitan Energy Center. *Biodiesel Rebate*. https://metroenergy.org/biodiesel-rebate/ (Date last accessed 2023-02-11).
(2) Colorado Office of Economic Development & International Trade. *Advanced Industries Accelerator Programs*. https://oedit.colorado.gov/advanced-industries-accelerator-programs (Date last accessed 2023-04-11).
(3) Oklahoma Department of Environmental Quality. *Business Assistance*. https://www.deq.ok.gov/external-affairs-division/for-business/business-assistance/ (Date last accessed 2023-02-11).
(4) Virginia Clean Cities. *CMAQ Vehicle Fuel Conversion Incentive Program*. https://vacleancities.org/reports-2/cmaq-incentive-program/ (Date last accessed 2023-02-11).
(5) Colorado Department of Local Affairs. *Energy/Mineral Impact Assistance Fund Grant (EIAF) | Department of Local Affairs*. https://cdola.colorado.gov/funding-programs/energy/mineral-impact-assistance-fund-grant-eiaf (Date last accessed 2023-02-11).
(6) Missouri Department of Agriculture. *Biofuel Infrastructure Incentive Program*. https://www.agriculture.mo.gov/abd/financial/biofuelgrant.php (Date last accessed 2023-02-11).
(7) Association of Central Oklahoma Governments. *Fleet Conversion Grants* . https://www.acogok.org/transportation-planning/air-quality/fleet-conversion-grants/#:~:text=The%20Association%20of%20Central%20Oklahoma%20Governments'%20CLEAN%20AIR%20Grants%20for,vehicles%20and%20alternative%20fuel%20vehicle (Date last accessed 2023-02-11).
(8) South Coast Air Quality Management District. *Air Quality Investment Program*. http://www.aqmd.gov/home/programs/business/business-detail?title=air-quality-investment-program (Date last accessed 2023-02-11).
(9) Department of Environmental Quality. *Volkswagen and Diesel Funding*. https://www.deq.idaho.gov/air-quality/improving-air-quality/volkswagen-and-diesel-funding/ (Date last accessed 2023-02-11).
(10) Indiana Department of Environmental Management. *DieselWise*. https://www.in.gov/idem/airquality/dieselwise/ (Date last accessed 2023-02-11).
(11) Iowa Department of Transportation. *Diesel Emissions Reduction Act in Iowa*. https://iowadot.gov/dera/ (Date last accessed 2023-02-11).
(12) Maryland Energy Administration. *Clean Fuels Incentive Program*. https://energy.maryland.gov/transportation/Pages/Clean-Fuels-Incentive-Program.aspx (Date last accessed 2023-02-11).
(13) North Carolina Department of Environmental Quality. *Mobile Sources Emissions Reductions Grant | NC DEQ*. https://www.deq.nc.gov/about/divisions/air-quality/motor-vehicles-and-air-quality/mobile-sources-emissions-reductions-grant (Date last accessed 2023-02-11).

(14) North Carolina Department of Environmental Quality. *Diesel Bus and Vehicle Program*. https://www.deq.nc.gov/about/divisions/air-quality/motor-vehicles-and-air-quality/volkswagen-settlement/phase-2-volkswagen-settlement/diesel-bus-and-vehicle-program (Date last accessed 2023-02-11).
(15) Vermont Department of Environmental Conservation. *Diesel Emissions Reduction Assistance*. https://dec.vermont.gov/air-quality/mobile-sources/diesel-emissions/vt-diesel-grant (Date last accessed 2023-02-11).
(16) Wisconsin Department of Administration. *VW Mitigation Program*. https://doa.wi.gov/Pages/vwsettlementwisconsin.aspx (Date last accessed 2023-02-11).
(17) (Wisconsin Department of Natural Resources. *Clean Diesel Grant Programs*. https://dnr.wisconsin.gov/Aid/CleanDiesel.html (Date last accessed 2023-02-11).

Supporting Information 3: Driving Sustainability in the United States: Efficacy of State-Level Biodiesel Policy Approaches

AUTHOR NAMES: Adam P. Sibal, Ashlynn S. Stillwell*

AUTHOR ADDRESS: Civil and Environmental Engineering, University of Illinois Urbana-Champaign, 205 N Mathews Ave, MC-250, Urbana IL 61801, adamps2@illinois.edu

Contents

S3.1. Background.......... S46
S3.2 Colorado Biodiesel Case Study Methodology Summary S47
S3.3 Colorado Biodiesel Case Study Results Summary S49
S3.4 Colorado Biodiesel Case Study Methodology Detailed Modeling Calculations Description S52
S3.5. Biodiesel Fuel Price Distributions.......... S57
S3.6. Additional Emissions Reduction Figures S58
S3.7. Additional Social Cost/Benefit Figures.......... S76
S3.7 Discussion on Potential of Biodiesel Production in Colorado.......... S81
S3.8 Sensitivity Analysis Results.......... S82

S3.1. Background

The state of Colorado is plagued by smog, Denver's notorious "brown cloud",[1] and ozone pollution. Additionally, abundant emissions of greenhouse gases (GHGs) and criteria pollutants such as carbon dioxide (CO_2), particulate matter (PM), carbon monoxide (CO), volatile organic compounds (VOCs), nitrous oxides (NO_X), sulfur oxides (SO_X), and methane (CH_4) contribute to global warming, acid rain, and adverse human health impacts.[2] In 2022, all or portions of 9 counties along Colorado's front range (Adams, Arapahoe, Denver, Boulder, Broomfield, Douglas, Jefferson, Larimer, and Weld counties) were downgraded by the U.S. Environmental Protection Agency (EPA) to Severe Nonattainment Areas for their ground level ozone pollution levels.[3] Between 2018 and 2020, these areas averaged 81 parts per billion (ppb) of ozone, well above the EPA 70 ppb threshold, making the area's ozone levels second only to New York City among major metropolitan areas.[4] This ruling will cost Colorado $43.4 million in estimated state funding to handle additional permitting workload, hire additional staff, and procurement and operation of additional air monitoring equipment.[5] These pollutants also contribute to significant deleterious human health impacts.[6] In 2019, the University of Washington's Institute for Health Metrics and Evaluation estimated that more than 800 annual deaths could be attributed to air pollution issues in the State of Colorado.[7]

Approximately 20% of the state's emissions come from the transport sector, growing to 25% by 2030.[8] Reports from the EPA demonstrate that most of the vehicle miles traveled (VMT) occur within nonattainment zones along the I-25 and I-70 corridors with fuel consumption contributing to the high levels of pollution.[3] The medium-duty (MD) and heavy-duty (HD) vehicle classes account for 25% of the total fuel use in Colorado. Diesel-consuming vehicles in the HD, MD, light-duty (LD), and bus sectors accounted for 6.6 billion VMT in Colorado in 2020.[9]

Consumption of biodiesel does incur tradeoffs, however. Based on 2017-2022 values, 20% biodiesel blended diesel fuel (B20) nationally sold for $0.12 ± $0.09 less per gallon than traditional fossil fuel diesel, even when accounting for the reduced energy content in biodiesel blends compared to fossil fuel diesel.[10] Colorado, within the Rocky Mountain Region, has a $0.28 ± $0.20 higher cost per gallon of fuel on an energy basis, largely due to the lack of biodiesel production or distribution infrastructure in the state.[10] Additionally, biodiesel and diesel fuels are not chemically identical. The lower heating value of pure diesel fuel is 128,700 British thermal units (BTU)/gallon (gal) and 117,100 BTU/gal for 100% biodiesel (B100), implying that 1.1 gallons of B100 must be consumed per 1 gallon of fossil diesel fuel to provide the same energy content, with values proportionally scaled down as the biodiesel blend volume decreases.[10] Due to the chemical differences in the fuels, blend volumes above B20 require modifications to engines, storage, and distribution systems to prevent gelling at low temperatures and damage to rubber components.[11] Blend volumes less than B20 can be readily blended and utilized in engines without voiding warranties, requiring additional maintenance or storage and dispensing issues.[12]

S3.2 Colorado Biodiesel Case Study Methodology Summary

We analyze how the use of varied biodiesel blends in Colorado's diesel fuel mix could affect CO_2, PM, CO, VOCs, NO_X, SO_X, and CH_4 emissions. We use a model of Colorado's diesel-powered fleet incorporating electrification projections coupled with data from the well-to-wheels full life-cycle analysis in the GREET model for vehicle classes in this fleet to understand the social costs and tradeoffs associated with biodiesel use. To better understand these tradeoffs, we model biodiesel blend volume in each year under uncertainty from 2024-2033 and quantify the social cost savings based on the higher cost to Colorado's citizens per gallon of biodiesel blended fuels compared with the social benefits of potential reductions in CO_2, CO, PM, VOCs, NO_X, SO_X, and CH_4 pollutants associated with use of biodiesel blends. Due to the primary concern in Colorado being air pollutant emissions, this analysis focuses on those emissions and does not include an assessment of impact to water demand and pollution or potential biodiversity loss. The model also does not account specifically for land use change outside of the emissions associated with land use changes included in GREET. Additionally, we present the social cost savings that Colorado could achieve if the biodiesel fuel price in Colorado could be aligned with the U.S. national average.

We determined the VMT of each vehicle class per year based on 2020 data and projected compound annual growth rates (CAGR) from the Colorado Medium and Heavy Duty (M/HD) Vehicle Study as shown in Table S3.1.[9]

To consider the electrification of the fleet, projections were mirrored with a uniformly distributed CAGR of 44% ± 15%, assuming 1% of the total diesel fleet is electrified in 2025.[13] For simplification purposes and consideration of the rapid decarbonization of the energy sector in Colorado, the direct emissions associated with electrified vehicles are assumed to be zero. Additionally, accounting for emissions from electric vehicles does not provide any value to comparing use cases of biodiesel; however, understanding the possible futures of electrification of the fleet does play a role in how much biodiesel/diesel could be consumed and thereby emissions produced.

To allow for the practical development of supply chains and potential in-state biodiesel capacity generation, we modeled a progressive stepwise maximum percentage of biodiesel in the overall diesel fuel mix at a 2% minimum in each year, with a maximum of 5% through 2026, 10% through 2029, 15% through 2031, and 20% through 2033 as detailed in Table S3.2 similar to existing policies observed in Pennsylvania, Kansas, and Washington.

The high percentage of biodiesel was capped at 20% in the later years of the study due to engine and storage incompatibilities and possible gelling at low temperatures for blends above 20% biodiesel. Use of blends above 20% biodiesel are less common but possible with modifications to engines and infrastructure, which is beyond the scope of this analysis.

We simulated each possible biodiesel blend volume in 1% increments in the GREET model version 1.3.0.13991 for a representative vehicle over the study period. Representative vehicles used from each class in the GREET model are shown in Table S3.3. GREET has been used by numerous studies to quantify potential transport sector emissions reductions from biodiesel consumption including a Colorado-specific study assessing implementation of a statewide clean fuel standard.[14]

In GREET the blend of biodiesel in the fuel was modified as well as the study year to account for changes in technology and federal emissions requirements for diesel vehicles' emissions. The full well-to-wheels life-cycle emissions results from the GREET model were returned on a per VMT basis for each vehicle class. Creating GREET models for each vehicle class in each study year at biodiesel blend percentage (2-20%) for each pollutant (CO_2, CO, PM, VOCs, NO_X, and SO_X), provided the respective kg per mile of pollutant emitted for each case.

The annual emissions reduction for each pollutant was compared to the use of low-sulfur diesel (LSD) in each case, also modeled in GREET as there is currently no biodiesel fuel mandate existing in Colorado. GREET simulation output for each case, representing 840 GREET model runs. Due to overall federal blending mandates for biodiesel that equate to 2% of the total diesel volume, it is possible to have fuel with or without biodiesel preblended; we assume that no biodiesel is currently in the Colorado fuel mix for comparison purposes.

We quantify the social value of emissions reduction using values published in literature for the social cost per ton of pollutant emitted as represented in Table S3.4. The total yearly social benefit of switching to a biodiesel blend percentage is the sum of total social costs for each pollutant in its respective case:

Historic values were obtained from the U.S. Department of Energy's Clean Cities Alternative Fuels Reports between 2017 and 2022 for B20 fuel vs. LSD on an energy basis. Nationally, B20 was sold for $0.12 ± $0.09 less than pure diesel (normally distributed), with prices in the Rocky Mountain Region specifically at $0.28 ± $0.20 higher per gallon of fuel (log-normally distributed). See Table S3.5 for historic fuel cost difference data[10]. To account for the reported cost difference for B20 blends, we estimate the change in cost associated with various blends as a proportion further detailed in Table S3.2.

The Colorado-specific fuel economy in miles per gallon (MPG) of each vehicle class is shown in Table S3.6 assuming a uniform uncertainty of ± 20%. We applied proportional conversion factors (Table S3.7) to account for the lower energy density of biodiesel compared to LSD. [10] We then determine the total gallons of diesel fuel consumed by each vehicle class in each year and biodiesel blend volume scenario accounting for the decreased energy intensity in each blend.

We determine the total gallons consumed in a given year for each class in that year under a specific biodiesel blend volume and determine the total additional fuel cost (or savings) to consumers of the fuel blend. The total social value of each biodiesel case in each year is the sum of the fuel cost or savings and the social value of emissions reductions.

We conducted a Monte Carlo analysis with 10,000 simulations to reflect uncertainty in the input factors both in the United States and the Rocky Mountain Region specifically: cost difference of biodiesel compared to LSD, CAGR of electrification, CAGR of each vehicle class population, MPG of each fleet class, and social cost of emissions. We also determined model correlations and conducted sensitivity analysis for each of these parameters. A more detailed description of the modeling calculations is provided in the following section.

S3.3 Colorado Biodiesel Case Study Results Summary

The results of our model showed a decrease the annual emissions of Colorado's diesel-consuming fleet for each of the studied criteria pollutants, as shown in a representative example for the year 2032 in Figure S3.1. Similar figures and model output data for each year of the study period are in Section S3.6.

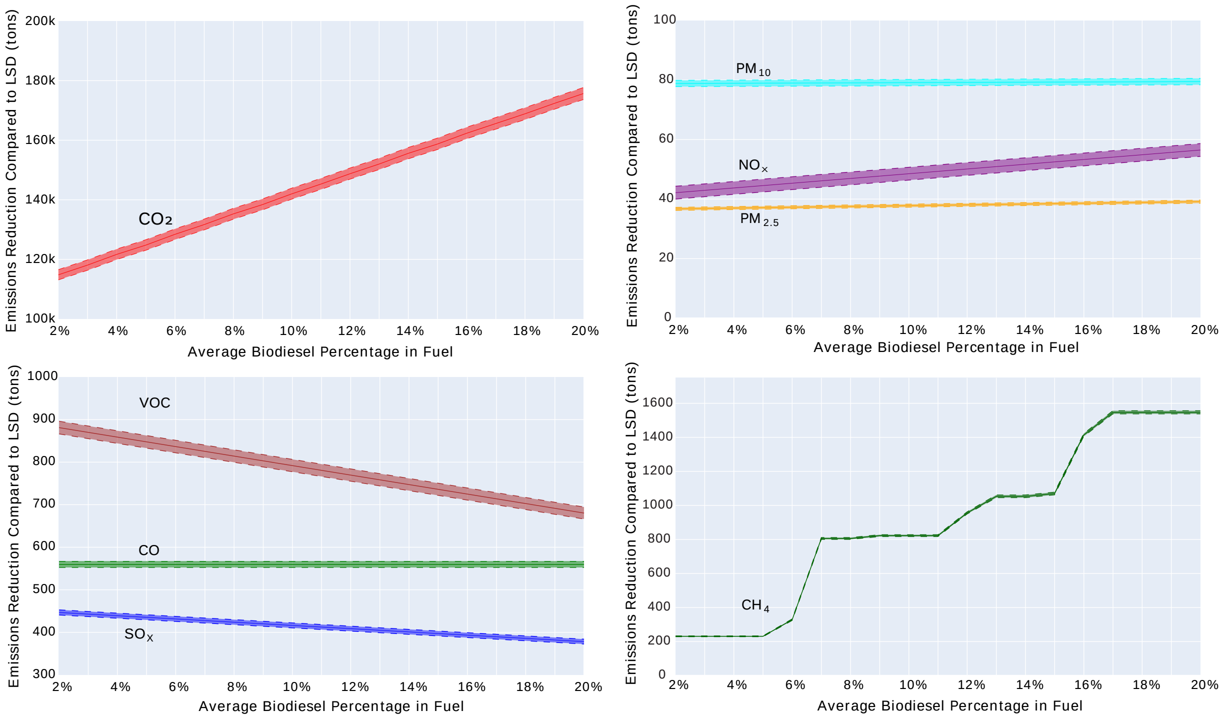

Figure S3.1. Projected emissions reductions of CO_2, $PM_{2.5}$, PM_{10}, CO, VOCs, NO_X, SO_X, and CH_4 at biodiesel blend volumes from 2% to 20% compared to the use of LSD in 2032 for the Colorado diesel fleet bound by 5% and 95% confidence intervals.

Our analysis showed that as the biodiesel volume percentage increased, the well-to-wheels emissions reduction compared to LSD for CO_2, $PM_{2.5}$, PM_{10}, NO_X, and CH_4 increased with the greatest absolute reductions seen for CO_2 and CH_4 emissions. The well-to-wheels emissions of CO remained unchanged as biodiesel blend volumes increased, while VOC and SO_X showed a decrease in well-to-wheels emissions reduction (i.e., an emissions increase) as biodiesel blend volumes increased that could be due to varied reaction chemistry effects of the fuel blend or other assumption in the GREET model. Although several studies have identified increases in NO_x emissions from the direct combustion of biodiesel, from a full well-to-wheels perspective, the overall emissions of NO_x criteria pollutants are lower than that of LSD in the combined GREET and Colorado diesel consuming fleet modeled in this study. Additionally, the results of this analysis are consistent with reported literature for decreased CO_2, CO, PM, and hydrocarbon emissions through biodiesel use.[15] An interesting step function is noted for CH_4 emissions reductions the origin of which is unclear and is likely a result of GREET model's outputs used as an input for this analysis. These results indicate that the use of biodiesel blends could lead to an overall decrease in full life-cycle emissions from the Colorado diesel fleet, even considering an increased option of fleet electrification.

While decreases in criteria pollutants would always result in a net positive social benefit, to further understand the tradeoffs that reduced emissions through biodiesel blending might have on the social value, the difference in fuel cost becomes an important factor to consider, especially in cases where B20 blends are more expensive than LSD, as is the case in the Rocky Mountain Region. Sensitivity analysis results showed a strong correlation ($\rho = 0.92$) between the cost difference between B20 and LSD and the total social net cost (i.e., cost-benefit). Full results of the sensitivity analysis are in Section S3.6. Social cost/benefit is important to consider in Colorado's specific context where biodiesel is more expensive that fossil fuel diesel. It would be impractical for a state to encourage more expensive biodiesel use where the cost of such fuel outweighs the social cost benefit. would be impractical for a state to encourage more expensive biodiesel use where the cost of such fuel outweighs the social cost benefit. To illustrate the impact that the fuel cost difference has on the social value of biodiesel use, Figure S3.2 illustrates the total social value of biodiesel use under the Rocky Mountain Region and U.S. National Average fuel price difference distributions for the representative year 2032 considering both the value added from a reduction in emissions and the positive and/or negative impact of fuel cost.

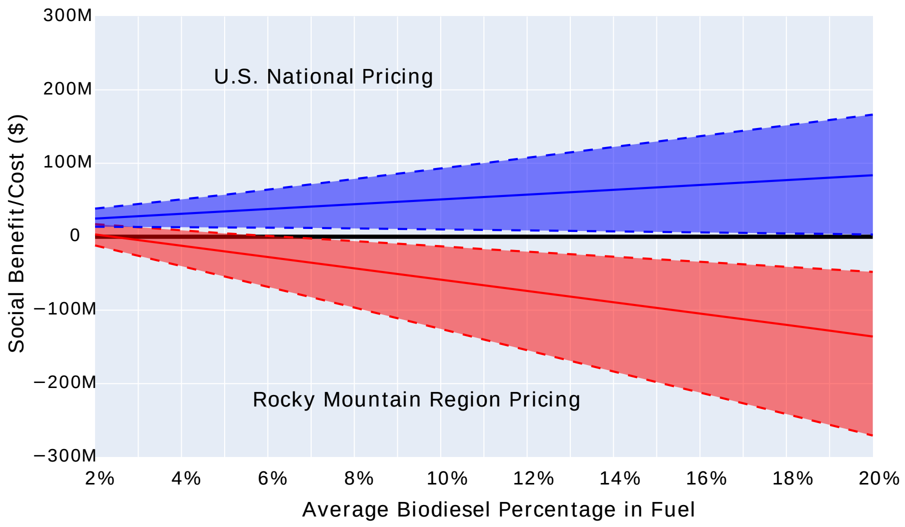

Figure S3.2. Estimated social benefit/cost expected for biodiesel blend volumes from 2% to 20% compared to the use of LSD in 2032 by the Colorado diesel fleet bound by 5% and 95% confidence intervals for both Rocky Mountain Region and U.S. National Pricing data distributions. Positive vertical axis values are benefits while negative values are costs.

As suggested by the sensitivity analysis as well as the modeling results (Figure S3.2), the social benefit of biodiesel use is highly correlated with the cost difference between fossil fuel-based diesel and biodiesel blended fuels. Under a U.S. national fuel price difference distribution, increasing the biodiesel content of the fuel leads to additional social benefits; however, the opposite is seen under the Rocky Mountain Region fuel price difference distribution. As the blend of biodiesel increases in the Rocky Mountain Region, the social benefit of emissions reductions increases; however, due to the additional cost of biodiesel and lower energy content of the fuels driving additional consumption as biodiesel volumes increase, the cost to consumers also increases creating a counteractive effect. With the fuel price differences historically seen in

the Rocky Mountain Region, it is not surprising that current biodiesel volumes in the overall fuel mix in Colorado remain less than 2%

The state of Colorado could take action to lower the cost difference between biodiesel blended fuels and LSD and approach U.S. national price differences through effective policy mechanisms that create a broader market for biodiesel. In 2022, Colorado enacted policies in four of the five categories described in Table 1 of the main text that support biodiesel use and market development. The policies include a mandate for state-owned and operated diesel vehicles and equipment to utilize B20 fuel (Biodiesel Fuel Use Mandate), grant offerings through the Colorado Office of Economic Development & International Trade (OEDIT) that can be applied to biofuel industries (Biodiesel Production Incentive), and funding for the incremental cost of alternative fuel vehicles and alternative fueling infrastructure for public fleets through the Colorado Department of Local Affairs (Biodiesel Fueling Infrastructure Incentive and Biodiesel Fleet Development). Despite this legislation, biodiesel use remains low, and no biodiesel production currently operates in Colorado. To provide perspective on new policies that could be adopted in Colorado or existing policies that could be modified to achieve the social benefits identified in this study, we assessed the relationship between policy mechanisms and state-level per capita biodiesel consumption and total biodiesel production, shown in the main text.

S3.4 Colorado Biodiesel Case Study Methodology Detailed Modeling Calculations Description

We used Equation 1 to determine the VMT of each vehicle class, i, in year j, or $VMT_{i,j}$ based on 2020 data from the Colorado Medium and Heavy Duty (M/HD) Vehicle Study.[9]

$$VMT_{i,j} = VMT_{i,j-1} \times CAGR_i \qquad \textbf{Eq. 1}$$

where $CAGR_i$ represents the compound annual growth rate of the VMT for vehicle class, i [9]. The 2020 VMT for each class as well as the corresponding CAGR and uniform uncertainty range utilized in the model are shown in Table S3.1.

Table S3.1 2020 VMT data for the diesel fuel-powered vehicles in Colorado's fleet and the CAGR for each. [9]

Vehicle Class	2020 VMT	CAGR	Uniform Uncertainty Range
Heavy Duty	1,402,000,000	0.85%	±10% of CAGR
Medium Duty	885,000,000	2.08%	±10% of CAGR
Bus	164,000,000	1.34%	±10% of CAGR
Light Duty	4,206,000,000	-0.42%	±10% of CAGR
Total	6,657,000,000	N/A	

To consider the electrification of the fleet,[13] projections were mirrored with a uniformly distributed CAGR of 44% ± 15%, assuming 1% of the total diesel fleet is electrified in 2025,[13] The percentage of the fleet that is electrified in each year, E_j, informs the VMT traveled in each respective year by diesel-powered vehicles in each class, $DVMT_{i,j}$:

$$DVMT_{i,j} = VMT_{i,j} \times (1 - E_j) \qquad \textbf{Eq. 2}$$

For simplification purposes and consideration of the rapid decarbonization of the energy sector in Colorado, the direct emissions associated with electrified vehicles are assumed to be zero.

We modeled a progressive stepwise maximum percentage of biodiesel in the overall diesel fuel mix at a 2% minimum in each year, with a maximum of 5% through 2026, 10% through 2029, 15% through 2031, and 20% through 2033 as detailed in Table S3.2.

Table S3.2 Ranges of biodiesel volume percentage in the overall diesel fuel mix in Colorado used in this study.

Year	Low % of Biodiesel in Overall Fuel Mix, k	High % of Biodiesel in Overall Fuel Mix, k
2024	2%	5%
2025	2%	5%
2026	2%	5%
2027	2%	10%
2028	2%	10%
2029	2%	10%
2030	2%	15%

2031	2%	15%
2032	2%	20%
2033	2%	20%

We simulated each possible biodiesel blend volume in 1% increments in the GREET model version 1.3.0.13991 for a representative vehicle over the study period. Representative vehicles used from each class in the GREET model are shown in Table S3.3.

Table S3.3. Name of representative vehicle used for each class in this study and its name in the GREET model.

**Vehicle Class**	**GREET Model Designation**
Heavy Duty	HDV: Combination Short Haul Truck CIDI
Medium Duty	HDV : MHD Vocational Vehicle CIDI
Bus	HDV: LHD Transit Bus CIDI
Light Duty	HDV : LHD Vocational Vehicle CIDI

In GREET the blend of biodiesel in the fuel was modified as well as the study year to account for changes in technology and federal emissions requirements for diesel vehicles' emissions. The full well-to-wheels life-cycle emissions results from the GREET model were returned on a per VMT basis for each vehicle class. Creating GREET models for each vehicle class, *i*, in year *j*, at biodiesel blend percentage, *k* (0-20%), for each pollutant, *l* (CO_2, CO, PM, VOCs, NO_X, and SO_X), provided the respective kg per mile of pollutant emitted for each case and is represented by $P_{i,j,k,l}$. The total quantity of pollutants emitted (in kg) in each case, $TP_{i,j,k,l}$,, is:

$$TP_{i,j,k,l} = P_{i,j,k,l} \times DMVT_{i,j} \quad \textbf{Eq. 3}$$

The annual emissions reduction for each pollutant, *i*, was compared to the use of low-sulfur diesel (LSD) in each case, also modeled in GREET as there is currently no biodiesel fuel mandate existing in Colorado. Due to overall federal blending mandates for biodiesel that equate to 2% of the total diesel volume, it is possible to have fuel with or without biodiesel preblended; we assume that no biodiesel is currently in the Colorado fuel mix for comparison purposes. The annual emissions reduction for each pollutant in each case $RP_{i,j,k,l}$ is determined by Equation 4, where $TP_{i,j,LSD,l}$ represents the respective comparison case where no biodiesel is blended in the fuel.

$$RP_{i,j,k,l} = TP_{i,j,LSD,l} - TP_{i,j,k,l} \quad \textbf{Eq. 4}$$

We quantify the social value of emissions reduction using values published in literature for the social cost per ton of pollutant emitted, SC_l, as represented in Table S3.4.

Table S3.4 The social cost of pollutants considered in this study per ton emitted.

**Pollutant, *l***	**Average Social Cost, SC_l ($/metric ton emitted)**	**Social Cost Distribution Type**	**Standard Deviation or Range***	**Citation**
CO_2	$51	Log-Normal	$89	[16]
PM	$66,784	Uniform	$34,430 - $99,138*	[17]
CO	$1,964	Log-Normal	$1,373	[18]
VOCs	$2,483	Uniform	$1,258 - $3,708*	[17]
NO_X	$10,859	Uniform	$5,827 - $15,891*	[17]
SO_X	$8,506	Log-Normal	$12,739	[18]
CH_4	$1,485	Log-Normal	$2,504	[16]

* indicates where a range is applied to the distribution instead of a standard deviation.

The annual total social cost of each case for each pollutant, $TSC_{i,j,k,l}$, is:

$$TSC_{i,j,k,l} = RP_{i,j,k,l} \times SC_l \quad \textbf{Eq. 5}$$

The total yearly social benefit, $YSB_{j,k}$, of switching to a biodiesel blend percentage, k, is the sum of total social costs for each pollutant in its respective case:

$$YSB_{j,k} = \sum_{i,l}^{n} TSC_{i,j,k,l} \quad \textbf{Eq. 6}$$

Historic values were obtained from the U.S. Department of Energy's Clean Cities Alternative Fuels Reports between 2017 and 2022 for B20 fuel vs. LSD on an energy basis. Nationally, B20 was sold for $0.12 ± $0.09 less than pure diesel (normally distributed), with prices in the Rocky Mountain Region specifically at $0.28 ± $0.20 higher per gallon of fuel (log-normally distributed). See Table S3.5 for historic fuel cost difference data.[10]

Table S3.5. Historic Rocky Mountain Region and U.S. Price Difference Data between 2017 and 2022.

**Clean Cities Alternative Fuel Prices Report Date**	**Rocky Mountain Region Price Difference for B20 vs. Diesel**	**U.S. Price Difference for B20 vs. Diesel**	**Source**
July, 2022	$0.17	$(0.20)	[19]
April, 2022	$0.25	$(0.35)	[20]
January, 2022	$0.08	$(0.13)	[21]
October, 2021	$0.19	$(0.12)	[22]
July, 2021	$0.14	$(0.15)	[23]
April, 2021	$(0.12)	$(0.23)	[24]
January, 2021	$0.55	$(0.17)	[25]
October, 2020	$0.89	$(0.06)	[26]
July, 2020	$0.49	$0.08	[27]
April, 2020	$0.20	$(0.20)	[28]

January, 2020	$0.35	$(0.10)	29
October, 2019	$0.18	$(0.15)	30
July, 2019	$0.31	$(0.12)	31
April, 2019	$0.34	$(0.15)	32
January, 2019	$0.24	$(0.12)	33
October, 2018	$0.20	$(0.21)	34
July, 2018	$0.34	$(0.12)	35
April, 2018	$0.39	$(0.10)	36
January, 2018	$0.38	$(0.06)	37
October, 2017	$0.23	$(0.03)	38
July, 2017	$0.17	$0.07	39
April, 2017	$0.12	$(0.01)	40
Average	$0.28	$(0.12)	
Standard Deviation	$0.20	$0.09	
Median	$0.24	$(0.12)	

To account for the reported cost difference for B20 blends, we estimate the change in cost associated with various blends as a proportion:

$$\frac{C_{20}}{B_{20}} = \frac{C_k}{B_k} \qquad \text{Eq. 7}$$

where C_{20} represents the cost of B20 fuel, B_{20} represents the blending of 20% biodiesel in the fuel mix, C_k represents the cost to be solved for each biodiesel blend of (2%-19%), and B_k represents the respective biodiesel blend volume.

The Colorado-specific fuel economy in miles per gallon (MPG) of each vehicle class is shown in Table S3.6.

Table S3.6. Blended MPG values for each vehicle class for 2020-2030 and 2030-2040.[9]

Vehicle Class	2020-2030 MPG	2030-2040 MPG
Heavy Duty	5.84	6.90
Medium Duty	9.53	10.4
Bus	6.50	7.1
Light Duty	15.32	18.6

We assumed a uniform uncertainty of ± 20% in the $MPG_{i,j}$ value. We applied proportional conversion factors, X_k, (Table S3.7) to account for the lower energy density of biodiesel compared to LSD, representing 1.1 gallons of B100 or 1.02 gallons of B20 per 1 gallon of LSD.[10]

Table S3.7. Biodiesel blend conversion factors to gallons of fossil fuel diesel.

Biodiesel Blend	Biodiesel %	Gallons of Diesel Equivalent
B2	2.00	1.002
B3	3.00	1.003
B4	4.00	1.004
B5	5.00	1.005
B6	6.00	1.006
B7	7.00	1.007
B8	8.00	1.008
B9	9.00	1.009
B10	10.00	1.010
B11	11.00	1.011
B12	12.00	1.012
B13	13.00	1.013
B14	14.00	1.014
B15	15.00	1.015
B16	16.00	1.016
B17	17.00	1.017
B18	18.00	1.018
B19	19.00	1.019
B20	20.00	1.020

Using the $MPG_{i,j}$. and X_k with the calculated $DVMT_{i,j}$ (Equation 2), the total gallons of diesel fuel consumed, $G_{i,j,k}$ by each vehicle class, *i*, in each year, *j*, in each biodiesel blend volume scenario, *k*, were calculated accounting for the decreased energy intensity in the biodiesel blend:

$$G_{i,j,k} = \frac{DMVT_{i,j}}{MPG_{i,j}} \times X_k \quad \textbf{Eq. 8}$$

The total gallons consumed in a given year, $TG_{j,k}$, is the sum of the total gallons consumed by each class in that year under a specific biodiesel blend volume. The total additional fuel cost (or savings) to consumers of the fuel blend, $CTG_{j,k}$, is:

$$CTG_{j,k} = TG_{j,k} \times C_k \quad \textbf{Eq. 9}$$

The total social value of each biodiesel case in each year, $SV_{j,k}$, is:

$$SV_{j,k} = YSC_{j,k} + CTG_{j,k} \quad \textbf{Eq. 10}$$

S3.5. Biodiesel Fuel Price Distributions

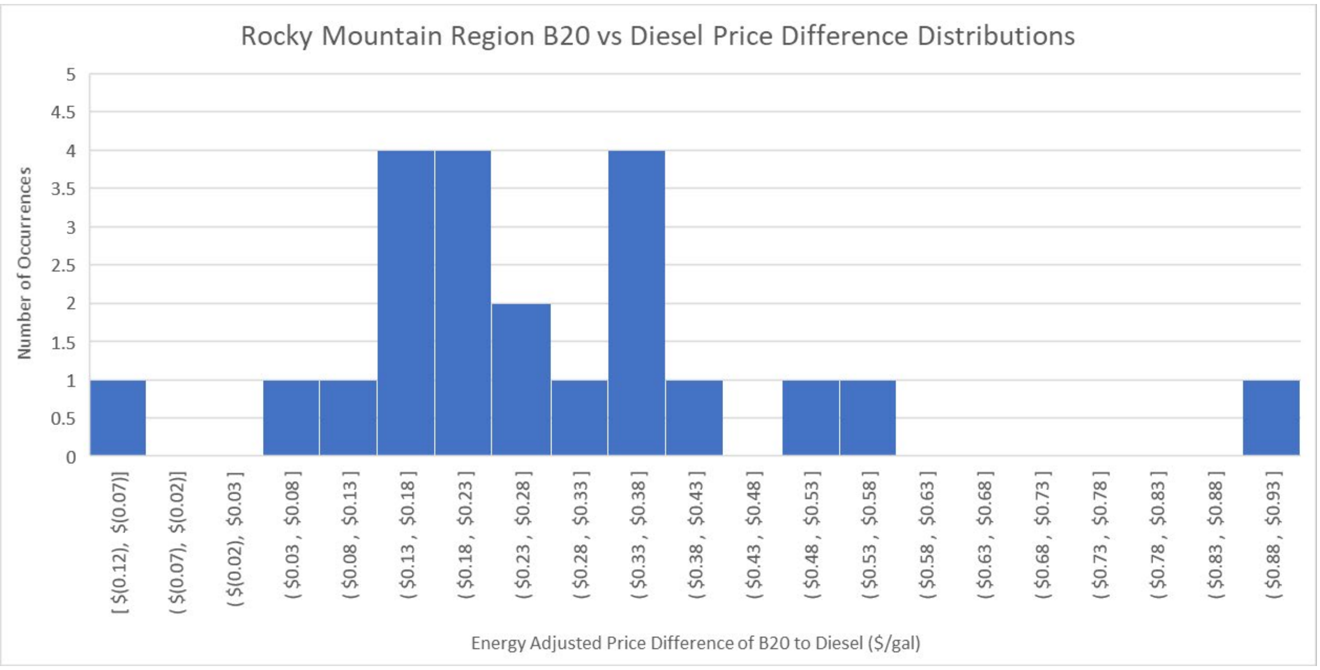

Figure S3.3. Energy adjusted price difference of B20 compared to fossil fuel diesel in the Rocky Mountain Region from 2017 to 2022. See references in Table S3.3.

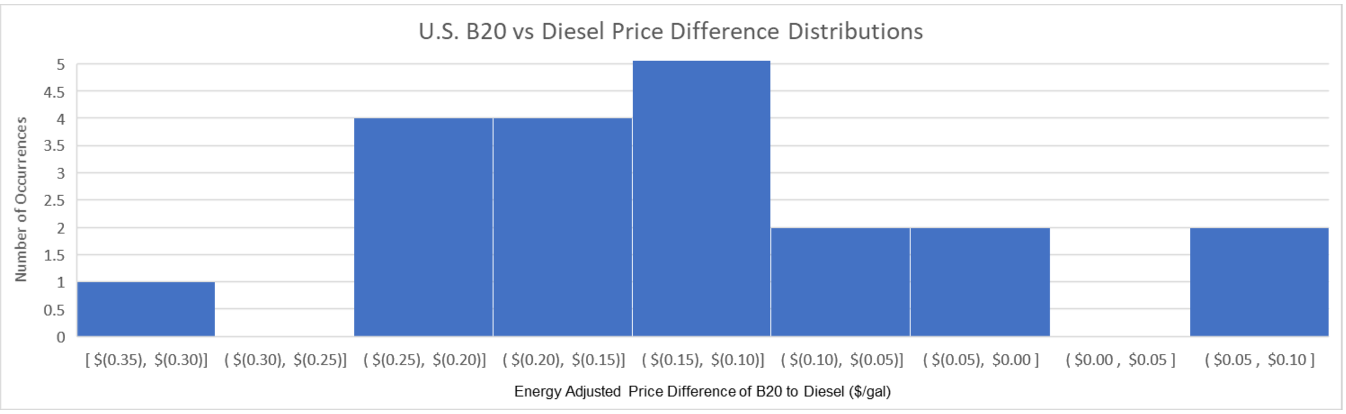

Figure S3.4. Energy adjusted price difference of B20 compared to fossil fuel diesel in the United States from 2017 to 2022. See references in Table S3.3.

S3.6. Additional Emissions Reduction Figures

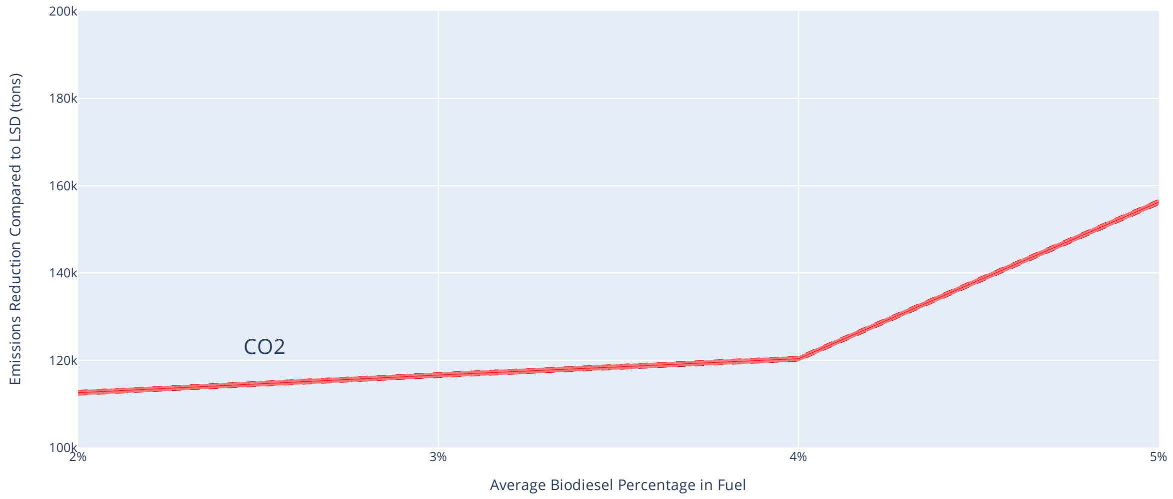

Figure S3.5a. Projected emissions reductions in 2024 for CO_2 at biodiesel blend volumes from 2% to 5% compared to the use of LSD for the Colorado diesel fleet bound by 5% and 95% confidence intervals.

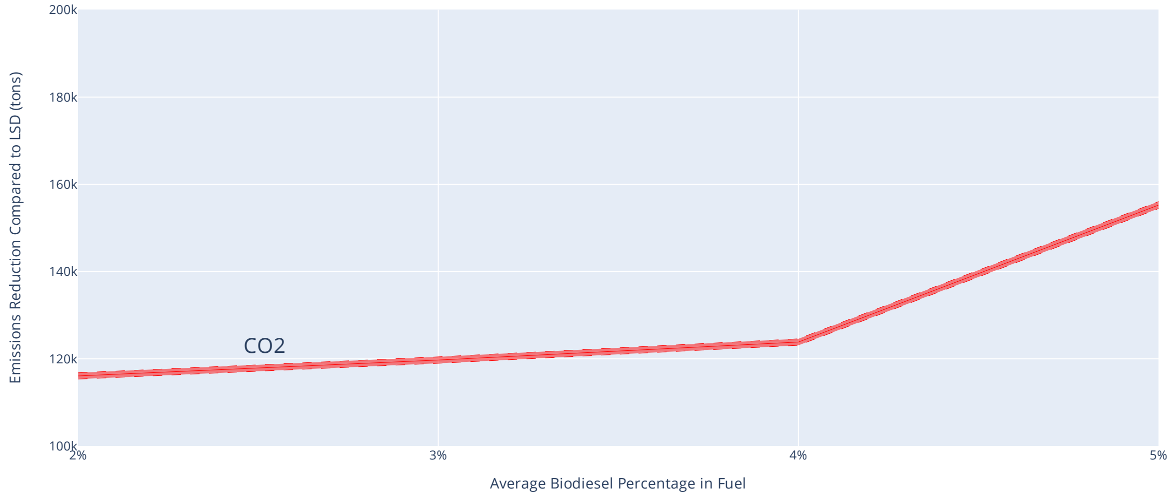

Figure S3.5b. Projected emissions reductions in 2025 for CO_2 at biodiesel blend volumes from 2% to 5% compared to the use of LSD for the Colorado diesel fleet bound by 5% and 95% confidence intervals.

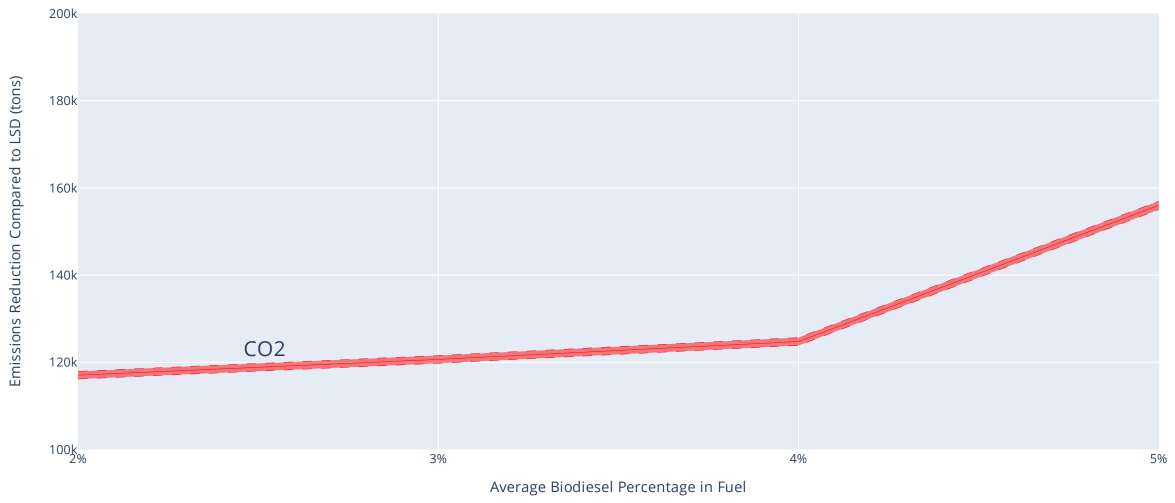

Figure S3.5c. Projected emissions reductions in 2026 for CO_2 at biodiesel blend volumes from 2% to 5% compared to the use of LSD for the Colorado diesel fleet bound by 5% and 95% confidence intervals.

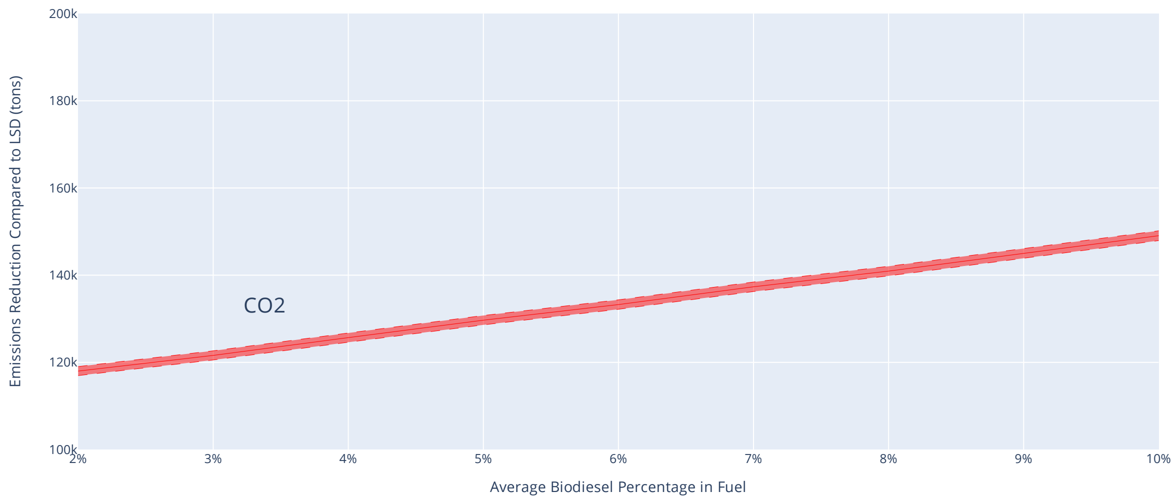

Figure S3.5d. Projected emissions reductions in 2027 for CO_2 at biodiesel blend volumes from 2% to 10% compared to the use of LSD for the Colorado diesel fleet bound by 5% and 95% confidence intervals.

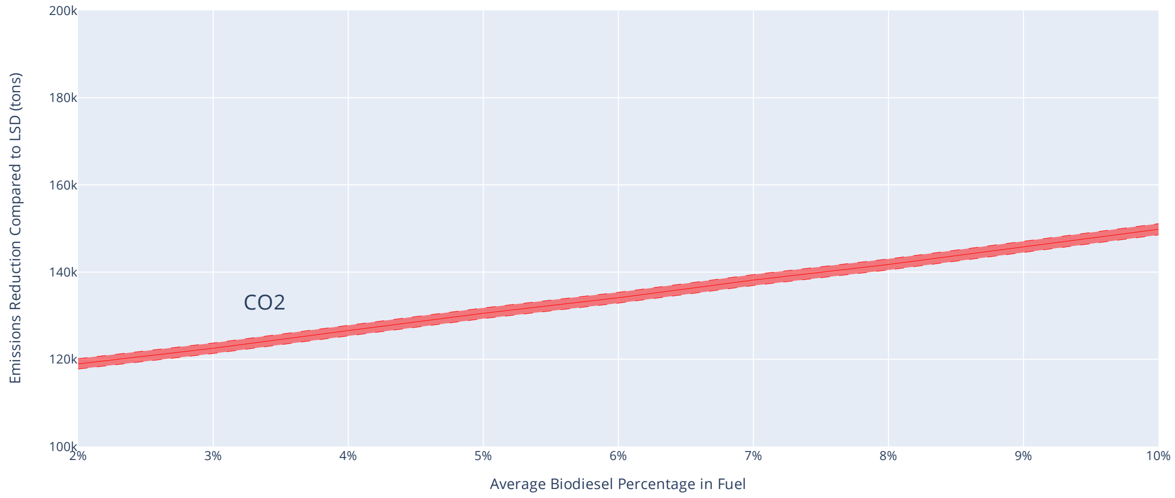

Figure S3.5e. Projected emissions reductions in 2028 for CO_2 at biodiesel blend volumes from 2% to 10% compared to the use of LSD for the Colorado diesel fleet bound by 5% and 95% confidence intervals.

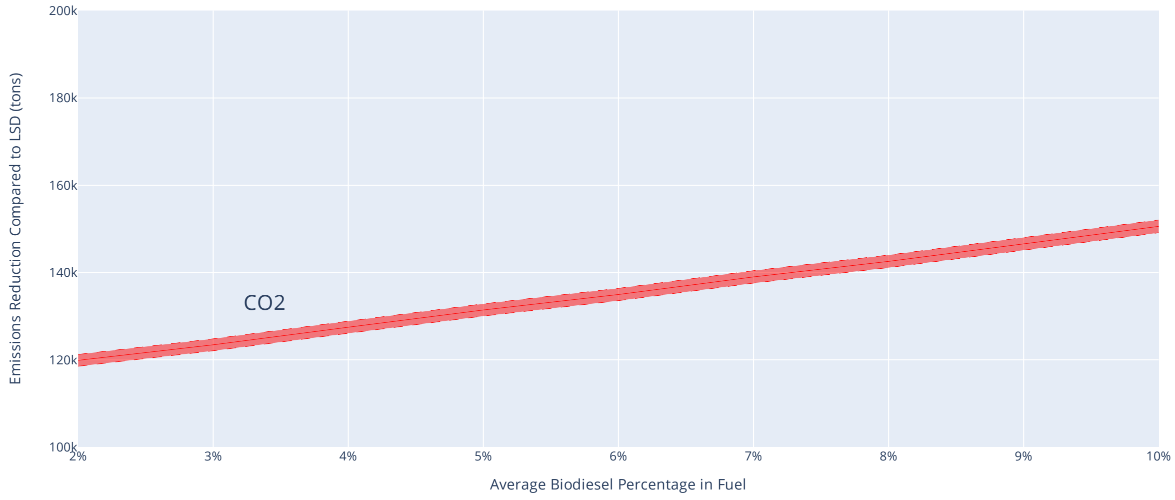

Figure S3.5f. Projected emissions reductions in 2029 for CO_2 at biodiesel blend volumes from 2% to 10% compared to the use of LSD for the Colorado diesel fleet bound by 5% and 95% confidence intervals.

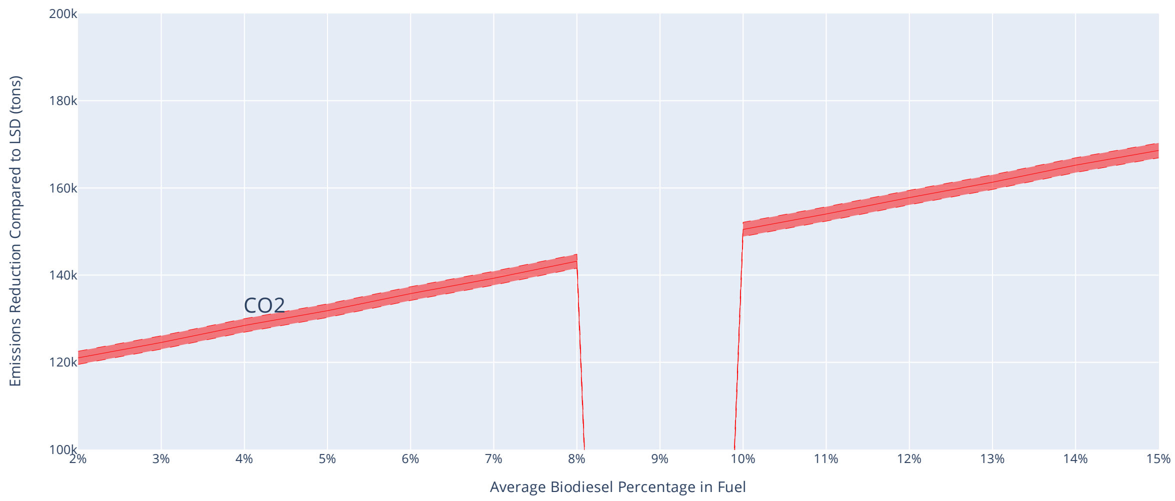

Figure S3.5g. Projected emissions reductions in 2030 for CO_2 at biodiesel blend volumes from 2% to 15% compared to the use of LSD for the Colorado diesel fleet bound by 5% and 95% confidence intervals. Note: An anomaly exists in GREET for a B9 blend in 2030.

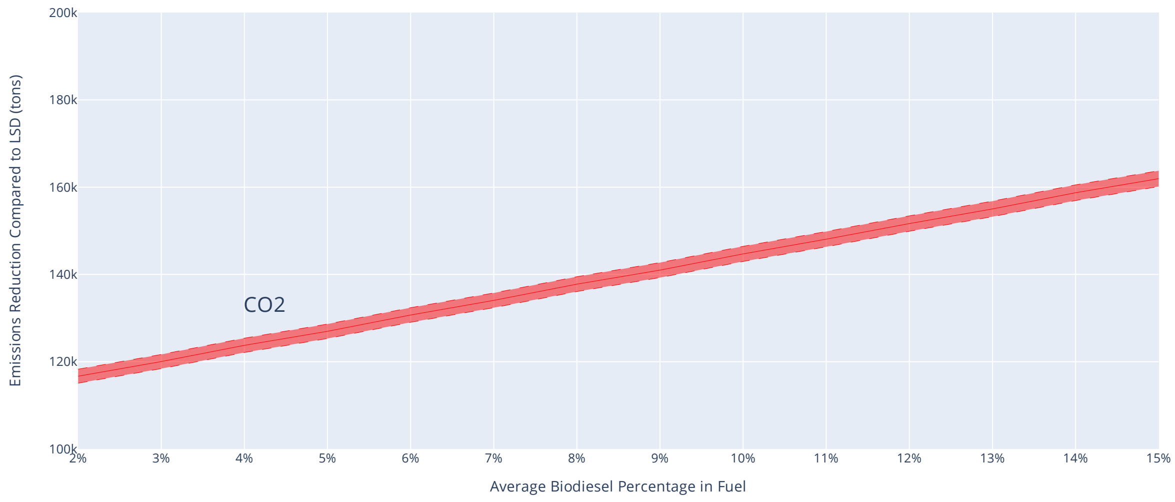

Figure S3.5h. Projected emissions reductions in 2031 for CO_2 at biodiesel blend volumes from 2% to 15% compared to the use of LSD for the Colorado diesel fleet bound by 5% and 95% confidence intervals.

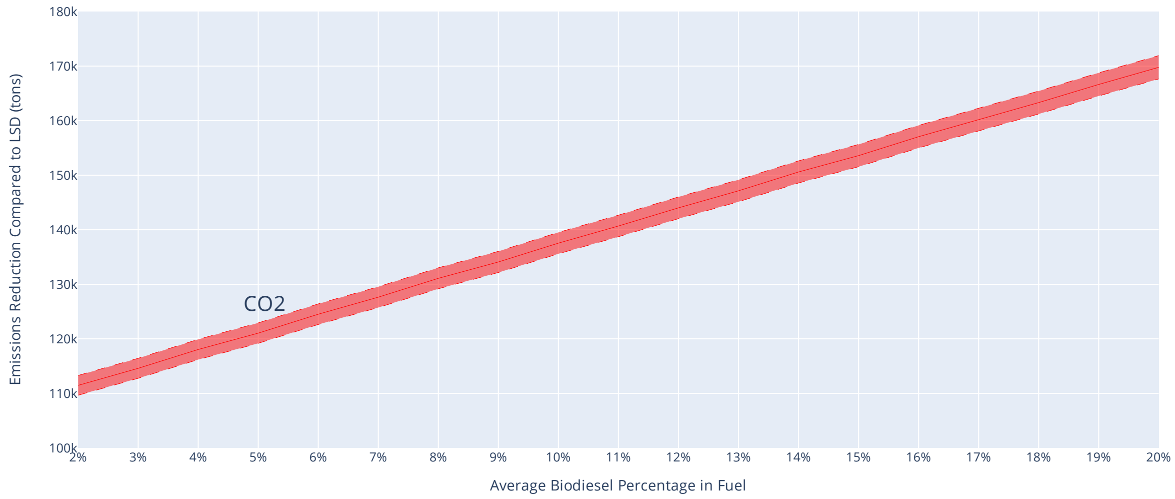

Figure S3.5i. Projected emissions reductions in 2033 for CO_2 at biodiesel blend volumes from 2% to 20% compared to the use of LSD for the Colorado diesel fleet bound by 5% and 95% confidence intervals.

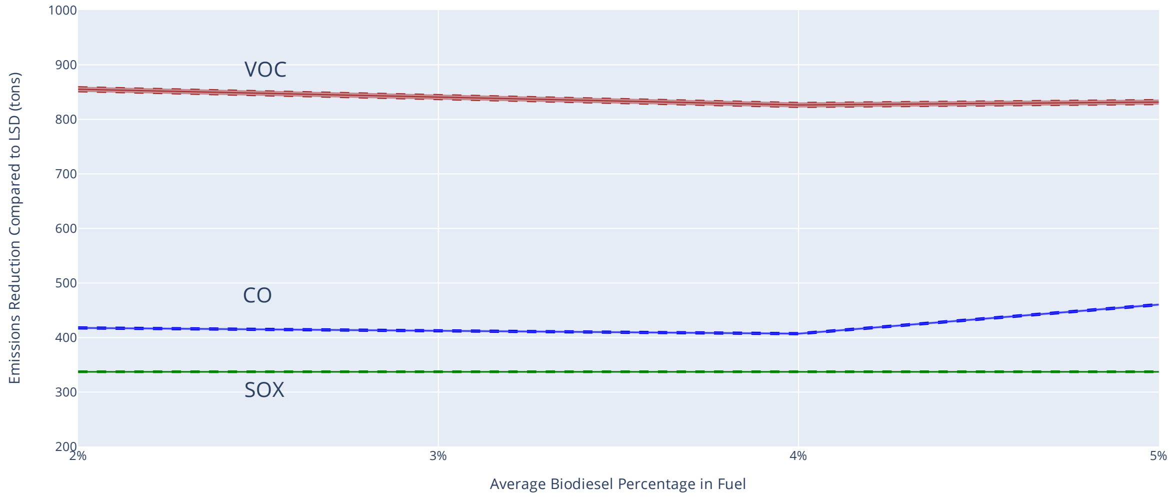

Figure S3.5j. Projected emissions reductions in 2024 for VOC, CO, and SO_X at biodiesel blend volumes from 2% to 5% compared to the use of LSD for the Colorado diesel fleet bound by 5% and 95% confidence intervals.

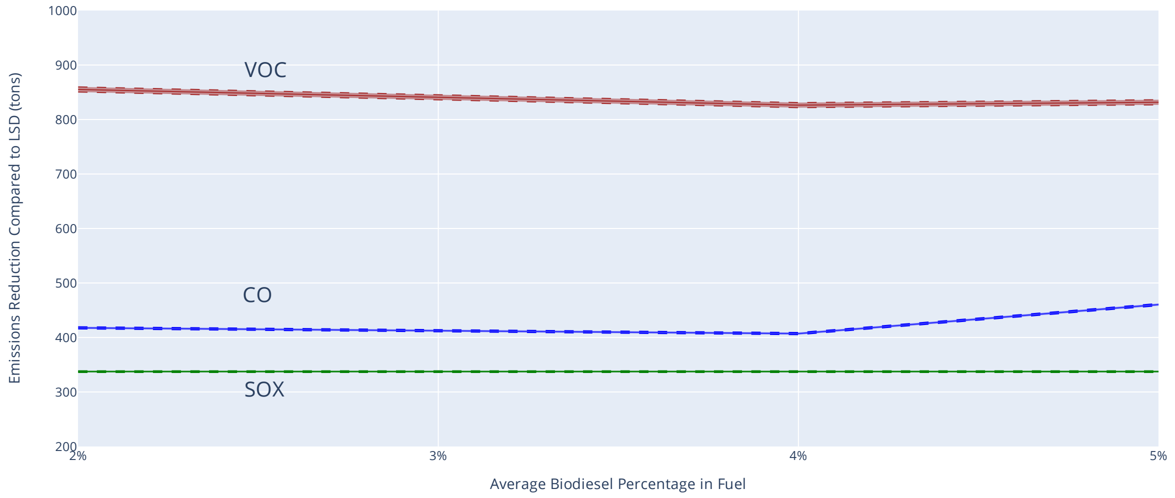

Figure S3.5k. Projected emissions reductions in 2025 for VOC, CO, and SO_X at biodiesel blend volumes from 2% to 5% compared to the use of LSD for the Colorado diesel fleet bound by 5% and 95% confidence intervals.

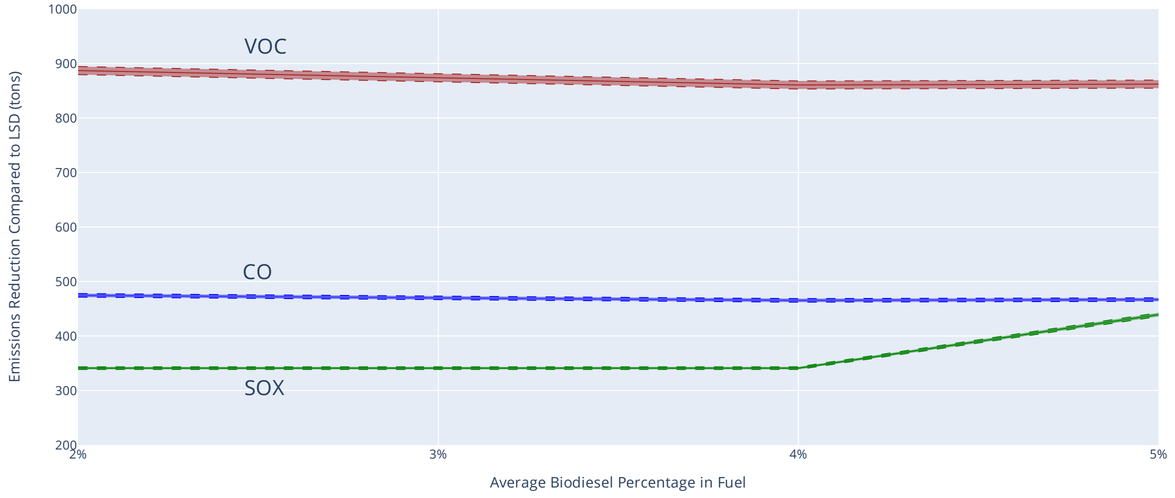

Figure S3.5l. Projected emissions reductions in 2026 for VOC, CO, and SO_X at biodiesel blend volumes from 2% to 5% compared to the use of LSD for the Colorado diesel fleet bound by 5% and 95% confidence intervals.

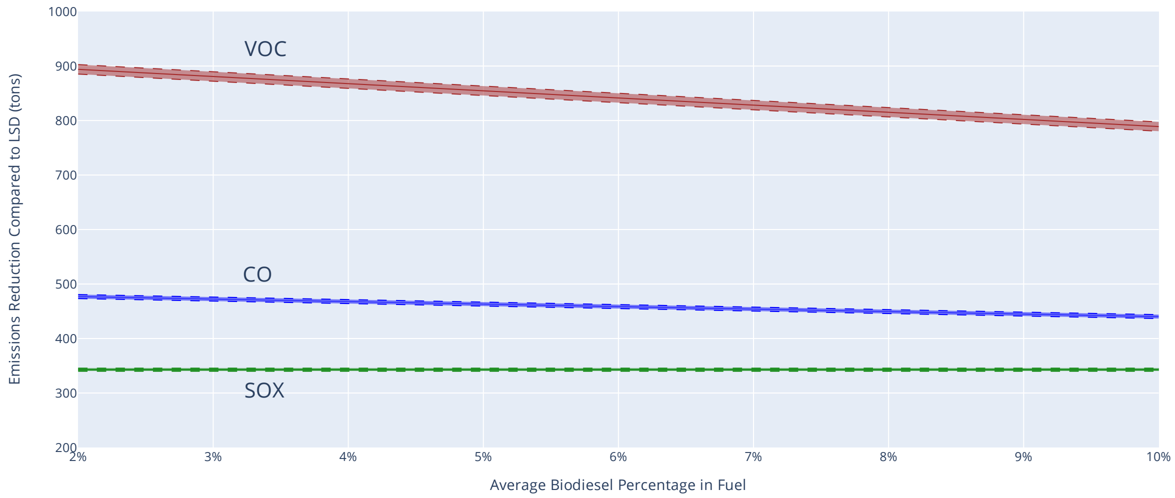

Figure S3.5m. Projected emissions reductions in 2027 for VOC, CO, and SO_X at biodiesel blend volumes from 2% to 10% compared to the use of LSD for the Colorado diesel fleet bound by 5% and 95% confidence intervals.

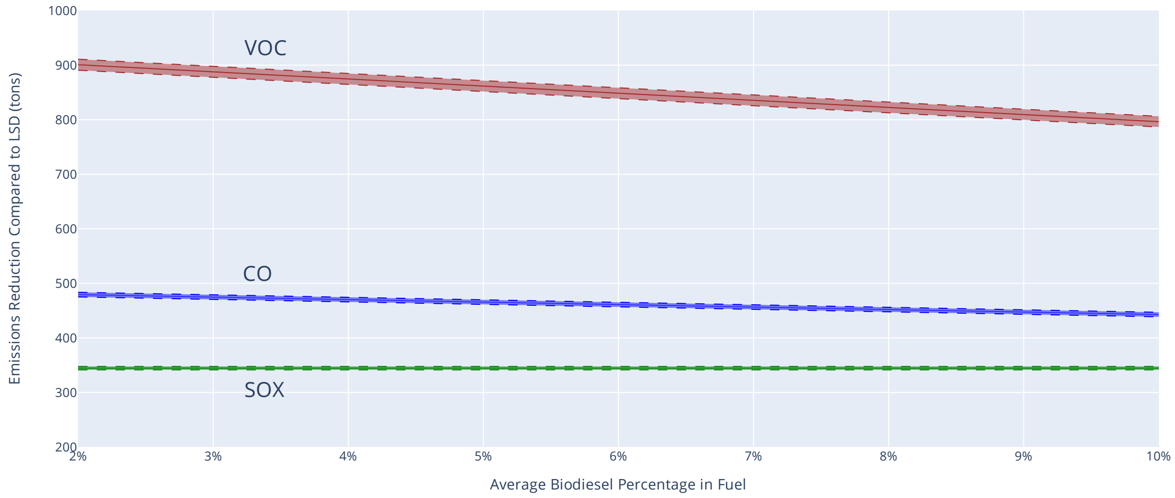

Figure S3.5n. Projected emissions reductions in 2028 for VOC, CO, and SO_X at biodiesel blend volumes from 2% to 10% compared to the use of LSD for the Colorado diesel fleet bound by 5% and 95% confidence intervals.

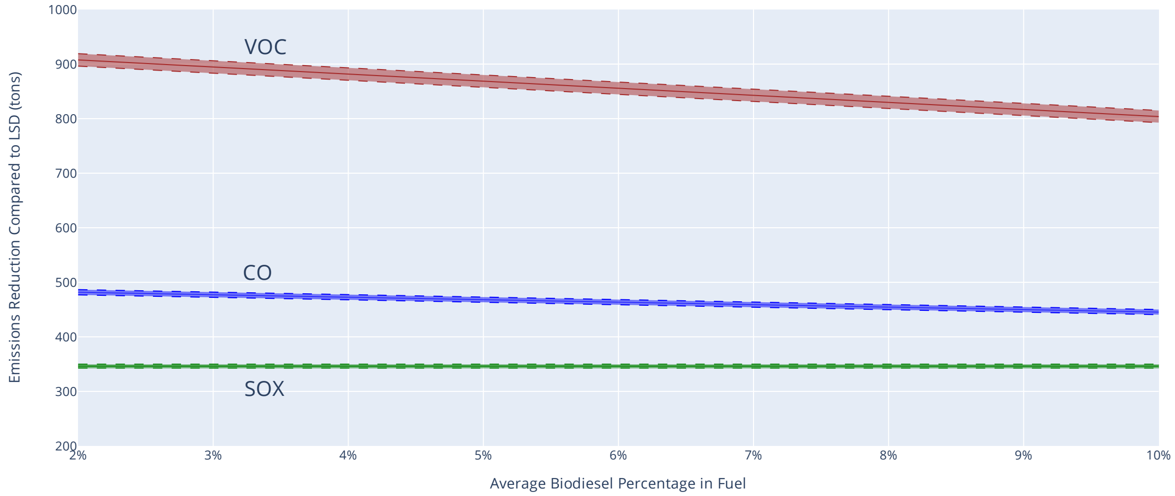

Figure S3.5o. Projected emissions reductions in 2029 for VOC, CO, and SO_X at biodiesel blend volumes from 2% to 10% compared to the use of LSD for the Colorado diesel fleet bound by 5% and 95% confidence intervals.

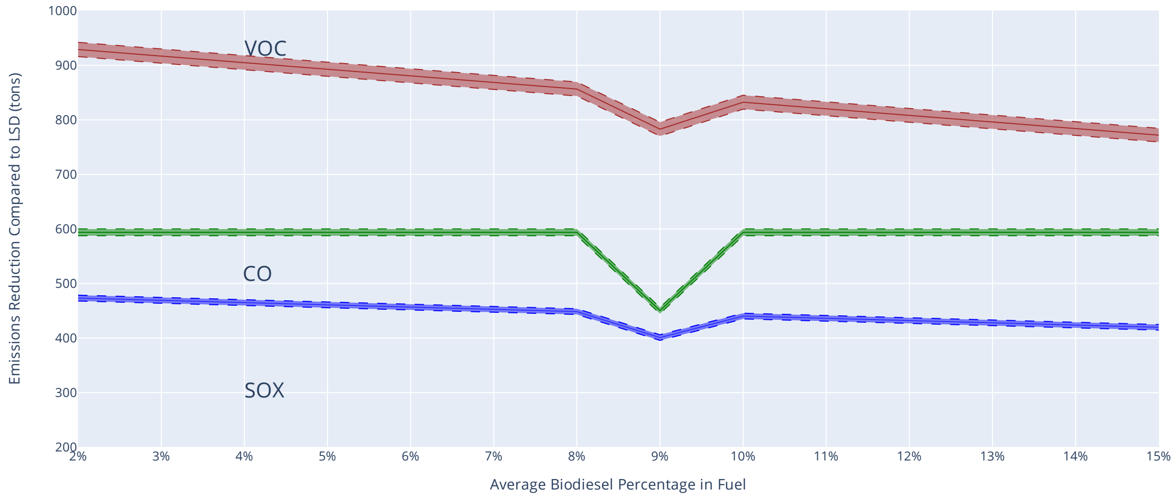

Figure S3.5p. Projected emissions reductions in 2030 for VOC, CO, and SO_X at biodiesel blend volumes from 2% to 15% compared to the use of LSD for the Colorado diesel fleet bound by 5% and 95% confidence intervals. Note: An anomaly exists in GREET for a B9 blend in 2030

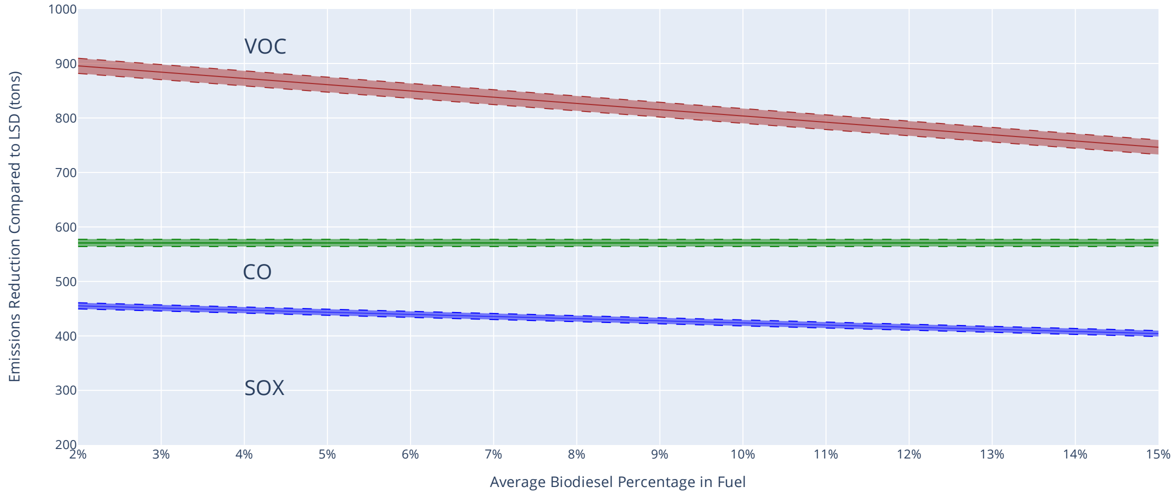

Figure S3.5q. Projected emissions reductions in 2031 for VOC, CO, and SO_X at biodiesel blend volumes from 2% to 15% compared to the use of LSD for the Colorado diesel fleet bound by 5% and 95% confidence intervals.

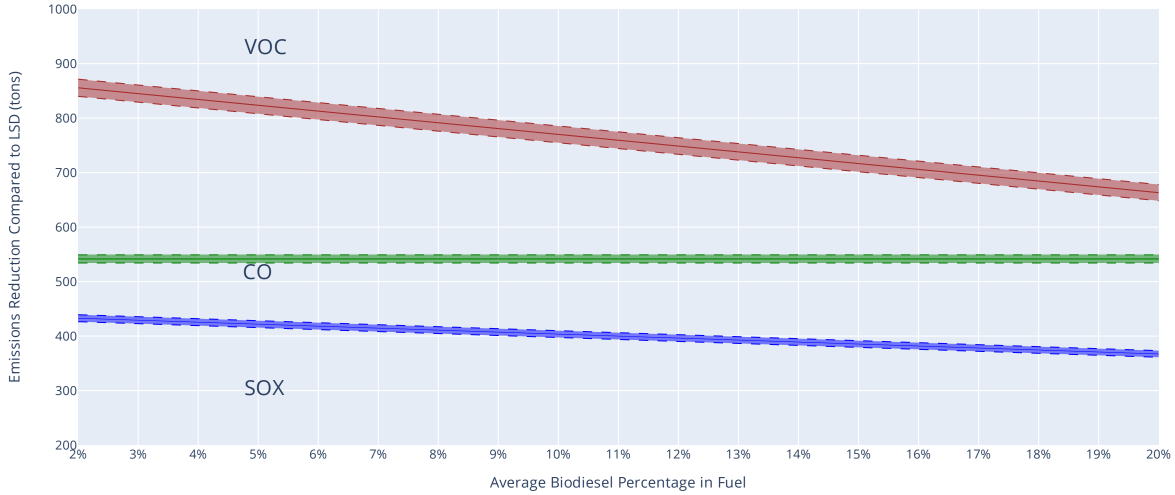

Figure S3.5r. Projected emissions reductions in 2033 for VOC, CO, and SO_X at biodiesel blend volumes from 2% to 20% compared to the use of LSD for the Colorado diesel fleet bound by 5% and 95% confidence intervals.

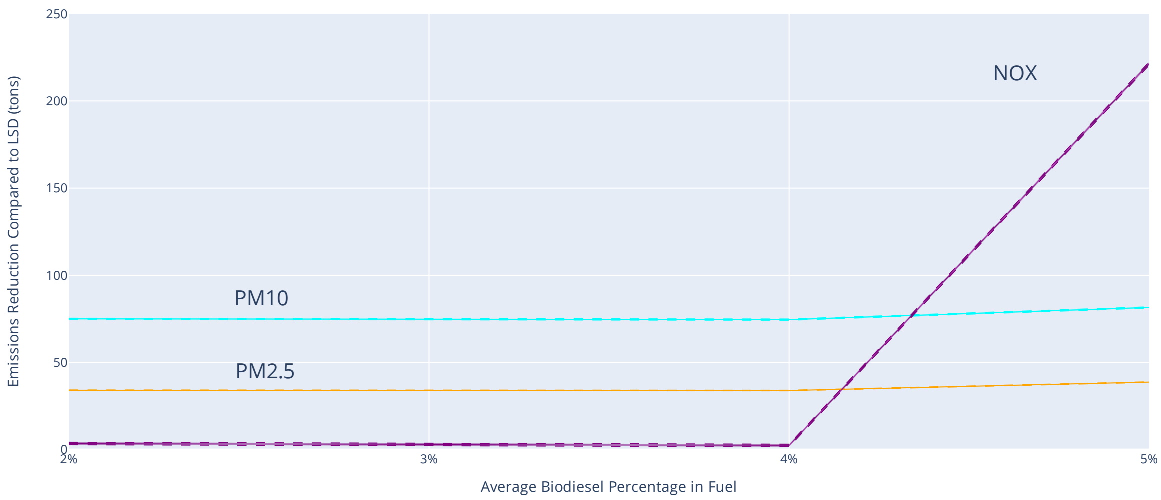

Figure S3.5s. Projected emissions reductions in 2024 for $PM_{2.5}$, PM_{10}, and NO_X at biodiesel blend volumes from 2% to 5% compared to the use of LSD for the Colorado diesel fleet bound by 5% and 95% confidence intervals.

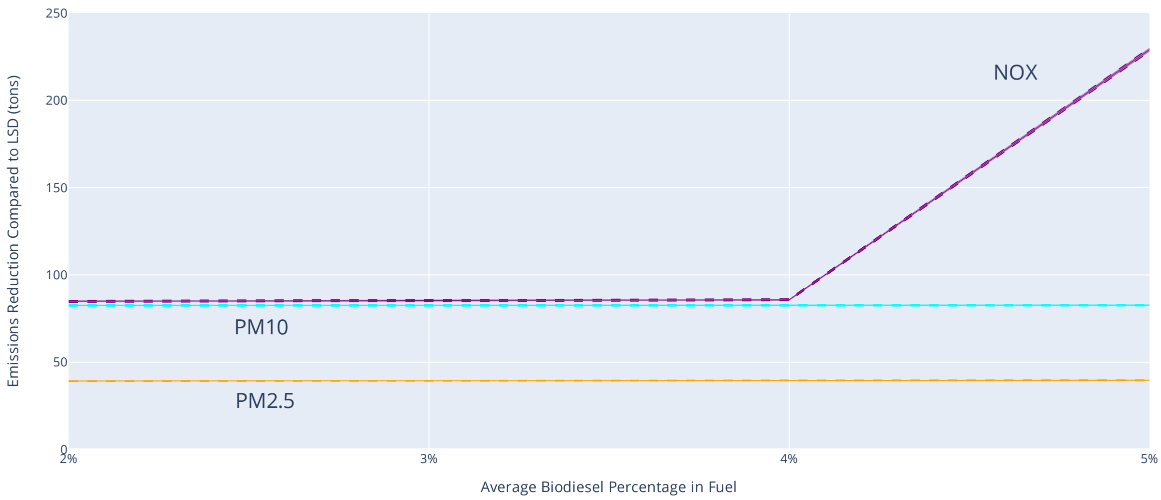

Figure S3.5t. Projected emissions reductions in 2025 for $PM_{2.5}$, PM_{10}, and NO_X at biodiesel blend volumes from 2% to 5% compared to the use of LSD for the Colorado diesel fleet bound by 5% and 95% confidence intervals.

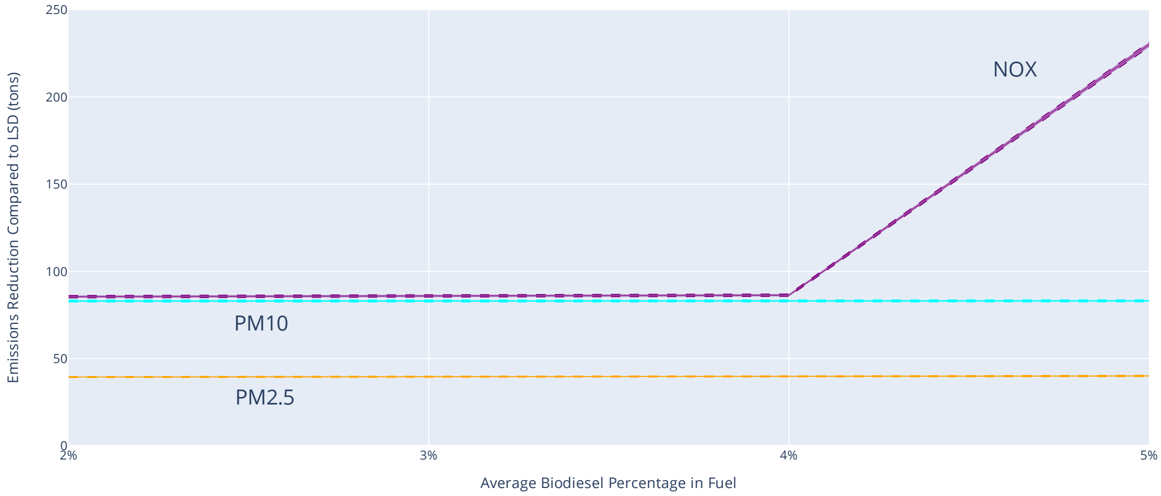

Figure S3.5u. Projected emissions reductions in 2026 for $PM_{2.5}$, PM_{10}, and NO_X at biodiesel blend volumes from 2% to 5% compared to the use of LSD for the Colorado diesel fleet bound by 5% and 95% confidence intervals.

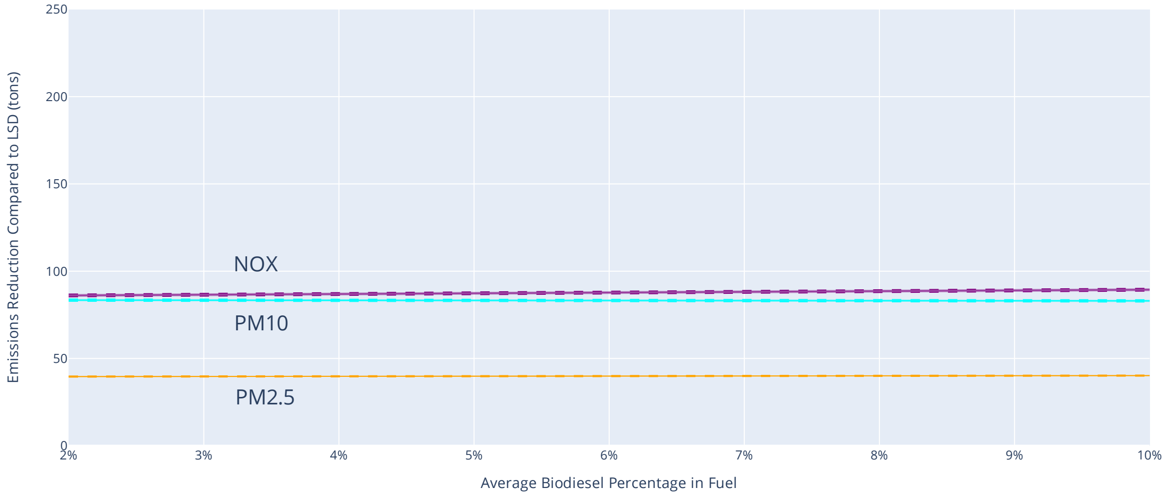

Figure S3.5v. Projected emissions reductions in 2027 for $PM_{2.5}$, PM_{10}, and NO_X at biodiesel blend volumes from 2% to 10% compared to the use of LSD for the Colorado diesel fleet bound by 5% and 95% confidence intervals.

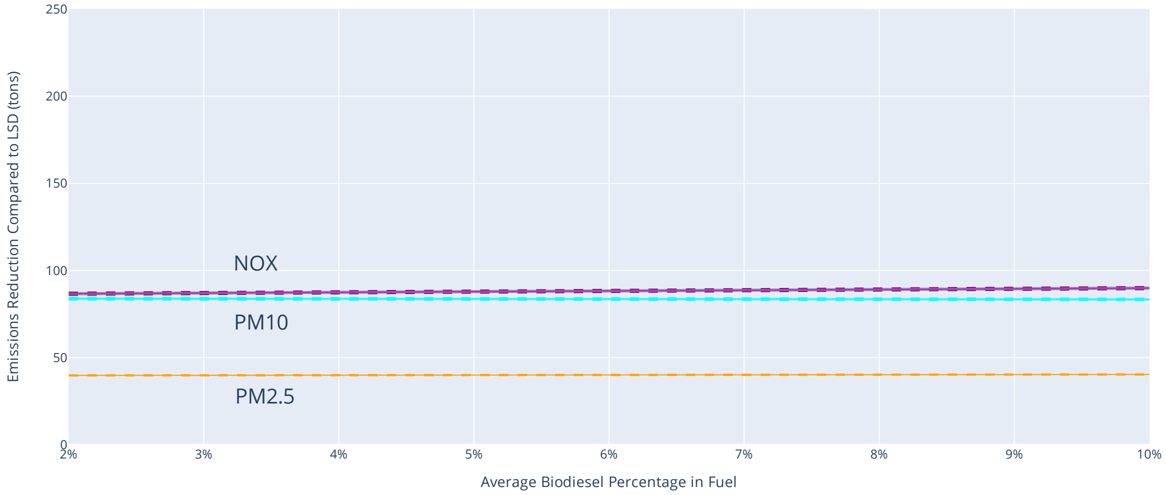

Figure S3.5w. Projected emissions reductions in 2028 for $PM_{2.5}$, PM_{10}, and NO_X at biodiesel blend volumes from 2% to 10% compared to the use of LSD for the Colorado diesel fleet bound by 5% and 95% confidence intervals.

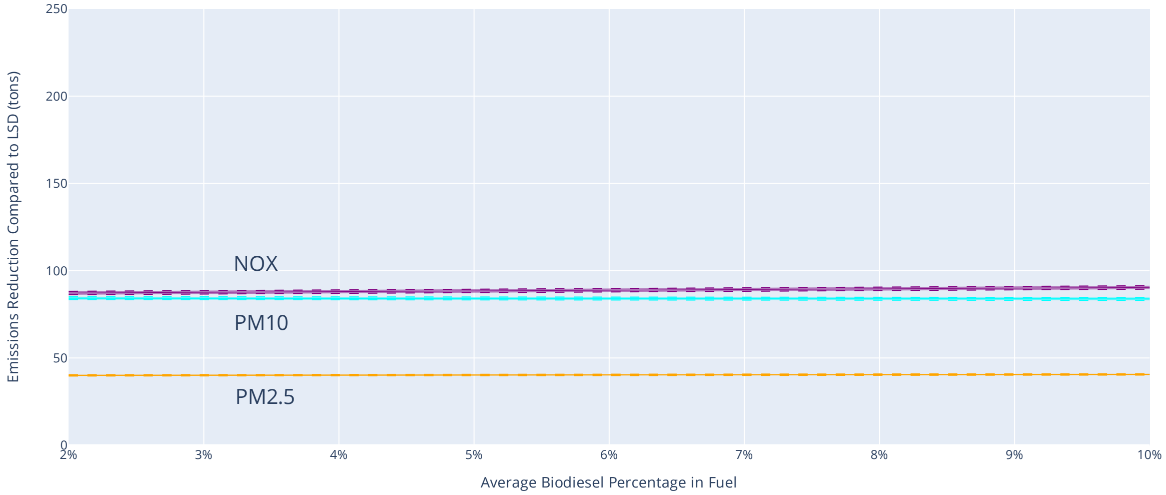

Figure S3.5x. Projected emissions reductions in 2029 for $PM_{2.5}$, PM_{10}, and NO_X at biodiesel blend volumes from 2% to 10% compared to the use of LSD for the Colorado diesel fleet bound by 5% and 95% confidence intervals.

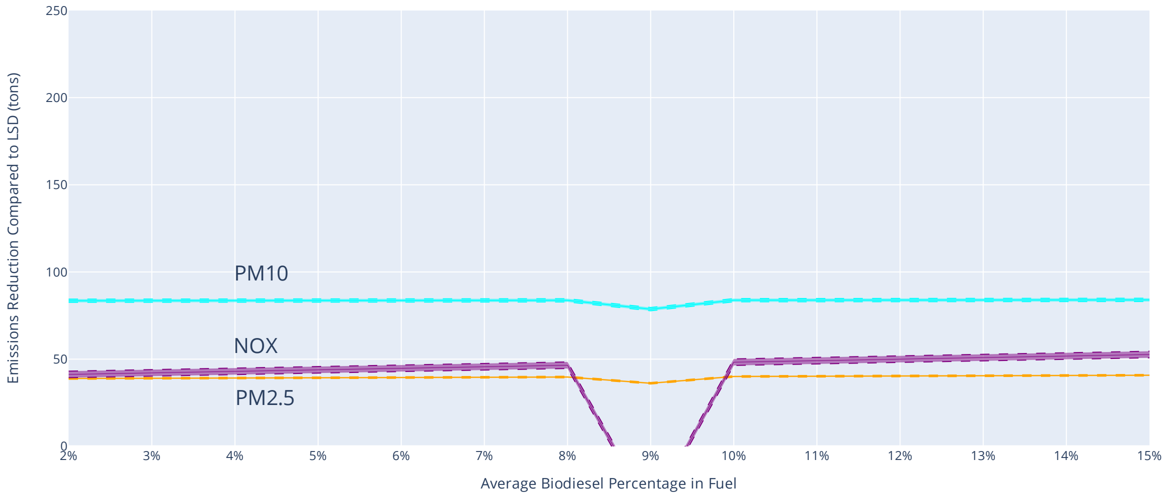

Figure S3.5y. Projected emissions reductions in 2030 for $PM_{2.5}$, PM_{10}, and NO_X at biodiesel blend volumes from 2% to 15% compared to the use of LSD for the Colorado diesel fleet bound by 5% and 95% confidence intervals. Note: An anomaly exists in GREET for a B9 blend in 2030

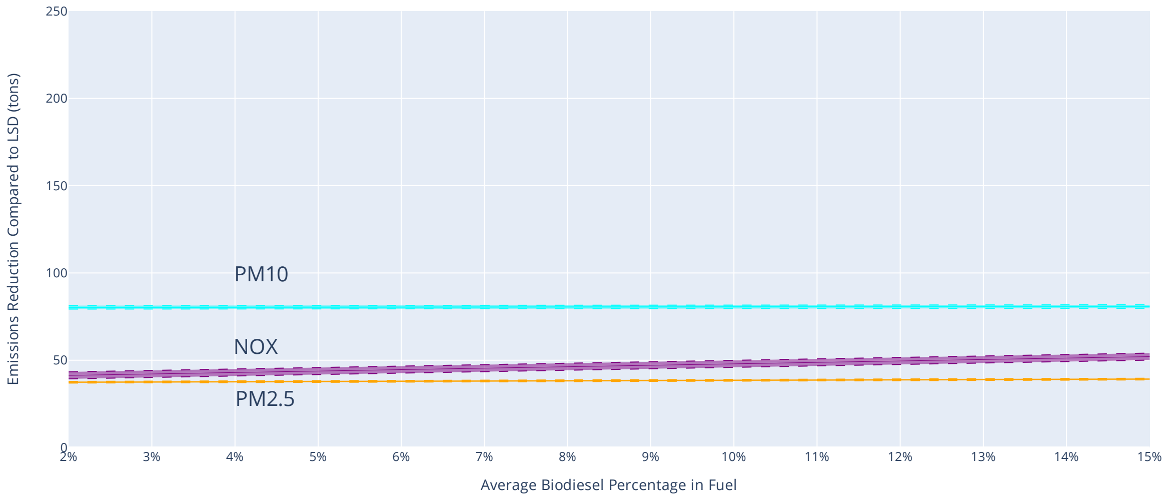

Figure S3.5z. Projected emissions reductions in 2031 for $PM_{2.5}$, PM_{10}, and NO_X at biodiesel blend volumes from 2% to 15% compared to the use of LSD for the Colorado diesel fleet bound by 5% and 95% confidence intervals.

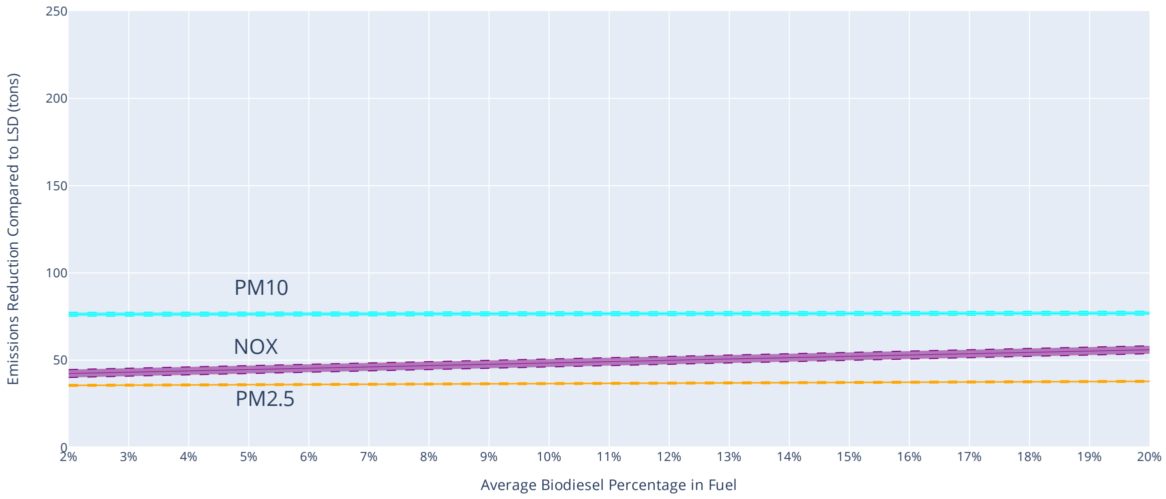

Figure S3.5aa. Projected emissions reductions in 2033 for $PM_{2.5}$, PM_{10}, and NO_X at biodiesel blend volumes from 2% to 15% compared to the use of LSD for the Colorado diesel fleet bound by 5% and 95% confidence intervals.

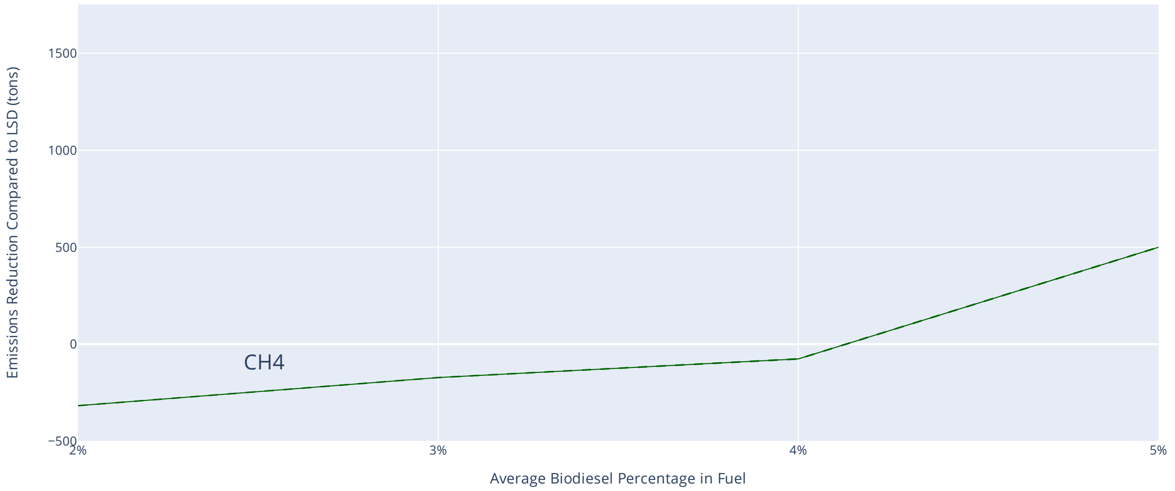

Figure S3.5bb. Projected emissions reductions in 2024 for CH_4 at biodiesel blend volumes from 2% to 5% compared to the use of LSD for the Colorado diesel fleet bound by 5% and 95% confidence intervals.

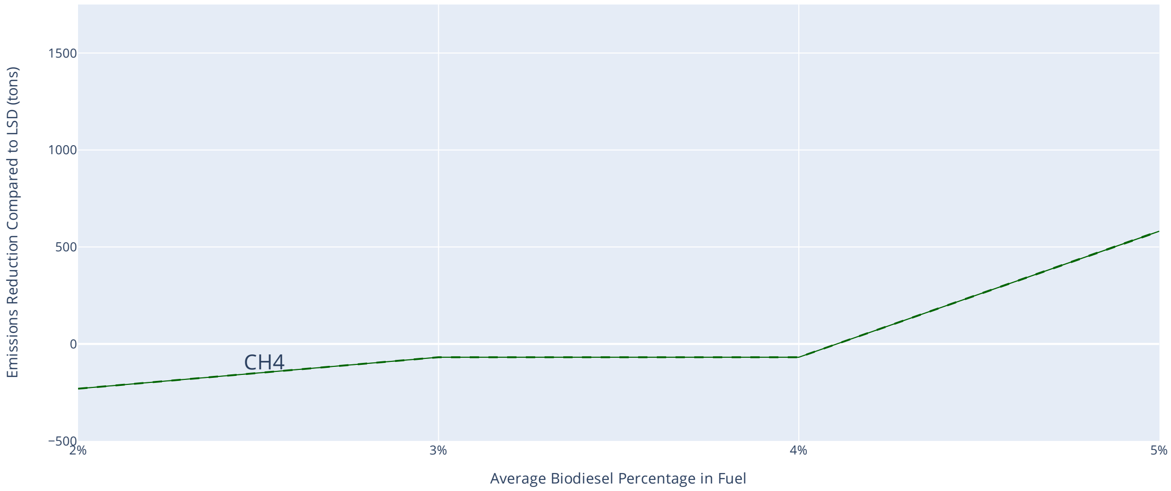

Figure S3.5cc. Projected emissions reductions in 2025 for CH_4 at biodiesel blend volumes from 2% to 5% compared to the use of LSD for the Colorado diesel fleet bound by 5% and 95% confidence intervals.

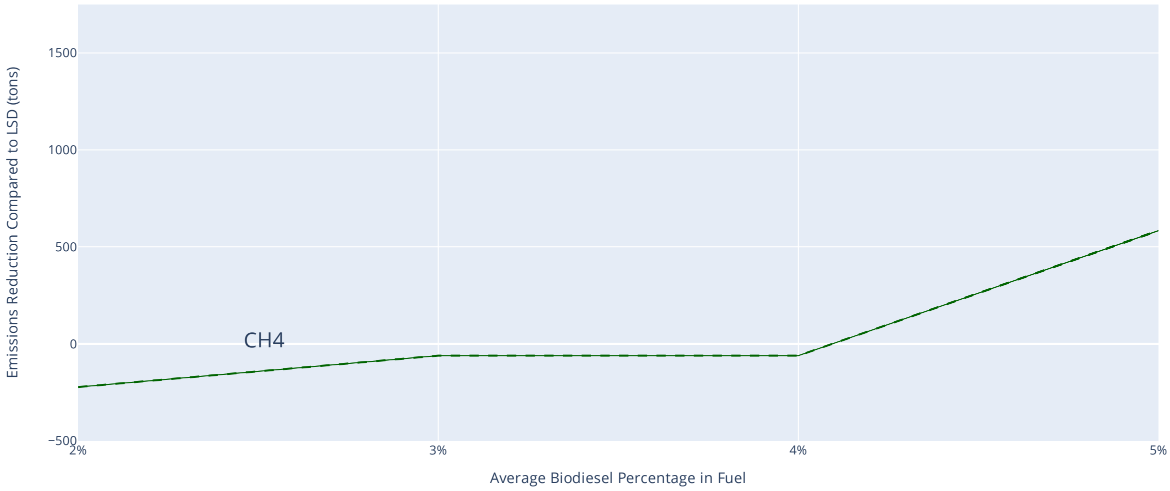

Figure S3.5dd. Projected emissions reductions in 2026 for CH_4 at biodiesel blend volumes from 2% to 5% compared to the use of LSD for the Colorado diesel fleet bound by 5% and 95% confidence intervals.

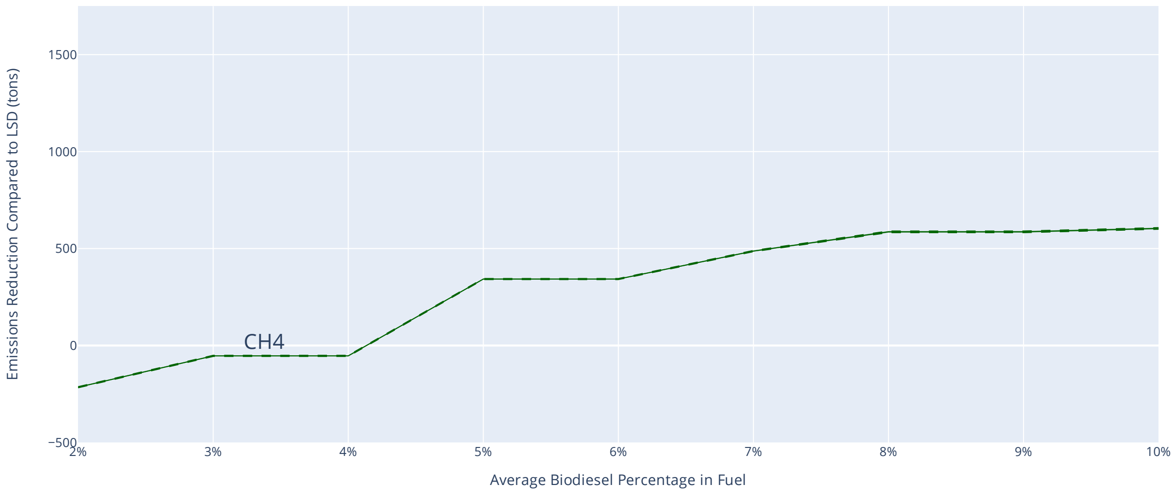

Figure S3.5ee. Projected emissions reductions in 2027 for CH_4 at biodiesel blend volumes from 2% to 10% compared to the use of LSD for the Colorado diesel fleet bound by 5% and 95% confidence intervals.

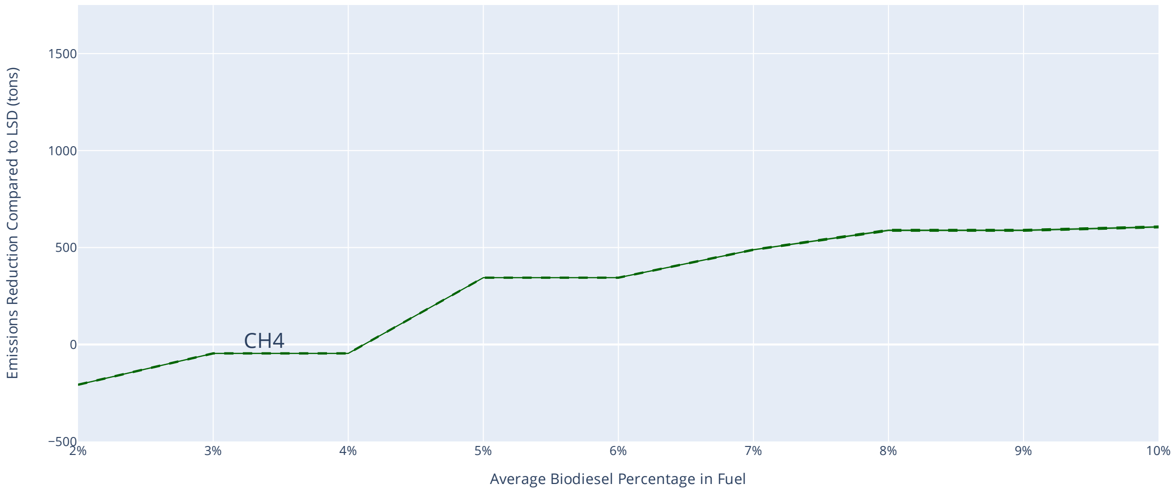

Figure S3.5ff. Projected emissions reductions in 2028 for CH_4 at biodiesel blend volumes from 2% to 10% compared to the use of LSD for the Colorado diesel fleet bound by 5% and 95% confidence intervals.

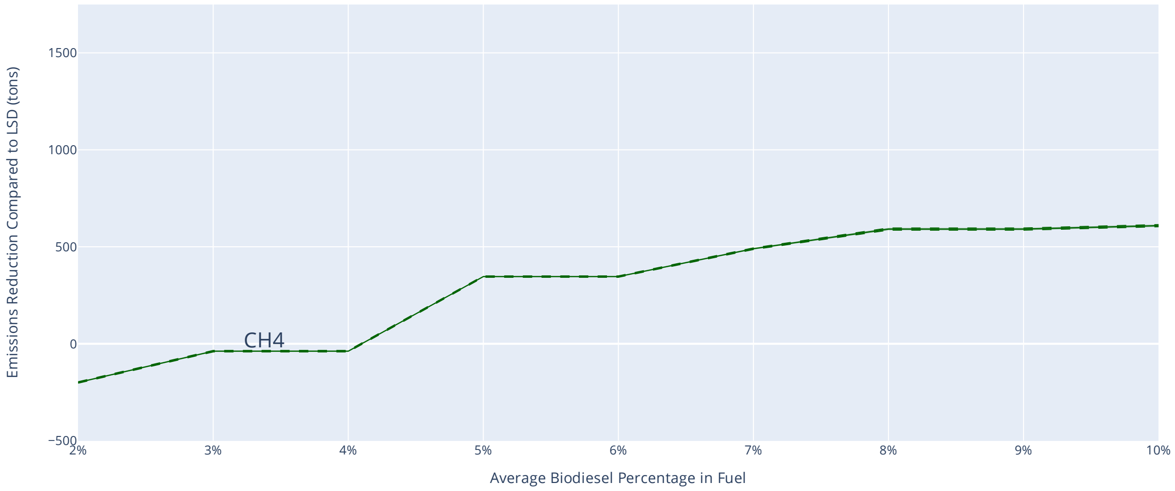

Figure S3.5gg. Projected emissions reductions in 2029 for CH_4 at biodiesel blend volumes from 2% to 10% compared to the use of LSD for the Colorado diesel fleet bound by 5% and 95% confidence intervals.

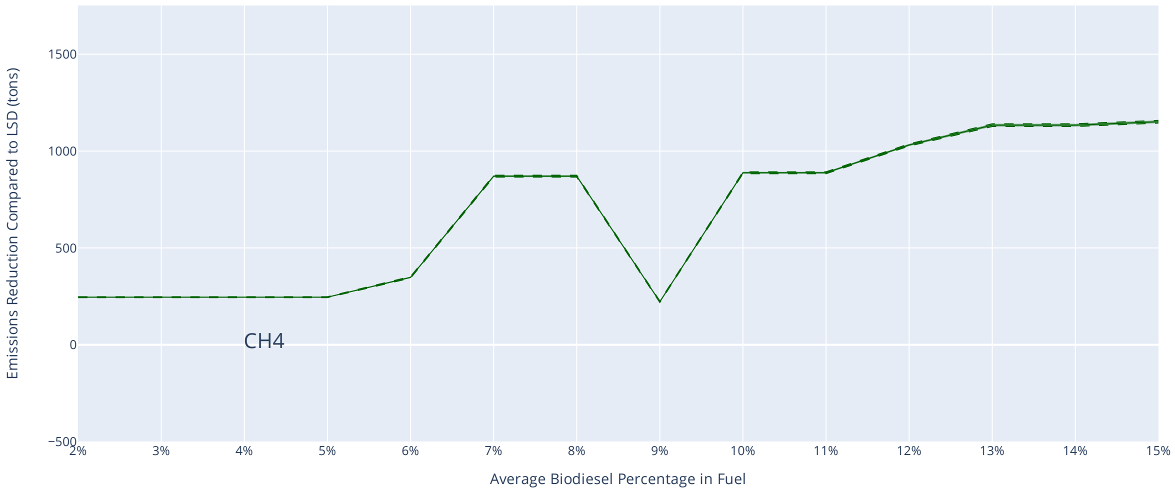

Figure S3.5hh. Projected emissions reductions in 2030 for CH_4 at biodiesel blend volumes from 2% to 15% compared to the use of LSD for the Colorado diesel fleet bound by 5% and 95% confidence intervals. Note: An anomaly exists in GREET for a B9 blend in 2030

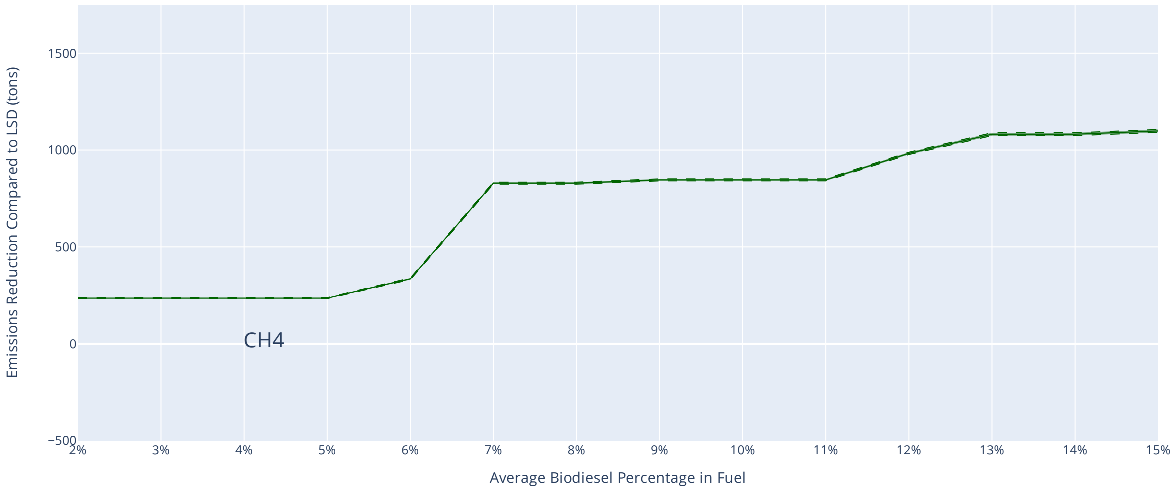

Figure S3.5ii. Projected emissions reductions in 2031 for CH_4 at biodiesel blend volumes from 2% to 15% compared to the use of LSD for the Colorado diesel fleet bound by 5% and 95% confidence intervals.

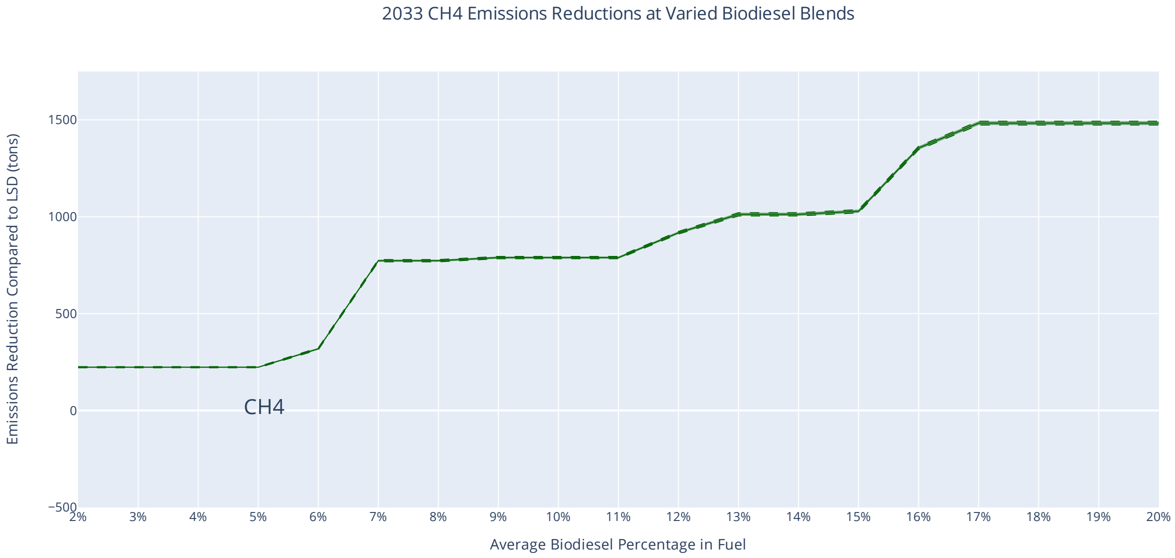

Figure S3.5jj. Projected emissions reductions in 2033 for CH_4 at biodiesel blend volumes from 2% to 20% compared to the use of LSD for the Colorado diesel fleet bound by 5% and 95% confidence intervals.

S3.7. Additional Social Cost/Benefit Figures

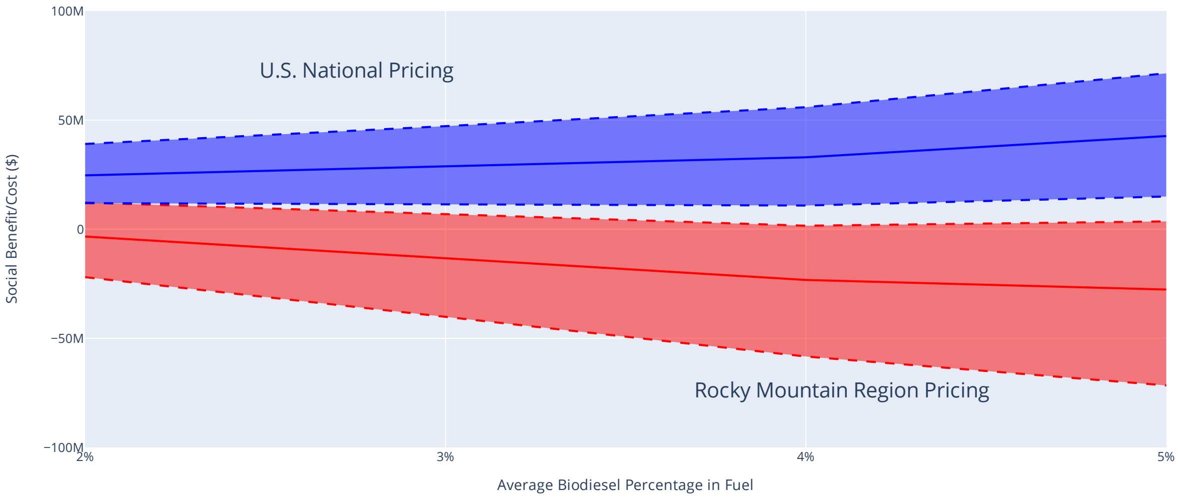

Figure S3.6a. Estimated social benefit/cost expected for biodiesel blend volumes from 2% to 5% compared to the use of LSD in 2024 by the Colorado diesel fleet bound by 5% and 95% confidence intervals for both Rocky Mountain Region and U.S. National Pricing data distributions. Positive vertical axis values are benefits while negative values are costs.

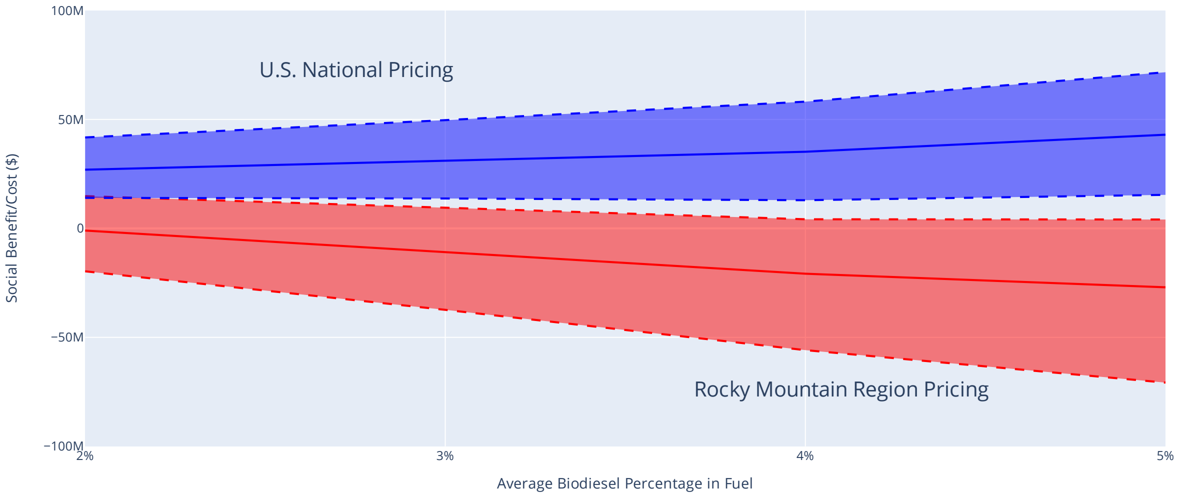

Figure S3.6b. Estimated social benefit/cost expected for biodiesel blend volumes from 2% to 5% compared to the use of LSD in 2025 by the Colorado diesel fleet bound by 5% and 95% confidence intervals for both Rocky Mountain Region and U.S. National Pricing data distributions. Positive vertical axis values are benefits while negative values are costs.

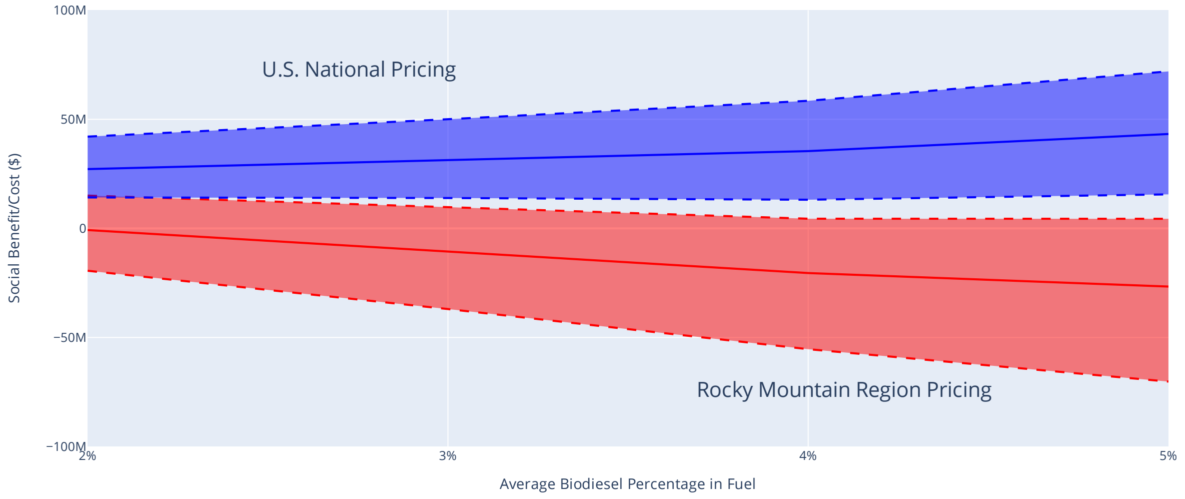

Figure S3.6c. Estimated social benefit/cost expected for biodiesel blend volumes from 2% to 5% compared to the use of LSD in 2026 by the Colorado diesel fleet bound by 5% and 95% confidence intervals for both Rocky Mountain Region and U.S. National Pricing data distributions. Positive vertical axis values are benefits while negative values are costs.

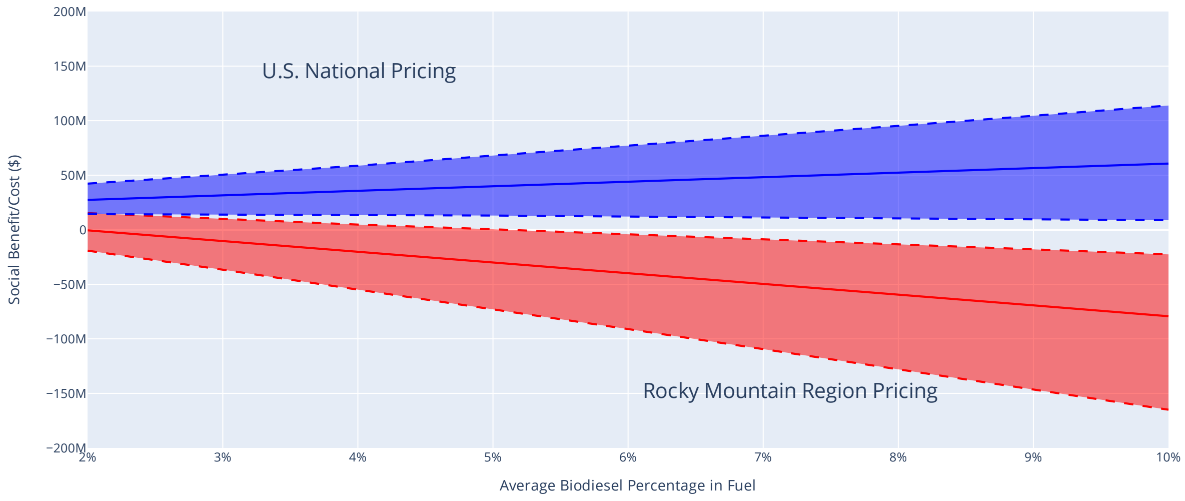

Figure S3.6d. Estimated social benefit/cost expected for biodiesel blend volumes from 2% to 10% compared to the use of LSD in 2027 by the Colorado diesel fleet bound by 5% and 95% confidence intervals for both Rocky Mountain Region and U.S. National Pricing data distributions. Positive vertical axis values are benefits while negative values are costs.

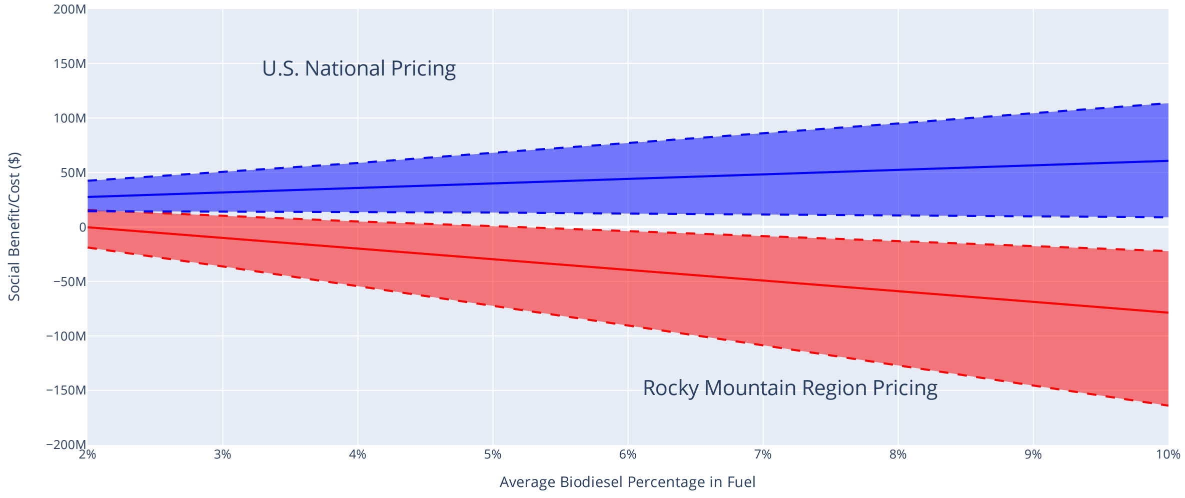

Figure S3.6e. Estimated social benefit/cost expected for biodiesel blend volumes from 2% to 10% compared to the use of LSD in 2028 by the Colorado diesel fleet bound by 5% and 95% confidence intervals for both Rocky Mountain Region and U.S. National Pricing data distributions. Positive vertical axis values are benefits while negative values are costs.

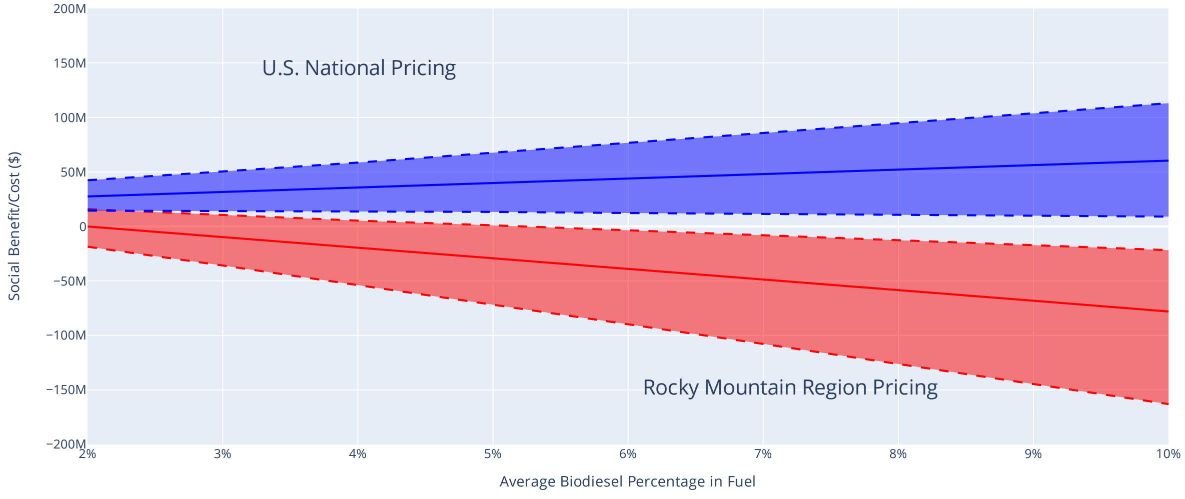

Figure S3.6f. Estimated social benefit/cost expected for biodiesel blend volumes from 2% to 10% compared to the use of LSD in 2029 by the Colorado diesel fleet bound by 5% and 95% confidence intervals for both Rocky Mountain Region and U.S. National Pricing data distributions. Positive vertical axis values are benefits while negative values are costs.

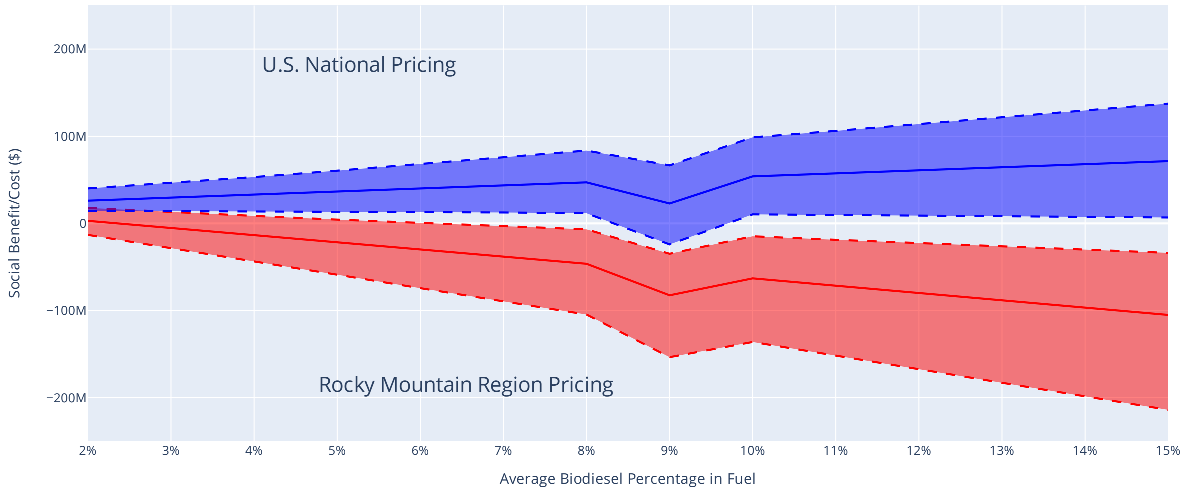

Figure S3.6g. Estimated social benefit/cost expected for biodiesel blend volumes from 2% to 15% compared to the use of LSD in 2030 by the Colorado diesel fleet bound by 5% and 95% confidence intervals for both Rocky Mountain Region and U.S. National Pricing data distributions. Positive vertical axis values are benefits while negative values are costs. Note: An anomaly exists in GREET for a B9 blend in 2030

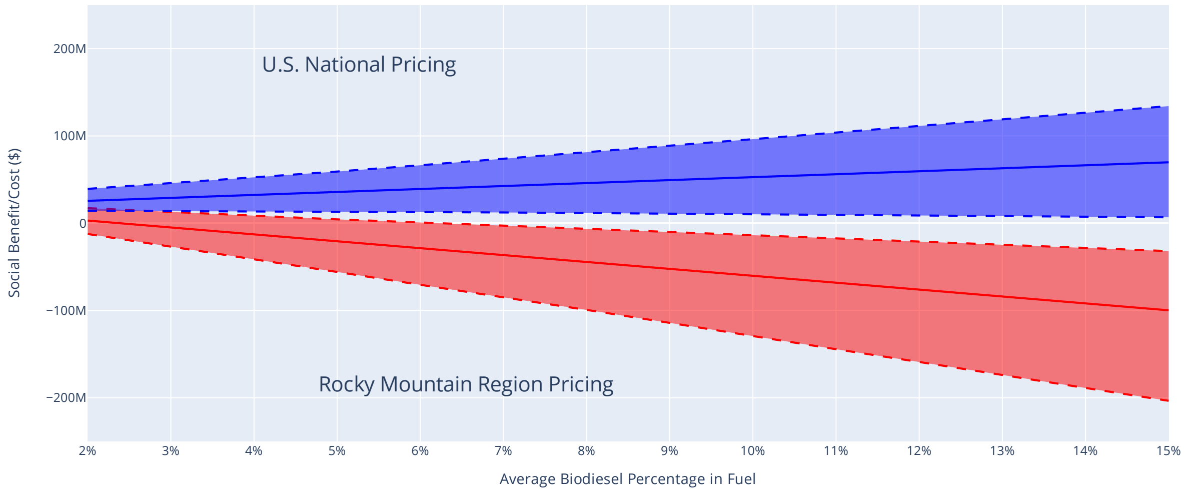

Figure S3.6h. Estimated social benefit/cost expected for biodiesel blend volumes from 2% to 15% compared to the use of LSD in 2031 by the Colorado diesel fleet bound by 5% and 95% confidence intervals for both Rocky Mountain Region and U.S. National Pricing data distributions. Positive vertical axis values are benefits while negative values are costs.

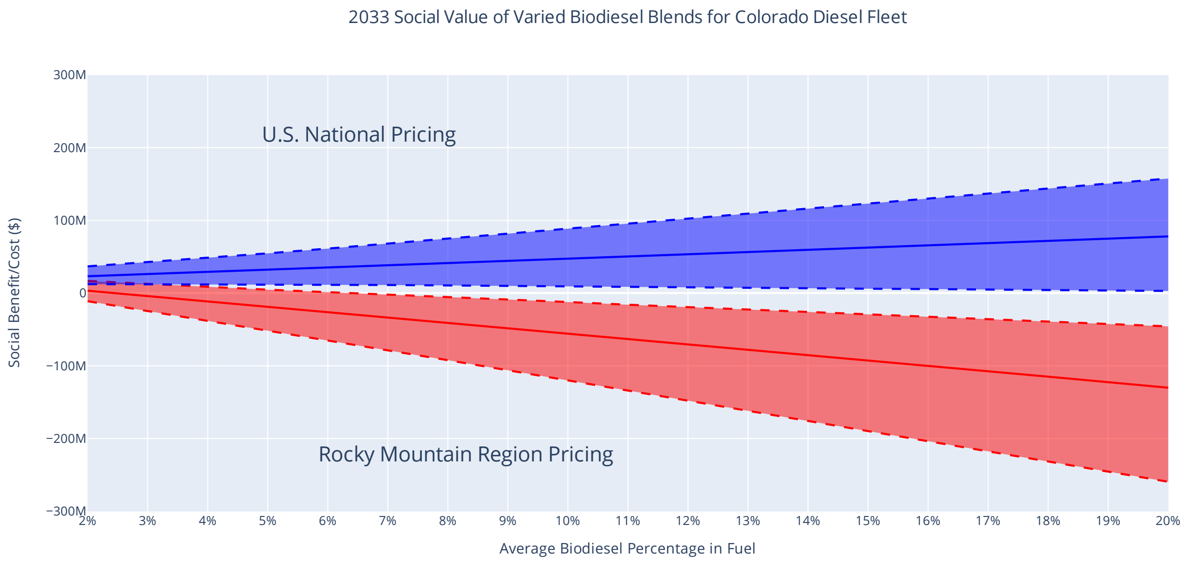

Figure S3.6i. Estimated social benefit/cost expected for biodiesel blend volumes from 2% to 20% compared to the use of LSD in 2033 by the Colorado diesel fleet bound by 5% and 95% confidence intervals for both Rocky Mountain Region and U.S. National Pricing data distributions. Positive vertical axis values are benefits while negative values are costs.

S3.7 Discussion on Potential of Biodiesel Production in Colorado

Cattle production is the largest agricultural industry in Colorado and 81% of all Colorado farms are family-run.[41] In 2021, 2.3 million cows were slaughtered in Colorado, of which 11% of the weight is beef tallow.[41,42] If all of the beef tallow produced in Colorado were converted to biodiesel, 48 million gallons of biodiesel could be produced supplying the state with a B8 equivalent volume [42]. At the time of this study, an unknown amount of current beef tallow produced in Colorado is not used for other purposes, so our biodiesel production value is an overestimate. Colorado is also the 17th largest corn producer at 123 million bushels in 2020.[41] Colorado has three bioethanol production facilities that produced 127 million gallons of ethanol in 2018.[43] Assuming 0.7 lbs of corn oil are produced for every 2.7 gallons of ethanol (the typical production from 1 bushel of corn), 7.7 lbs of corn oil are required to make 1 gallon of biodiesel, and all potentially produced corn oil in Colorado goes towards biodiesel production, this source could account for up to 4.2 million gallons of biodiesel annually, equivalent to a just under a B1 volume.[44] Additionally, Colorado has more than 12,700 restaurants (5th highest in restaurants per capita), generating an unquantified volume of waste cooking oil that could be collected and converted to biodiesel at a rate of 7.7 lbs of oil per gallon of biodiesel.[45, 46]

S3.8 Sensitivity Analysis Results

Table S3.8. Model Sensitivity Analysis Results

Parameter	Social Cost	CO_2 Emissions	$PM_{2.5}$ Emissions	PM_{10} Emissions	CO Emissions	VOC Emissions	NO_X Emissions	SO_X Emissions	CH_4 Emissions
Biodiesel Cost Difference	-0.9211	0.0016	0.0011	0.0011	0.0012	-0.0006	0.0006	0.0009	0.0026
Electrification CAGR	0.0048	0.0071	0.0078	0.0078	0.0079	0.0085	0.0022	0.0078	0.0070
CAGR of HD Fleet	-0.0063	0.3297	0.2928	0.2875	0.2948	0.1128	0.3127	0.2709	0.3754
CAGR of MD Fleet	0.0037	0.9289	0.9488	0.9507	0.9474	0.9909	0.6714	0.9554	0.8662
CAGR of Bus Fleet	-0.0006	0.0023	0.0056	0.0060	0.0073	0.0036	0.0044	0.0043	0.0217
CAGR of LD Fleet	-0.0129	-0.0096	0.0116	0.0136	0.0197	0.0043	-0.1147	0.0049	0.0682
HD Mileage Up to 2030	0.0511	-0.0060	-0.0060	-0.0060	-0.0059	-0.0052	-0.0050	-0.0060	-0.0056
HD Mileage After 2030	0.0653	-0.0088	-0.0093	-0.0094	-0.0094	-0.0100	-0.0031	-0.0093	-0.0092
MD Mileage Up to 2030	0.0128	0.0010	0.0015	0.0015	0.0016	0.0020	0.0001	0.0015	0.0015
MD Mileage After 2030	0.0399	0.0136	0.0133	0.0132	0.0133	0.0105	0.0109	0.0129	0.0150
Bus Mileage Up to 2030	0.0088	-0.0010	-0.0009	-0.0009	-0.0008	-0.0009	-0.0031	-0.0009	0.0005
Bus Mileage After 2030	0.0019	-0.0091	-0.0080	-0.0078	-0.0079	-0.0029	-0.0083	-0.0074	-0.0098
LD Mileage Up to 2030	0.0307	-0.0107	-0.0110	-0.0110	-0.0111	-0.0108	-0.0057	-0.0109	-0.0111
LD Mileage After 2030	0.0636	-0.0026	-0.0026	-0.0026	-0.0025	-0.0030	-0.0011	-0.0028	-0.0009
CO_2 Social Cost	0.2263	0.0011	0.0004	0.0003	0.0002	-0.0008	0.0021	0.0004	-0.0008
PM Social Cost	0.1074	-0.0074	-0.0073	-0.0073	-0.0075	-0.0042	-0.0048	-0.0069	-0.0090
CO Social Cost	0.0058	-0.0009	-0.0006	-0.0005	-0.0005	0.0008	-0.0024	-0.0005	-0.0006
VOC Social Cost	0.0536	0.0074	0.0073	0.0073	0.0073	0.0068	0.0078	0.0073	0.0067
NO_X Social Cost	0.0174	0.0133	0.0133	0.0133	0.0134	0.0119	0.0097	0.0131	0.0136
SO_X Social Cost	0.0562	-0.0189	-0.0186	-0.0185	-0.0185	-0.0175	-0.0156	-0.0185	-0.0180
CH_4 Social Cost	0.0023	0.0194	0.0195	0.0195	0.0195	0.0190	0.0142	0.0195	0.0186

REFERENCES

(1) Hidy, G. M.; Heisler, S. L.; Watson, J. G.; Wolff, G. T. Winter Urban Chemistry and Denver's Brown Cloud: Part 1—Light Extinction and Visibility. *Aerosol Sci. Eng.* **2020**, *4* (2), 64–79. https://doi.org/10.1007/s41810-020-00055-5.

(2) Reşitoğlu, İ. A.; Altinişik, K.; Keskin, A. The Pollutant Emissions from Diesel-Engine Vehicles and Exhaust Aftertreatment Systems. *Clean Technol. Environ. Policy* **2015**, *17* (1), 15–27. https://doi.org/10.1007/s10098-014-0793-9.

(3) U.S. EPA. *COLORADO: Denver Metro/North Front Range Nonattainment Area Intended Area Designations for the 2015 Ozone National Ambient Air Quality Standards Technical Support Document (TSD) for Counties Remanded to EPA*; 2021. https://www.epa.gov/sites/default/files/2021-05/documents/final_clean_co_tsd_-_weld_county_remand.pdf.

(4) U.S. EPA. *Determinations of Attainment by the Attainment Date, Extension of the Attainment Date, and Reclassification of Areas Classified as Serious for the 2008 Ozone National Ambient Air Quality Standards*; Proposed Rule EPA-HQ-OAR-2021-0741; U.S. EPA, 2022; pp 21825–21842.

(5) Miller, Faith. Colorado Could See $43M More for Air-Quality Monitoring, Electric Lawn Equipment Rebates. *Colorado Newsline*. Colorado April 1, 2022. https://coloradonewsline.com/2022/04/01/cdphe-43-million-air-quality-monitoring/ (Date last accessed 2024-06-16).

(6) A. Sydbom; A. Blomberg; S. Parnia; N. Stenfors; T. Sandström; S-E. Dahlén. Health Effects of Diesel Exhaust Emissions. *Eur. Respir. J.* **2001**, *17* (4), 733. https://doi.org/10.1183/09031936.01.17407330.

(7) GBD 2019 Risk Factors Collaborators. Global Burden of 87 Risk Factors in 204 Countries and Territories, 1990-2019: A Systematic Analysis for the Global Burden of Disease Study 2019. *Lancet Lond. Engl.* **2020**, *396* (10258), 1223–1249. https://doi.org/10.1016/S0140-6736(20)30752-2.

(8) ICF Resources. *Low Carbon Fuel Standard Feasibility Study Final Report*; Colorado Energy Office, 2020. https://drive.google.com/file/d/11zczj8ieUzNbxMvlob9HJCctyzJGVYF3/view?usp=embed_facebook (Date last accessed 2023-02-09).

(9) Moynihan, P.; Culkin, J.; Seamonds, D.; Huntington, A.; MacIntosh, R. *Colorado Medium- and Heavy- Duty (M/HD) Vehicle Study*; Colorado Energy Office, 2021.

(10) Bourbon, E.; Science, A. Clean Cities Alternative Fuel Price Report, April 2022. **2022**.

(11) Dunlap, R. A. Biofuels. In *Transportation Technologies for a Sustainable Future*; IOP Publishing, 2023; pp 4–1. https://doi.org/10.1088/978-0-7503-5306-9ch4.

(12) Alleman, T. L.; McCormick, R. L.; Christensen, E. D.; Fioroni, G.; Moriarty, K.; Yanowitz, J. *Biodiesel Handling and Use Guide (Fifth Edition)*; United States, 2016.

(13) Ledna, C.; Muratori, M.; Yip, A.; Jadun, P.; Hoehne, C. *Decarbonizing Medium-& Heavy-Duty On-Road Vehicles: Zero-Emission Vehicles Cost Analysis*; National Renewable Energy Lab.(NREL), Golden, CO (United States), 2022.

(14) Somers, M.; Batan, L.; Al-Alawi, B.; Bradley, T. H. A Colorado-Specific Life Cycle Assessment Model to Support Evaluation of Low-Carbon Transportation Fuels and Policy. *Environ. Res. Infrastruct. Sustain.* **2022**, *2* (1), 011001.

(15) Ghadikolaei, M. A.; Wong, P. K.; Cheung, C. S.; Zhao, J.; Ning, Z.; Yung, K.-F.; Wong, H. C.; Gali, N. K. Why Is the World Not yet Ready to Use Alternative Fuel Vehicles? *Heliyon* **2021**, *7* (7), e07527. https://doi.org/10.1016/j.heliyon.2021.e07527.
(16) Interagency Working Group on Social Cost of Greenhouse Gases. *Technical Support Document: Social Cost of Carbon, Methane, and Nitrous Oxide Nterim Estimates under Executive Order 13990*; United States Government, 2021.
(17) IRENA. *The True Cost of Fossil Fuels: Externality Cost Assessment Methodology*; 2016.
(18) Song, S. *Transport Emissions & Social Cost Assessment: Methodology Guide*; World Resources Insitute, 2016.
(19) U.S. Department of Energy. *Clean Cities Alternative Fuel Price Report, July 2022*; U.S. Department of Energy, 2022. https://afdc.energy.gov/files/u/publication/alternative_fuel_price_report_july_2022.pdf.
(20) U.S. Department of Energy. *Clean Cities Alternative Fuel Price Report, April 2022*; U.S. Department of Energy, 2022. https://afdc.energy.gov/files/u/publication/alternative_fuel_price_report_april_2022.pdf.
(21) U.S. Department of Energy. *Clean Cities Alternative Fuel Price Report, January 2022*; U.S. Department of Energy, 2022. https://afdc.energy.gov/files/u/publication/alternative_fuel_price_report_january_2022.pdf.
(22) U.S. Department of Energy. *Clean Cities Alternative Fuel Price Report, October 2021*; U.S. Department of Energy, 2021. https://afdc.energy.gov/files/u/publication/alternative_fuel_price_report_october_2021.pdf.
(23) U.S. Department of Energy. *Clean Cities Alternative Fuel Price Report, July 2021*; U.S. Department of Energy, 2021. https://afdc.energy.gov/files/u/publication/alternative_fuel_price_report_july_2021.pdf.
(24) U.S. Department of Energy. *Clean Cities Alternative Fuel Price Report, April 2021*; U.S. Department of Energy, 2021. https://afdc.energy.gov/files/u/publication/alternative_fuel_price_report_april_2021.pdf.
(25) U.S. Department of Energy. *Clean Cities Alternative Fuel Price Report, January 2021*; U.S. Department of Energy, 2021. https://afdc.energy.gov/files/u/publication/alternative_fuel_price_report_january_2021.pdf.
(26) U.S. Department of Energy. *Clean Cities Alternative Fuel Price Report, October 2020*; U.S. Department of Energy, 2020. https://afdc.energy.gov/files/u/publication/alternative_fuel_price_report_october_2020.pdf.
(27) U.S. Department of Energy. *Clean Cities Alternative Fuel Price Report, July 2020*; U.S. Department of Energy, 2020. https://afdc.energy.gov/files/u/publication/alternative_fuel_price_report_july_2020.pdf.
(28) U.S. Department of Energy. *Clean Cities Alternative Fuel Price Report, April 2020*; U.S. Department of Energy, 2020. https://afdc.energy.gov/files/u/publication/alternative_fuel_price_report_april_2020.pdf.
(29) U.S. Department of Energy. *Clean Cities Alternative Fuel Price Report, January 2020*; U.S. Department of Energy, 2020. https://afdc.energy.gov/files/u/publication/alternative_fuel_price_report_jan_2020.pdf.
(30) U.S. Department of Energy. *Clean Cities Alternative Fuel Price Report, October 2019*; U.S. Department of Energy, 2019. https://afdc.energy.gov/files/u/publication/alternative_fuel_price_report_oct_2019.pdf.

(31) U.S. Department of Energy. *Clean Cities Alternative Fuel Price Report, July 2019*; U.S. Department of Energy, 2019. https://afdc.energy.gov/files/u/publication/alternative_fuel_price_report_july_2019.pdf.
(32) U.S. Department of Energy. *Clean Cities Alternative Fuel Price Report, April 2019*; U.S. Department of Energy, 2019. https://afdc.energy.gov/files/u/publication/alternative_fuel_price_report_april_2019.pdf.
(33) U.S. Department of Energy. *Clean Cities Alternative Fuel Price Report, January 2019*; U.S. Department of Energy, 2019. https://afdc.energy.gov/files/u/publication/alternative_fuel_price_report_jan_2019.pdf.
(34) U.S. Department of Energy. *Clean Cities Alternative Fuel Price Report, October 2018*; U.S. Department of Energy, 2018. https://afdc.energy.gov/files/u/publication/alternative_fuel_price_report_oct_2018.pdf.
(35) U.S. Department of Energy. *Clean Cities Alternative Fuel Price Report, July 2018*; U.S. Department of Energy, 2018. https://afdc.energy.gov/files/u/publication/alternative_fuel_price_report_july_2018.pdf.
(36) U.S. Department of Energy. *Clean Cities Alternative Fuel Price Report, April 2018*; U.S. Department of Energy, 2018. https://afdc.energy.gov/files/u/publication/alternative_fuel_price_report_april_2018.pdf.
(37) U.S. Department of Energy. *Clean Cities Alternative Fuel Price Report, January 2018*; U.S. Department of Energy, 2018. https://afdc.energy.gov/files/u/publication/alternative_fuel_price_report_jan_2018.pdf.
(38) U.S. Department of Energy. *Clean Cities Alternative Fuel Price Report, October 2017*; U.S. Department of Energy, 2017. https://afdc.energy.gov/files/u/publication/alternative_fuel_price_report_oct_2017.pdf.
(39) U.S. Department of Energy. *Clean Cities Alternative Fuel Price Report, July 2017*; U.S. Department of Energy, 2017. https://afdc.energy.gov/files/u/publication/alternative_fuel_price_report_july_2017.pdf.
(40) U.S. Department of Energy. *Clean Cities Alternative Fuel Price Report, April 2017*; 2017. https://afdc.energy.gov/files/u/publication/alternative_fuel_price_report_april_2017.pdf.
(41) United States Department of Agriculture; National Agricultural Statistics Service; Mountain Region, Colorado Field Office. *Colorado Agricultural Statistics 2021*; USDA, 2022.
(42) Nelson, R. G.; Schrock, M. D. Energetic and Economic Feasibility Associated with the Production, Processing, and Conversion of Beef Tallow to a Substitute Diesel Fuel. *Biomass Bioenergy* **2006**, *30* (6), 584–591. https://doi.org/10.1016/j.biombioe.2005.09.005.
(43) U.S. EIA. *U.S. Fuel Ethanol Plant Production Capacity*. https://www.eia.gov/petroleum/ethanolcapacity/ (Date last accessed 2023-02-09).
(44) McAloon, A.; Taylor, F.; Yee, W. *Determining the Cost of Producing Ethanol from Corn Starch and Lignocellulosic Feedstocks*; NREL, 2000.
(45) Colorado Restaurant Association. *Restaurant Industry Statistics - CO Restaurant Association*. https://corestaurant.org/industry-statistics/ (Date last accessed 2023-02-09).
(46) Brorsen, W. Projections of US Production of Biodiesel Feedstock. *Int. Counc. Clean Transp.* **2015**, 1–13.
